# Full-Cycle Ecological Damage Assessment Framework for Sudden Water Pollution Accidents: Multi-Model Coupled Prediction and Three-Dimensional Quantitative Evaluation with a Case Study of Tailings Dam Breach

**DOI:** 10.3390/toxics14090745

**Published:** 2026-08-23

**Authors:** Zhengda Lin, Xinhao Sun, Bingjie Yan, Caoqingqing Li

**Affiliations:** 1 State Key Laboratory of Urban Water Resource and Environment, School of Environment, Harbin Institute of Technology, Harbin 150090, China; sunxinhao2001@163.com (X.S.); 18654872775@163.com (B.Y.); 2 Heilongjiang Research Academy of Environmental Sciences, No. 3 Haixing Street, Nangang District, Harbin 150090, China

**Keywords:** heavy metal pollution, ecological damage comprehensive assessment, pollutant diffusion prediction, graph theory model, ecosystem service loss

## Abstract

Sudden tailings dam breaches trigger large-scale heavy metal compound pollution in coupled surface water–groundwater systems, requiring systematic full-cycle ecological damage quantification tools applicable to diverse contamination types. This study constructs an integrated full-cycle ecological damage assessment framework for sudden water pollution accidents, integrating three core modules: multi-model pollutant migration prediction, multi-scale aquatic biological damage diagnosis, and three-dimensional ecological-economic loss accounting. The framework adopts a modular design that can potentially accommodate heavy metals (Cd, Cr, As, Pb) and organic pollutants such as polycyclic aromatic hydrocarbons (PAHs), with standardized molecular, individual, and population-level biological endpoints and corresponding pollutant dose–response templates reserved as reference calculation modules. However, applicability beyond this case has not been validated and requires case-specific calibration. To verify the operability and accuracy of the proposed integrated system, a typical tailings dam leakage incident dominated by hexavalent chromium (Cr(VI)) and arsenic (As) pollution was selected as the practical validation case; all field monitoring, pollutant simulation, and final economic loss quantification in this case exclusively rely on on-site measured Cr(VI) and As data, while Cd and PAH-related biological response curves and remediation cost formulas retained in the manuscript only serve as illustrative universal template components of the framework rather than case-measured results. For the Cr(VI)/As pollution case, the advection–diffusion model simulation revealed that the Cr(VI) contamination plume horizontally spread 250 m within 48 h and extended to 560 m after seven days, and anaerobic groundwater environments drove the transformation of toxic mobile trivalent arsenic (As(III)) from primary pentavalent arsenic. The calibrated SWAT model achieved Nash–Sutcliffe efficiency (NSE) coefficients of 0.75 for dissolved Cr(VI) and 0.68 for particulate As. The graph theory-based rapid prediction model cut computation duration down to minutes; when validated against independent field monitoring data, it yielded an average relative error of 14.2%, and its consistency with the SWAT model reached 10.5% relative deviation, satisfying the accuracy requirement for emergency early warning. Field biological monitoring demonstrated substantial ecological impairment: metallothionein (MT) expression in fish tissues was markedly elevated (the reported 6.2-fold induction value derives from standard Cd exposure template tests within the framework, with analogous MT upregulation also observed for field Cr(VI)/As co-stress), and benthic community Shannon diversity declined by over 50% in polluted river reaches. The standardized Ecological Damage Index (EDI) of the case was calculated as 480.2, indicating severe aquatic ecosystem damage, with total comprehensive ecological and economic losses reaching 17.25 million CNY. This study innovatively couples high-precision physical transport models with fast emergency prediction algorithms and establishes a complete multi-tier biological indicator chain linking molecular biomarkers to community integrity metrics; the three-dimensional loss accounting system integrating ecosystem service impairment, restoration expenditure, and post-pollution recovery loss realizes closed-loop full-cycle damage evaluation. The proposed framework, demonstrated for Cr(VI) and As pollution, has a modular design that may potentially be extended to other pollutants such as Cd and PAHs by adjusting model parameters, providing a quantitative reference for emergency disposal, pollution remediation, and ecological compensation of water contamination accidents, although further validation across different pollutants and hydrological settings is required.

## 1. Introduction

With the rapid development of the global economy and the advancement of industrialization, human activities have caused intensifying interference with the environment. As one of the critical environmental carriers, the aquatic environment is facing severe pollution pressure. A substantial amount of heavy metal elements enter surface water bodies, groundwater, sediments, and organisms through various pathways, including industrial discharges, mining activities, agricultural runoff, and domestic wastewater, leading to complex interconnected ecological, chemical, and biological issues [1]. Heavy metals possess fundamentally different environmental behaviors compared to organic pollutants. As inorganic elements, they cannot be microbially or chemically degraded into harmless end-products in environmental media. Instead, they persist indefinitely and can only undergo transformations in speciation, valence states, or partitioning among dissolved, particulate, and sedimentary phases. This inherent non-degradability and environmental persistence cause heavy metals to readily accumulate in soils, sediments, and biota, with the potential for transfer and biomagnification along food chains, thereby posing long-term risks to aquatic ecosystem stability and human health [2]. It should be noted that while the title and subsequent sections of this paper focus specifically on chromium (Cr) and arsenic (As), these two elements are not the only, nor the most abundant, heavy metal pollutants in aquatic environments on a global scale. In terms of occurrence and concentration levels in surface water and sediment, comprehensive synthesis studies have identified manganese (Mn), zinc (Zn), copper (Cu), and lead (Pb) as the most prevalent heavy metals across regional and continental water systems. Chromium and arsenic, the subjects of this study, merit focused investigation not because they are the most common or abundant heavy metals, but because their unique speciation-dependent toxicity, redox-sensitive mobility, and contrasting environmental behaviors under varying geochemical conditions present particularly complex scientific challenges for understanding heavy metal fate and transport in aquatic systems. This study specifically focuses on hexavalent chromium [Cr(VI)] and arsenic [As], which are the characteristic pollutants of tailings dam breach accidents. Cr(VI) has strong oxidizing properties, high mobility and carcinogenicity, and can rapidly diffuse in groundwater and surface water systems. As has complex valence transformation characteristics, and pentavalent arsenic [As(V)] will be reduced to highly toxic and more mobile trivalent arsenic [As(III)] in anaerobic environments, leading to long-term ecological risks [3]. The environmental behaviors and toxic effects of these two pollutants are significantly different, so it is necessary to conduct separate simulation and assessment. From an ecosystem perspective, the distribution and migration of heavy metals in water bodies are governed by their distinct chemical properties and biological roles. Certain metals, such as iron, manganese, and zinc, act as essential micronutrients for organisms at low concentrations but become toxic when excessive, whereas non-essential metals like chromium and arsenic exert toxicity even at trace levels. Furthermore, several toxic metals and metalloids commonly occur in anionic forms, and their speciation, solubility, and bioavailability are highly sensitive to ambient redox conditions through oxidation or reduction. Heavy metals accumulate in organisms including benthic invertebrates, plankton, fish, and shellfish through direct uptake and trophic transfer, yet this process is not a simple uniform enrichment; it is substantially modulated by chemical speciation, biotransformation, and the contrasting degrees of biological essentiality. These complexities in metal behavior along the food chain ultimately pose potential risks to human health [4,5,6]. It is worth emphasizing that in recent years, under circumstances of sudden environmental incidents, including tailings dam failures and chemical plant accident releases, heavy metals have posed a new challenge to aquatic ecosystems due to their high release rates, extensive dispersion, and significant ecological impacts [7,8]. Therefore, developing scientific prediction models for heavy metal pollution dispersion and ecological damage assessment is not only a focus area in environmental research, but also serves as essential technical support urgently needed in ecological environment management, pollution prevention and control, and ecological compensation practices [9,10,11]. Against this backdrop, this study takes the Cr(VI) and As composite pollution caused by a typical tailings dam breach accident as the research object, aiming to clarify the migration and transformation laws of Cr(VI) and As in groundwater–surface water systems, verify the prediction accuracy of different models for these two pollutants, and quantify their multi-scale ecological damage. This study will bridge existing research gaps through a multi-model coupling and three-dimensional assessment framework, thereby providing scientific support for emergency response and ecological compensation of sudden Cr(VI) and As pollution accidents.

While the research on heavy metal pollution has accumulated a substantial body of literature, several critical issues remain to be addressed in areas such as pollutant diffusion prediction, quantification of ecological damages, remediation assessment, and decision support. Olha Biedunkova and Pavlo Kuznietsov [12] employed heavy metal pollution indices and statistical correlation analysis to assess heavy metal pollution in the Styr River. In practical applications, such data dependencies are often difficult to meet, particularly during unexpected incidents where data collection is characterized by delays and incomplete spatial coverage, thereby compromising the timeliness and accuracy of model predictions [12]. At the level of ecological damage assessment, current research mainly focuses on a single or a few isolated biological indicators, such as molecular biomarkers, individual-level toxicity indicators, or community-level characteristics, and lacks a systematic integration of multi-level biological factors including molecules, individuals, populations, and ecosystems. Zhang et al. [13] systematically analyzed the chronic risks of copper, zinc, and nickel to aquatic organisms in the Songhua River by examining community characteristics [13]. At the level of remediation technologies and environmental management, although various physical, chemical, and biological remediation methods have been proposed and studied, they often have their respective focuses and are mostly targeted at single pollutants or single pollution pathways. Kumar et al. [14] employed transgenic plants and nanomaterials for the efficient bioremediation of cadmium contamination, demonstrating the potential of integrated biotechnology in the treatment of individual heavy metals [14]. However, when facing complex composite pollution scenarios in aquatic environments, there is a lack of systematic, rapidly responsive assessment and remediation strategies.

To cope with the above challenges, researchers in recent years have carried out improvement work from multiple dimensions. The idea of multi-model coupling has been increasingly emphasized for the research of pollution dispersion prediction [15]. Multi-model coupling refers to the strategy of integrating two or more distinct models—each originally designed to represent specific physical, chemical, or biological processes—through data exchange and process feedback at their interfaces, thereby collectively simulating a system that none of the individual models can adequately describe alone. For instance, some studies have coupled convection-diffusion models with water runoff models or combined watershed models with spatial statistical models to enhance the applicability and prediction accuracy of the models for pollution transport under complex hydrological–geological conditions [16]. The practical significance of this approach lies in its ability to overcome the inherent limitations of single-model frameworks. A convection-diffusion model excels at resolving pollutant transport mechanisms but cannot independently generate the spatiotemporally varying flow fields that drive such transport, while a hydrological model provides distributed runoff and recharge estimates yet lacks the capacity to simulate pollutant fate at fine resolution. By coupling them, each model contributes its specialized strength to compensate for the others’ weaknesses, resulting in a predictive system that captures both the watershed-scale hydrological forcing and the local-scale reactive transport dynamics—a capability that is critically important for sudden pollution incidents where contaminant migration is simultaneously controlled by surface-runoff generation, groundwater recharge fluctuations, and in-stream attenuation processes. This complementarity makes multi-model coupling convincingly more appropriate than single-model approaches for complex environmental pollution scenarios. Jiayi Deng et al. [17] used a three-dimensional coupled geologic-groundwater flow model with the Indicator Kriging spatial statistical method to systematically simulate the migration paths of pollutants in karstic fissure media and complete the health risk zoning [17]. Meanwhile, some studies also combined the models with optimization algorithms or machine learning methods to improve the efficiency of model operation and prediction performance [18]. Hatice Kara [19] used machine learning algorithms such as Random Forest and Support Vector Machines to predict the index of heavy metal contamination in groundwater in order to assess the level of contamination and to identify the key influencing factors [19]. For studies in ecological damage assessment, researchers began to propose and apply multi-level and multi-indicator assessment systems [20]. Ecological risk assessment methods have also begun to be widely applied in the study of heavy metals in water bodies and sediments [21]. Xiang et al. [22] conducted an in-depth ecological risk assessment of a decommissioned industrial park, revealing that heavy metals such as Cd have high potential ecological risks due to the large proportion of highly reactive forms and strong mobility [22]. At the level of remediation technologies, physical, chemical, and bioremediation methods have been optimized and combined. Physical or chemical approaches usually use techniques such as membrane separation, in-situ permeation reaction barriers for remediation of aqueous environments. Li et al. [23] used layered double hydroxides based on the memory effect for highly efficient mineralization and electrochemical recovery of Pb ions from aqueous environments [23]. Bioremediation approaches usually use phytoremediation and biochar combined with microbial systems. Jonas Bayuo et al. systematically elucidated the use of green technology for the remediation of heavy metals in wastewater through phytoextraction, inter-root filtration, and other mechanisms using a variety of aquatic plants such as balsam, water hyacinth, and duckweed, and analyzed its advantages, challenges, and future development directions [24].

Further comparative analysis with classic global tailings dam failure events can reflect the advantages and practical value of the integrated assessment framework proposed in this study. The 2015 Fundão tailings dam collapse in Brazil caused large-scale iron oxide sediment pollution, and the post-disaster assessment only adopted a single sediment heavy metal risk model without coupling groundwater–surface water multi-medium migration simulation [25]. The 1998 Aznalcóllar mine leakage accident in Spain involved multiple metal pollutants, but only static post-hoc economic loss calculation was carried out, lacking real-time rapid prediction tools for emergency response [26]. The Xiangfen tailings leakage accident in China only adopted single water quality indicators for pollution evaluation, without constructing a multi-scale biological damage system covering molecular biomarkers to community integrity [27]. Most previous studies separate pollutant migration simulation and ecotoxicological damage assessment into independent research modules, while this study organically couples hydrodynamic models, rapid graph theory prediction tools, and multi-level biological indicators, and constructs a three-dimensional economic loss accounting system integrating service loss, restoration cost, and recovery period loss, which reflects the methodological defects of decentralized evaluation in existing mining pollution case studies.

Existing methods have alleviated some bottlenecks in heavy metal pollution prediction, damage assessment, and remediation technologies. However, they still have significant limitations from a systematic perspective. First, the problems of model complexity and high data requirements have not been eliminated. Second, although ecological damage assessment has evolved from reliance on a single indicator toward multidimensional indicator systems, limitations remain in indicator selection, weighting, and integration, making it difficult to comprehensively capture the cumulative, synergistic, and spatiotemporal effects of heavy metal pollution [28,29]. Finally, the closed-loop mechanism from data to decision-making is not yet perfect, and it is difficult to realize the rapid process of monitoring-warning–response–repair.

Against this backdrop, this study takes the Cr(VI) and As composite pollution caused by a typical tailings dam breach accident as the research object, aiming to clarify the migration and transformation laws of these two characteristic pollutants in groundwater–surface water systems, verify the prediction accuracy, computational efficiency, and applicable scope of different models, quantify their multi-scale toxic effects on aquatic organisms, and calculate the full-cycle ecological and economic losses caused by the pollution accident. This study will bridge existing research gaps through a multi-model coupling and three-dimensional assessment framework, thereby providing scientific support for emergency response and ecological compensation of sudden Cr(VI) and As pollution accidents. It should be noted that the field monitoring data used in this validation case were collected under a technical service contract associated with the national project stated in the Funding section. Pursuant to the confidentiality and data-rights clauses of this contract, the contracting party retains ownership and copyright of the primary monitoring data, and the authors are not permitted to disclose information that could identify the research object, including its precise geographic location, the name of the involved enterprise, and the unprocessed raw dataset.

Although the full-cycle ecological damage assessment framework constructed in this study realizes integrated quantitative evaluation of sudden water pollution, it has clear applicable boundary constraints, which are clarified as follows. Geographically, the model is suitable for plain river networks with shallow aquifers and gentle terrain, and cannot be directly applied to steep mountain torrent watersheds or karst fissure groundwater systems without parameter recalibration. In terms of pollutants, the field verification of this framework is based on measured Cr(VI) and arsenic data; Cd- and PAH-related formulas and dose–response curves in the manuscript only act as general template modules of the system and cannot be used as formal damage calculation data without field measured matching monitoring data. In terms of data support, the coupled Soil and Water Assessment Tool (SWAT)-graph theory prediction model requires continuous multi-media monitoring data within 1–6 months after the pollution accident, and cannot output reliable simulation results under sparse and discontinuous sampling datasets [30,31]. In terms of restoration engineering, the cost calculation module is only applicable to conventional ISCR and PRB treatment technologies, and complex combined remediation projects need additional economic parameter correction. In addition, the model is not suitable for pollution incidents with cyanide, ultra-acid mine drainage (pH < 3) as the main pollutants.

Compared with previous scattered evaluation schemes that separate pollutant simulation, biological toxicity testing, and economic loss accounting, this study realizes targeted integrated application of multiple mature, widely recognized evaluation tools rather than proposing fundamentally new methodologies. Three application-oriented improvements are achieved by assembling existing classical models and indicators: first, we couple well-established physical advection–diffusion simulation and graph theory rapid prediction algorithms to balance calculation precision and emergency computing speed for sudden pollution events; second, we combine widely reported molecular, individual, and community biological biomarkers into a continuous multi-layer damage identification chain suitable for mining pollution monitoring data; and third, we integrate three mature economic accounting modules (ecosystem service impairment, restoration expenditure, post-event recovery loss) to complete full-cycle quantitative loss estimation for the selected accident. This work builds an assessment workflow adaptable to sudden water pollution scenarios with similar hydrological and pollution characteristics, and adopts a tailings dam leakage case featuring Cr(VI) and As co-contamination as a practical demonstration case to test the operability of the integrated system. It should be clearly stated that this paper does not contain self-derived mathematical equations, independently developed calculation codes, or extra external case verification datasets. All sub-methods adopted are classic approaches from published studies, and the core advancement of this manuscript only lies in the systematic combination of these discrete tools and their targeted deployment in a representative plain river network tailings pollution incident.

Targeting the unique Cr(VI)–As composite pollution characteristics of plain river network tailings breach sites, this paper assembles a full-cycle assessment workflow with clear applicable boundary constraints and validates its practical performance using multi-media field monitoring data collected from this representative mining pollution case, making up the deficiency of separated evaluation logic in previous tailings disaster research. This study does not propose fundamentally new mathematical formulas, self-programmed calculation codes, or independently validated cross-case models. All pollutant migration, biological toxicity, and economic accounting sub-methods adopted in this study are mature tools derived from peer-reviewed existing studies. The advancement of this study lies only in the organic integration of these discrete, separately used modules into a coherent full-cycle closed-loop assessment workflow, and the targeted application of this integrated framework to the plain river network tailings dam breach case with Cr(VI)–As co-contamination for systematic practical verification.

## 2. Research Cases and Research Methods

### 2.1. Regional Background and Monitoring Plan

#### 2.1.1. Research Object Layout and Spatial Division

The study area is located in an industrial park in the middle and lower reaches of the Yangtze River (geographical coordinates: 118°42′–118°48′ E, 31°15′–31°21′ N), with a total area of 12.6 km^2^. To systematically evaluate the spatio-temporal ecological impacts and recovery dynamics, the study area was strictly partitioned into four distinct zones/phases: (1) Upstream Control Site: Located 5 km upstream of the leakage point (S0), representing the absolute, unpolluted ecological baseline. (2) Core Pollution Zone: Located 0–5 km downstream, representing the area directly and heavily impacted by the tailings slurry during the acute phase. (3) Buffer Pollution Zone: Located 5–15 km downstream, representing the moderately impacted area via diluted transport during the acute phase. (4) Far-Downstream Baseline Zone: Located 15–18 km downstream (B3/S7). Due to long-distance hydrodynamic dilution and river self-purification, the pollutant concentrations here had already attenuated back to background levels during the acute phase, showing no significant difference (*p* > 0.05) from the Upstream Control Site. Consequently, data from the Upstream Control and Far-Downstream Baseline sites were pooled into a unified “Baseline/Control Group” for subsequent comparative analyses. Additionally, the “Temporal Recovery Phase” was defined as the ecological status of the previously impacted downstream reaches (0–15 km) evaluated 6 months after the leakage incident.

The research objects include three interconnected components: (1) the surface water system, consisting of an 18 km main river and its 5 km tributary; (2) the shallow groundwater system within a 2 km radius around the tailings pond, covering the entire sandy aquifer; and (3) the aquatic biological community, including planktonic algae, benthic macroinvertebrates, and fish in the target river. The spatial distribution of the study area and functional zones is shown in Figure S1 in Supplementary Materials Section S1.

#### 2.1.2. Regional Hydrogeological Characteristics

The core pollution event is the explosive heavy metal pollution triggered by the dam failure of the tailing pond of a mining company, with an initial leakage concentration of 85.6 mg/L for Cr(VI) and 102.2 mg/L for total As. These two characteristic pollutants converge into the downstream river through surface runoff (accounting for about 30% of the total leakage) and pollute the shallow groundwater through infiltration and leaching (accounting for about 70% of the total leakage). The hydrological characteristics of the area are as follows: the river is 18 km long, with a flow rate of 0.6 m/s during the level water period, and a tributary joins the river 5 km downstream; the type of groundwater is the pore water of the Quaternary system, with an aquifer thickness of 10–15 m and a permeability coefficient of 18 m/d.

In this study, a three-dimensional multi-media environmental monitoring network covering surface water, groundwater, and aquatic organisms was constructed, following the technical specifications of Surface Water Environmental Quality Standards (GB 3838-2002) [32] and Technical Specifications for Groundwater Environmental Monitoring (HJ/T 164-2004) [33].

#### 2.1.3. Monitoring Point Layout

The monitoring network was designed based on three core principles: (1) covering all functional zones of the study area; (2) reflecting the spatial variation characteristics of pollutant diffusion; and (3) setting up sufficient background and control points for comparative analysis. The spatial distribution map is shown in Figure S1.

- Surface water monitoring: Eight monitoring sections were set up along the 18 km target river, including 1 upstream control section (S0, 5 km above the leakage point) and 7 pollution monitoring sections (S1–S7, located at 1 km, 3 km, 5 km, 8 km, 12 km, 15 km, and 18 km downstream of the leakage point). Each section was divided into three sampling points (left bank, middle channel, right bank) to ensure spatial representativeness.
- Groundwater monitoring: A total of 12 monitoring wells (W1–W12) were arranged within a 2 km radius around the tailings pond, with well depths ranging from 6 m to 15 m (covering the entire sandy aquifer). The wells were distributed along the groundwater flow direction (northwest to southeast) at intervals of 200–500 m, including 3 background wells (W1–W3) upstream of the pollution source, 6 pollution monitoring wells (W4–W9) in the core pollution zone, and 3 diffusion monitoring wells (W10–W12) in the buffer zone.
- Aquatic organism monitoring: Three key monitoring sections were selected corresponding to the functional zones: B1 (core polluted area, 1–3 km downstream), B2 (middle buffer zone, 5–8 km downstream), and B3 (far-downstream baseline zone, 15 km downstream, representing the spatial attenuation endpoint). At each section, samples were collected from three representative habitats (shallow water area, deep water area, and sediment–water interface).

#### 2.1.4. Water Quality Analytical Methods and QA/QC

Water samples collected from the surface-water and groundwater monitoring network were analyzed for Cr(VI), total As, As(III), and As(V). Standard analytical procedures commonly used for heavy-metal monitoring and arsenic speciation analysis were adopted to support the construction of the case-study dataset. To ensure methodological transparency, analytical conditions, calibration information, detection limits, quality control procedures, and arsenic speciation preservation protocols are summarized in Supplementary Materials Section S2 and Table S1.

Because the primary objective of this study is the demonstration and validation of an integrated ecological damage assessment framework rather than analytical method development, detailed instrumental descriptions are provided in the Supplementary Materials.

#### 2.1.5. Sampling Methodology and Field Quality Control

It should be clarified that the surface-water and groundwater sampling described in this section was conducted by the project monitoring team under the technical service contract. In contrast, the aquatic organism sampling protocols and biological indicator determinations described in Section Standardized Sampling Protocols, Section 2.4, and Section 2.5 were performed by the post-accident emergency monitoring teams and their contracted laboratories organized by the local ecological and environmental authority. The authors obtained the resulting biological data from the public emergency monitoring records and did not directly collect, sacrifice, dissect, or process any fish or other aquatic organisms, consistent with the Institutional Review Board Statement.

##### Sampling Frequency

- Acute phase (within 1 month after the accident): Surface water and groundwater samples were collected once daily; aquatic organism samples were collected every 3 days.
- Stable phase (1–6 months after the accident): Sampling frequency was adjusted to once every 3 days for water samples and once every 2 weeks for biological samples.

##### Standardized Sampling Protocols

Surface water sampling: Water samples were collected at 0.5 m below the water surface using a stainless-steel water sampler. At each section, three subsamples were collected from the left bank, middle channel, and right bank, and mixed into a composite sample. For dissolved heavy metal analysis, 1 L of water sample was filtered through a 0.45 μm cellulose acetate membrane on-site, acidified to pH < 2 with ultra-pure HNO_3_, and stored in pre-acid-washed (10% HNO_3_ for 24 h) polyethylene bottles at 4 °C in the dark. For total heavy metal analysis, unfiltered samples were collected and acidified in the same way.

Groundwater sampling: Before sampling, the monitoring wells were purged with 3–5 well volumes of water using a submersible pump until the water temperature, pH, and DO stabilized (variation < 5% within 5 min). Water samples were collected at the wellhead using a polyethylene bailer, and the same filtration and preservation methods as the surface water samples were applied. Water level and hydraulic gradient were measured on-site using a pressure transducer.

Aquatic organism sampling (per post-accident emergency monitoring records):

- Planktonic algae: 1 L of water sample was collected at 0.5 m depth, fixed with 1% Lugol’s iodine solution on-site, and concentrated to 50 mL by sedimentation for 48 h in the laboratory.
- Benthic macroinvertebrates: Three replicate samples were collected using a Peterson grab sampler (0.04 m^2^) at each section. The samples were sieved through a 0.5 mm mesh, sorted on-site, and preserved in 10% neutral buffered formalin solution.
- Fish (*Carassius auratus*): A total of 10–15 healthy adult individuals (body length 15–20 cm, body weight 100–150 g) were collected using gill nets at each section. The fish were dissected on-site within 2 h of collection, and gill and liver tissues were removed, rinsed with ice-cold physiological saline, frozen in liquid nitrogen, and stored at −80 °C until analysis.

##### Field Quality Control

At each monitoring point, 3 parallel samples were collected for all indicators. Field blanks (ultra-pure water carried to the sampling site and processed identically to samples) and transport blanks were included in each batch of 20 samples to assess contamination during sampling and transport. The relative deviation between parallel samples was controlled within 10%.

To provide a concise and intuitive overview of the entire monitoring scheme, the core parameters of the multi-media environmental monitoring program are summarized in Table 1.

#### 2.1.6. Data Classification and Sources

To ensure full transparency regarding the provenance and status of all data used in this study, the case-study data are systematically classified into five categories, as summarized in Table 2: (i) field-monitoring data collected through the multi-media monitoring network under the technical service contract; (ii) public emergency records provided by local environmental and hydrological authorities; (iii) parameters derived from peer-reviewed literature, public databases, and national standards; (iv) template/reference data embedded in the assessment framework as universal modules, which are not case-specific results; and (v) model-generated results produced by the prediction and assessment models described in Section 2.7. It should be emphasized that the template/reference data in category (iv)—including the Cd–metallothionein dose–response relationship, the PAH–CYP1A module, and the PAH-oriented PRB cost module—are included solely to demonstrate the generality and completeness of the framework and do not constitute measured or simulated results for this Cr(VI)/As case.

### 2.2. Core Research Objective

This study adopts a “multi-model coupling prediction + multi-level biological monitoring + three-dimensional comprehensive assessment” integrated research framework. Specifically: (1) the advection–diffusion equation is used to simulate the three-dimensional migration of heavy metals in groundwater aquifers; (2) the SWAT model is applied to simulate the dynamic diffusion of pollutants in surface water systems under complex hydrological conditions; (3) a graph theory-based rapid prediction model is constructed to meet the real-time response requirements of environmental emergencies; (4) a four-level biological damage indicator system covering molecule–individual–group-ecosystem is established to quantify the ecological damage degree; and (5) a three-dimensional assessment framework of “ecological service damage-restoration cost-recovery period loss” is used to calculate the total economic loss of pollution. The core objective is to clarify the migration and transformation laws of Cr(VI) and As in groundwater–surface water systems, verify the accuracy of different prediction models, and provide scientific support for emergency response and ecological compensation of sudden heavy metal pollution.

### 2.3. Water Body Environmental Data

The water body environmental dataset constructed in this study covers three types of indicators comprising surface water, groundwater, and key hydrological parameters. The water body environmental dataset includes surface water/groundwater heavy metal concentrations (Cr(VI), As), conventional water quality parameters (pH, DO, TSS, TN, TP), and hydrological parameters (water level, flow velocity, flow rate). The concentrations of Cr(VI) and As in water samples were determined by ion chromatography (IC) and Atomic Absorption Spectrometry (AAS), respectively. To maintain the conciseness of the main text, the exhaustive technical configurations—including analytical wavelengths, specific columns, mobile phases, speciation preservation protocols, and quality control frequencies—are comprehensively compiled in Table S2 of the Supplementary Materials.

For arsenic speciation analysis, high-performance liquid chromatography coupled with atomic fluorescence spectrometry (HPLC-AFS; AFS-9700, Beijing Haiguang Instrument Co., Ltd., Beijing, China) was used. Chromatographic separation was achieved using a Hamilton PRP-X100 anion-exchange column (250 mm × 4.1 mm, 10 μm) with a mobile phase of 15 mmol/L ammonium dihydrogen phosphate (pH 6.0) at a flow rate of 1.0 mL/min. The injection volume was 100 μL, and As(III) and As(V) were completely separated within 8 min with no significant peak interference. Detailed instrumental parameters are provided in Supplementary Materials Section S2.

Strict quality control measures were implemented throughout the analysis process:

- For total Cr and As analysis: 10% of the samples were analyzed in duplicate (relative deviation < 10%), procedural blanks were included in each batch, and national standard reference material GBW08611 (arsenic single-element solution, National Institute of Metrology, Beijing, China) was used for calibration, with recovery rates ranging from 92% to 108%.
- For arsenic speciation analysis: National standard reference material GBW08611 (arsenic single-element solution, 1000 μg/mL in 1% HNO_3_, National Institute of Metrology, Beijing, China) was used for method validation. The recovery rates for As(III) and As(V) were 88–112% and 85–115%, respectively, with intra-batch relative standard deviations < 5% and inter-batch relative standard deviations < 8%. No detectable arsenic species were found in procedural blanks.

Hydrological parameters were obtained from the local hydrological monitoring station, with daily monitoring records from 2008 to 2019. Detailed quality control data and complete hydrological time series are provided in Supplementary Materials Section S2.

### 2.4. Data of Biological Communities

Biological community data for three indicator groups—planktonic algae (*Microcystis aeruginosa*), benthic macroinvertebrates (*Limnodrilus hoffmeisteri*), and fish (*Carassius auratus*)—were obtained from the post-accident public emergency monitoring records provided by the local ecological and environmental authority. According to these records, algal samples were fixed with Lugol’s iodine solution on-site and identified and counted under an optical microscope (Olympus BX53, Olympus Corporation, Tokyo, Japan); benthic organisms were sorted from sediment samples, preserved in 10% formalin, and identified to the lowest possible taxonomic level; and fish gill and liver tissues were collected during emergency monitoring and stored at –80 °C for subsequent biomarker analysis. The authors did not directly perform any fish collection, dissection, or tissue processing.

Community structure was evaluated using the Shannon–Weiner diversity index (H′) and Pielou evenness index (J), calculated based on species abundance data. Three parallel samples were analyzed at each monitoring point to reduce random error. Detailed taxonomic identification results and community composition data are provided in Supplementary Materials Section S3.

### 2.5. Determination of Biological Indicators

Metallothionein (MT) concentrations in fish gill tissues were determined by the emergency monitoring laboratories using a commercial fish MT ELISA kit (Jiangsu Meimian Industrial Co., Ltd., MM-0523O1, Yancheng, Jiangsu, China) following the manufacturer’s protocol, with minor modifications, as described by Hertika et al. (2023) [35]. Briefly, gill tissues were homogenized in ice-cold phosphate-buffered saline (PBS, pH 7.4) at a ratio of 1:9 (*w*/*v*), centrifuged at 12,000× *g* for 15 min at 4 °C, and the supernatant was collected for analysis. The absorbance was measured at 450 nm using a microplate reader (Tecan Infinite M200 Pro, Tecan Group Ltd., Männedorf, Switzerland), and MT concentrations were calculated against a standard curve (0–100 ng/mL). The resulting MT data for the Cr(VI)/As case were obtained from these emergency monitoring records. The Cd–MT dose–response parameters used as the framework’s reference template (Section 3.3.6) were compiled from established literature [35] and were not measured in this study.

Oxidative stress indicators, including superoxide dismutase (SOD) activity, catalase (CAT) activity, and malondialdehyde (MDA) content in fish liver tissues, were determined by the emergency monitoring laboratories using commercial assay kits (Nanjing Jiancheng Bioengineering Institute, A001-1, A007-1, A003-1, Nanjing, China). SOD activity was measured by the xanthine oxidase method [36], which quantifies the inhibition rate of nitroblue tetrazolium reduction [37]. CAT activity was determined by the ammonium molybdate method, which measures the decomposition rate of hydrogen peroxide at 405 nm [38]. MDA content was analyzed by the thiobarbituric acid (TBA) method, which reflects the degree of lipid peroxidation in biological tissues [39].

The core principle of each method is as follows: SOD activity was measured by the xanthine oxidase method, CAT activity by the ammonium molybdate method, and MDA content by the thiobarbituric acid (TBA) method.

Detailed operational steps, reagent formulations, and quality control parameters for all biological indicator assays are provided in Supplementary Materials Section S4. The specific determination results of oxidative stress indicators are presented in Supplementary Materials Section S12.

### 2.6. Analytical Methodology, Equipment, and Laboratory Information

#### 2.6.1. Analytical Methodology

All analytical methods were performed in accordance with national standard methods and peer-reviewed published literature, with strict internal quality control measures implemented throughout the process.

Conventional water quality parameters: pH, dissolved oxygen (DO), and temperature were measured on-site using a portable multi-parameter water quality analyzer. Total suspended solids (TSS) were determined by the gravimetric method (GB 11901-1989) [40]. Total nitrogen (TN) and total phosphorus (TP) were analyzed by alkaline potassium persulfate digestion-ultraviolet spectrophotometry (GB 11894-1989) [41] and ammonium molybdate spectrophotometry (GB 11893-1989) [42], respectively.

Heavy metal analysis: Total Cr and As concentrations were determined by atomic absorption spectrophotometry (AAS) (GB 7475-1987 [43], GB 7485-1987 [44]). Dissolved Cr(VI) was analyzed by ion chromatography (IC) using the diphenylcarbazide colorimetric method (HJ 779-2015 [45]). Arsenic speciation (As(III)/As(V)) was determined by high-performance liquid chromatography coupled with atomic fluorescence spectrometry (HPLC-AFS). The detailed instrumental parameters and quality control data are provided in Supplementary Materials Section S2.

Biological indicator analysis: Metallothionein (MT) concentrations in fish gill tissues were determined by the emergency monitoring laboratories using enzyme-linked immunosorbent assay (ELISA). Superoxide dismutase (SOD) activity, catalase (CAT) activity, and malondialdehyde (MDA) content in fish liver tissues were measured by spectrophotometry. The detailed operation steps and reagent formulations are provided in Supplementary Materials Section S4.

#### 2.6.2. Complete List of Research Equipment

All equipment used in the study is listed in Table 3, classified by application scenario:

#### 2.6.3. Laboratory Information

All water sample analyses were conducted in the State Key Laboratory of Urban Water Resource and Environment, Harbin Institute of Technology. The laboratory holds China Metrology Accreditation (CMA) certification and operates in strict accordance with the ISO/IEC 17025 [34] quality management system. All analysts are professionally trained and certified, and all equipment is calibrated annually by national metrological institutions.

### 2.7. Core Mathematical Models and Theoretical Basis

#### 2.7.1. Groundwater Pollutant Migration Model (Advection–Diffusion Equation)

The migration of heavy metals in porous media follows the law of mass conservation, and the advection–diffusion equation is used to describe the comprehensive process of advection, dispersion, adsorption, degradation, and source-sink exchange [46]. The expression of the advection–diffusion equation model is as shown in Formula (1).

(1) $$\begin{array}{c} \frac{\partial (\theta C)}{\partial t}=\nabla \cdot \left(\theta D_{h}\nabla C\right)-\nabla \cdot \left(\theta vC\right)-\lambda \theta C+Q_{s}+\sum R_{kin} \end{array}$$

where: θ is volumetric water content of the aquifer (dimensionless); *C* is concentration of pollutants in groundwater (mg/L); t is time (s); $D_{h}$ is hydrodynamic dispersion coefficient tensor (m^2^/s); *v* is seepage velocity vector (m/s); λ is microbial degradation rate constant (s^−1^); $Q_{s}$ is Non-Aqueous Phase Liquid (NAPL) phase dissolution flux (mg/(L·s)); and $\sum R_{kin}$ is net rate of surface complexation adsorption (mg/(L·s)).

The detailed parameterization of each sub-process and model calibration method are provided in Supplementary Materials Section S5.

Model Features and Applicability:

This model is a physically based numerical model that can accurately describe the three-dimensional migration process of pollutants in heterogeneous porous media. It considers multiple physical and chemical processes such as advection, dispersion, adsorption, degradation, and phase exchange, and is suitable for long-term prediction of groundwater pollution in homogeneous or weakly heterogeneous aquifers.

Model Limitations and Quality Control:

(1) This model does not consider the colloid-facilitated transport of heavy metals, which may lead to underestimation of the long-distance migration potential of pollutants.
(2) The model results are highly sensitive to the hydrodynamic dispersion coefficient (Dh).

To reduce the impact of these limitations, we used the SUFI-2 algorithm for parameter calibration and uncertainty analysis, and the 95% prediction uncertainty interval contained 89% of the observed data, ensuring the reliability of the simulation results.

#### 2.7.2. Surface Water Pollutant Migration Model (SWAT Model)

The SWAT model is a semi-distributed hydrological model widely used in watershed water quality simulation [47]. For heavy metal transport simulation, the model couples the hydrological cycle module, sediment transport module, and pollutant migration module. The core Formula (2) for dissolved heavy metal transport is as follows:

(2) $$\begin{array}{c} C_{diss}=C_{0}\cdot e^{-k\cdot t}+\frac{Q_{in}\cdot C_{in}}{Q_{out}}\cdot \left(1-e^{-k\cdot t}\right) \end{array}$$

where: $C_{diss}$ is dissolved heavy metal concentration at time t (mg/L); $C_{0}$ is initial concentration (mg/L); *k* is comprehensive attenuation coefficient (d^−1^); $Q_{in}$/$Q_{out}$ is inflow/outflow of the river section (m^3^/s); and $C_{in}$ is inflow pollutant concentration (mg/L).

The adsorption of heavy metals by suspended solids is described by the Freundlich isotherm equation (originally only in Supplementary Materials Section S7):

(3) $$\begin{array}{c} S=K_{f}\cdot C^{n} \end{array}$$

where: *S* is adsorption capacity of suspended solids (mg/kg); $K_{f}$ is Freundlich adsorption coefficient (L/kg); and *n* is empirical constant (dimensionless).

The detailed model construction and calibration process are provided in Supplementary Materials Section S7.

Model Features and Applicability:

The SWAT model is a semi-distributed hydrological model that can simulate the hydrological cycle and pollutant transport process at the watershed scale. It has strong adaptability to complex land use and soil conditions, and is suitable for long-term simulation of non-point source pollution and point source pollution in large and medium-sized watersheds.

Model Limitations and Quality Control:

(1) The model has relatively low simulation accuracy for extreme rainstorm events (NSE = 0.62) due to the limited temporal resolution of meteorological data (daily scale).
(2) The model simplifies the mixing process at tributary confluences, which may lead to certain errors in the simulation of sudden point source pollution.

To reduce the impact of these limitations, we set a 3-year warm-up period (2017–2019) to eliminate the influence of initial conditions, and used high-resolution DEM data (30 m × 30 m) to improve the accuracy of hydrological simulation.

#### 2.7.3. Graph Theory-Based Rapid Prediction Model

The river system is abstracted as a directed graph G = (V, E), and the pollutant diffusion process is transformed into a directed propagation problem on the graph [30]. The core matrix equation (originally only in Supplementary Materials Section S8) is:

(4) $$\begin{array}{c} \frac{dC}{dt}=\left(A⊗W\right)C-\left(D_{a}⊗W\right)C-kIC \end{array}$$

where: *C*(t) is concentration vector of each river section (mg/L); *A* is adjacency matrix of the river system (dimensionless); *W* is weight matrix containing flow velocity and river length (s^−1^); $D_{a}$ is diagonal matrix of node out-degree (dimensionless); *I* is identity matrix (dimensionless); and *k* is natural degradation coefficient of pollutants (s^−1^).

The detailed derivation process and model validation results are provided in Supplementary Materials Section S8.

Model Features and Applicability:

This model abstracts the river system into a directed graph structure, converting the traditional partial differential equation solving problem into a graph traversal problem. It has extremely high computational efficiency (calculation time reduced from hours to minutes) and low data requirements, and is particularly suitable for rapid prediction and early warning of sudden point source pollution in emergency response scenarios.

Model Limitations and Quality Control:

(1) The model simplifies the flow mixing process at tributary confluences, resulting in a maximum relative error of 18.2% at confluence sections.
(2) This model is only applicable to plain river networks with relatively uniform flow velocity and no large hydraulic structures (dams, sluices), and is not suitable for mountainous rivers with significant elevation changes.

In this study, the graph theory model is only used for rapid emergency prediction in the first 72 h after the accident. For accurate simulation of medium- and long-term pollution processes, we still use the SWAT model with higher precision.

#### 2.7.4. Comprehensive Ecological Damage Assessment Model

The total comprehensive economic loss of pollution is calculated by the three-dimensional assessment framework:

(5) $$\begin{array}{c} C_{total}=D_{t}+C_{repair}+L_{rec} \end{array}$$

where: $D_{t}$ is cumulative discounted value of ecosystem service function damage (CNY); $C_{repair}$ is total cost of environmental restoration (CNY); and $L_{rec}$ is loss of ecosystem function recovery period (CNY).

The Ecological Damage Index (EDI) is constructed to quantitatively characterize the damage degree:

(6) $$\begin{array}{c} EDI=w_{s}\cdot \sum \Delta E_{fish,i}+w_{r}\cdot \sum E_{j} \end{array}$$

where: $w_{s}/w_{r}$ is weight of supply services/regulating services (dimensionless); $\Delta E_{fish,i}$ is economic loss of the *i*-th type of secondary consumers (10,000 CNY); and $E_{j}$ is loss amount of the *j*-th type of regulating service (10,000 CNY).

The detailed assessment method and parameter values are provided in Supplementary Materials Section S16.

### 2.8. Statistical Analysis

All statistical analyses were performed using R software (version 4.3.1). The SUFI-2 algorithm was used for model parameter calibration and uncertainty analysis, with the Nash–Sutcliffe Efficiency (NSE) coefficient as the objective function. The one-factor-at-a-time (OAT) method was used for parameter sensitivity analysis. Generalized Additive Models (GAMs) and Structural Equation Models (SEMs) were used to analyze the relationship between environmental factors and biological damage indicators. The Shapiro–Wilk test was used to test the normality of data distribution, and one-way ANOVA was used to compare differences between groups, with a significance level set at *p* < 0.05. All statistical models in this study are only applied to the analysis of data related to Cr(VI) and As.

### 2.9. Quality Assurance and Quality Control (QA/QC)

Strict quality control measures were implemented throughout the entire research process:

(1) Field sampling: All sampling containers were pre-washed with 10% nitric acid for 24 h. Field blanks and transport blanks were included in each batch of 20 samples, with relative deviations between parallel samples controlled within 10%.
(2) Laboratory analysis: National standard reference materials were used for method calibration, with recovery rates ranging from 85% to 115%. Intra-batch relative standard deviations were controlled below 5%, and inter-batch relative standard deviations below 8%.
(3) Model simulation: A 3-year warm-up period was set for all models to eliminate the influence of initial conditions. The 95% prediction uncertainty interval contained more than 85% of the observed data, ensuring the reliability of simulation results.

## 3. Results and Discussion

The case study focuses on a heavy metal leakage incident caused by the cracking of the dam of a tailings pond of a mining company. The tailings pond is located in the middle and lower reaches of the Yangtze River (hereinafter referred to as the “study area”), containing characteristic heavy metals such as hexavalent chromium (Cr(VI)) and arsenic (As). The partial collapse of the dam of the tailings pond led to the leakage of approximately 1.2 × 10^4^ m^3^ of tailings slurry, causing explosive water pollution.

It should be clarified that the proposed environmental damage assessment framework adopts a modular structure designed in principle to accommodate multiple categories of environmental contaminants and ecological stressors. This study employs a Cr(VI)- and arsenic-related tailings-dam leakage event as a demonstration case; the framework has not been validated for other stressors (including heavy metals, organic pollutants, algal toxins, and emerging contaminants), and any extension to such stressors would require case-specific parameter calibration, biological endpoint selection, and independent validation against field data.

The biological endpoints, ecological indicators, and ecosystem-service valuation methods incorporated into the framework are intentionally designed to be applicable to multiple contaminant classes and environmental stressors. Therefore, examples involving contaminants other than Cr(VI) and arsenic are included solely to illustrate the general applicability of the framework and should not be interpreted as additional target pollutants in the present case study. The classification and sources of all data used in this study are summarized in Table 2.

### 3.1. Prediction of Groundwater Pollutant Diffusion Results

Based on the advection–diffusion equation model described in Section 2.7.1, and using the SUFI-2 algorithm for parameter calibration and uncertainty analysis, this section analyzes the spatio-temporal diffusion characteristics of Cr(VI) and As in groundwater, and verifies the prediction accuracy of the model through on-site monitoring data.

#### 3.1.1. Basic Conditions of the Case (Groundwater Advection–Diffusion Model)

The hydrogeological structure of the study area is divided into three layers from top to bottom. The first layer is a silty clay layer (3 to 5 m thick), with a permeability coefficient K = 1.2 × 10^−7^ cm/s and a volumetric water content θ = 0.32. It is a weakly permeable layer and is prone to pollutant retention. The second layer is a sandy aquifer (10 to 15 m thick), with a permeability coefficient K = 18 m/d and a volumetric water content θ = 0.45. It is the main channel for pollutant migration, with a groundwater level depth of 6.5 m, flowing from northwest to southeast, and a hydraulic gradient ∇h = 0.0025. The last layer is a clay layer (thickness > 20 m), with a permeability coefficient K < 10^−9^ cm/s, which is an impermeable layer and restricts the downward migration of pollutants. For the multi-process coupling in complex hydrogeological structures, a spatio-temporal explicit prediction framework based on unstructured grids is established. The expression of the advection–diffusion equation model is as shown in Formula (1). The detailed analysis can be found in the Supplementary Materials Section S5.

A total of 120 sets of groundwater monitoring data (from 12 monitoring wells over 10 consecutive days) were used for model calibration and validation. The dataset was randomly partitioned into a calibration set (70%, 84 sets) and a validation set (30%, 36 sets) using the stratified sampling method to ensure representative coverage of different time periods and spatial locations. Outliers were identified and removed using the 3σ criterion, resulting in the exclusion of three data points with measurement errors exceeding three standard deviations. All remaining data were normalized to the range [0, 1] to eliminate dimensional effects before model input.

This study employed a pollutant migration model based on the advection–diffusion equation to predict the groundwater pollution diffusion process. The key parameters of the model were calibrated based on-site monitoring data and literature. The specific values are shown in Table S3.

#### 3.1.2. Spatio-Temporal Characteristics of Pollutant Diffusion

Based on the numerical solution of the advection–diffusion equation (using the finite difference method for discretization), the concentration distribution of groundwater pollutants at different times after leakage was predicted.

(1) The results 48 h after the leakage occurred are presented below. The Cr(VI) contamination plume spread horizontally to a distance of 250 m, with the concentration peak dropping to 28.7 mg/L. The As contamination plume diffused more slowly due to adsorption effects, with a horizontal diffusion range of 140 m and a concentration peak of 10.2 mg/L.
(2) The results 7 days after the leakage occurred are presented below. Affected by rainfall leaching (average monthly rainfall in the study area is 120 mm), the groundwater level rose by 0.8 m, the pollution plume of Cr(VI) spread to 560 m horizontally, and the vertical infiltration depth reached 8 m, with the concentration peak dropping to 12.3 mg/L. Pentavalent arsenic (As(V)) in the initial pollution plume was reduced to highly mobile trivalent arsenic (As(III)) due to the decrease in Eh (anaerobic zone formation, Eh = −80 mV), and the diffusion range expanded to 470 m.
(3) The results 30 days after the leakage occurred are presented below. Cr(VI) forms a retention zone in the low-permeability clay layer, with a stable concentration of 0.8 mg/L, the pollution plume of As(III) continues to spread, forming a hotspot area with a concentration of 0.42 mg/L.

Particularly for the groundwater module, the simulation results captured a highly dynamic migration process. The diffusion distance of As in the aquifer expanded from 140 m on Day 1 to 470 m by Day 7. This represents a net migration increase of 330 m, equating to a rapid spatial expansion of approximately 235% over the 7-day monitoring period.

**Figure 1:**
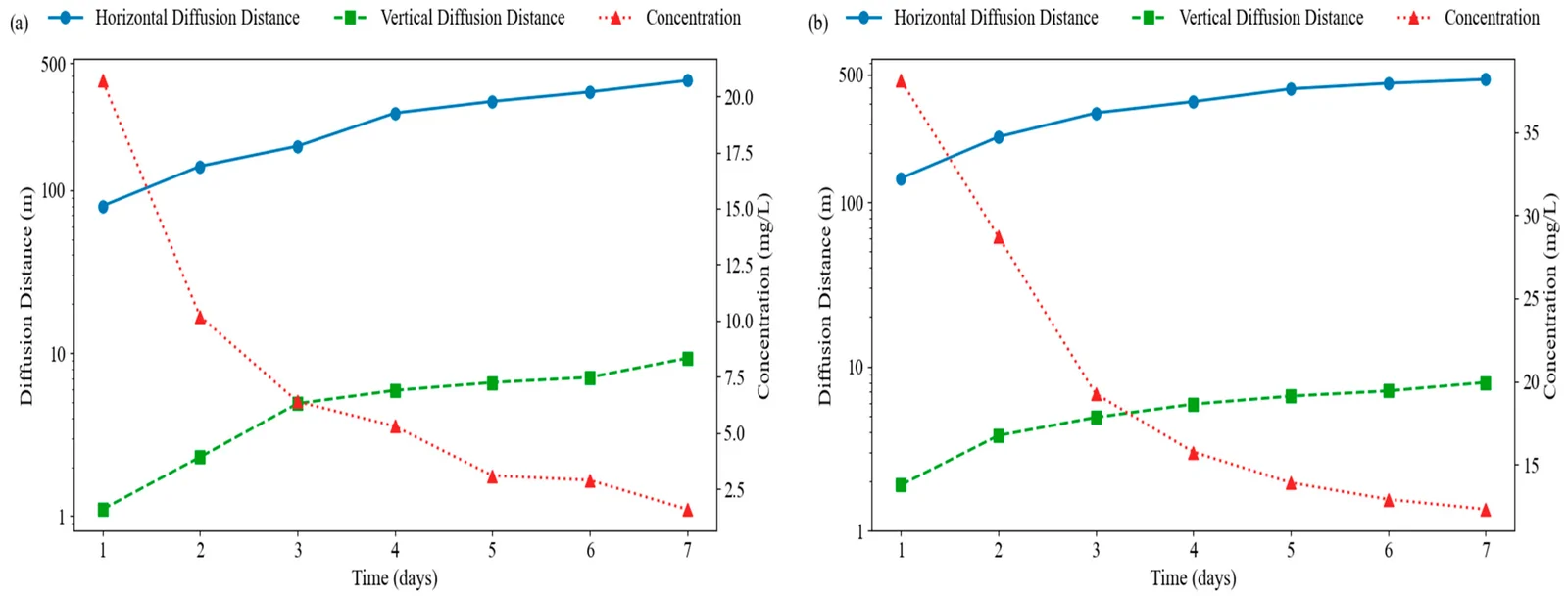
Contamination plume evolution (1–7 days) (**a**) As. (**b**) Cr(VI).

The pollution plume of As (Figure 1a) undergoes adsorption (with a higher K_d_ value) and later transforms into a more mobile form of As(III) in the anaerobic zone. As a result, its morphology becomes more dispersed and shows stronger vertical penetration. The pollution plume of Cr(VI) (Figure 1b) exhibits a significant finger-like expansion along the high-permeability sand layer (K = 18 m/d). The horizontal transport distance (560 m) is much greater than the vertical penetration depth (8 m), as shown in Figure 1.

#### 3.1.3. Parameter Calibration, Validation, and Uncertainty Analysis

The Sequential Uncertainty Fitting version 2 (SUFI-2) algorithm was used for model parameter calibration, with 1000 iterations performed until the objective function (Nash–Sutcliffe Efficiency, NSE) converged. The calibration results showed that the model achieved an NSE of 0.82 for Cr(VI) and 0.76 for As in the calibration set, and 0.78 for Cr(VI) and 0.71 for As in the validation set, meeting the requirements for groundwater pollution prediction accuracy.

Parameter sensitivity analysis was conducted using the one-factor-at-a-time (OAT) method, and the results confirmed that the hydrodynamic dispersion coefficient (Dh) was the most sensitive parameter, contributing 42% to the overall prediction uncertainty. The 95% prediction uncertainty (95PPU) interval contained 89% of the observed data, indicating that the model had good uncertainty characterization ability.

The detailed calibration and uncertainty analysis results are provided in Supplementary Materials Section S5.6. The detailed parameter sensitivity ranking and key impact scenarios are provided in Supplementary Materials Section S6 and Table S5.

### 3.2. Prediction of the Dispersion Results of Surface Water Pollutants

Based on the SWAT model and graph theory model described in Section 2.7.2 and Section 2.7.3, and using the Muskingum routing method for flow simulation, this section analyzes the dynamic diffusion process of Cr(VI) and As in surface water under different hydrological conditions, and compares the performance of the two models.

#### 3.2.1. Case Background and Characteristics of Surface Water Systems (SWAT Model)

After the tailings leakage, approximately 30% of the tailings slurry flowed through surface runoff into a certain river downstream of the study area (hereinafter referred to as the target river), resulting in a combined pollution of point source (the leakage point directly entered the river) and non-point source (rainwater washing the tailings accumulation area).

The SWAT model was used for predicting the pollution diffusion of surface water. The total length of the target river is 18 km, with an average cross-sectional width of 15 m. The average flow velocity during the flood period is 0.6 m/s, and during the dry period it is 0.3 m/s. At a distance of 5 km downstream, a tributary flows into it (the diversion ratio is 65% of the main river). The initial water quality pH = 7.5, dissolved oxygen DO = 8.2 mg/L, and suspended solids concentration TSS = 15 mg/L, which complies with the Class II standard of GB 3838-2002 [32] “Surface Water Environmental Quality Standards”. The riverbed quality is mainly sandy, and the land-use types include cultivated land (42%), forest land (35%), construction land (15%), and water area (8%). The soil type is mainly yellow-brown soil (saturation hydraulic conductivity K_s_ = 1.5 cm/h), and the catchment area A = 0.5 km^2^.

Based on the SWAT model, a hydrological–water quality coupling simulation for the target river basin was constructed. The model input data included spatial data (DEM, land use map, soil map), meteorological data (daily rainfall, temperature, wind speed from 2008 to 2019), and pollution data (time series of point source emissions from tailings leakage and sequence of storm events for surface water pollution).

##### SWAT Model Data Preprocessing and Warm-Up Period

All input data were preprocessed to ensure consistency in spatial resolution (30 m × 30 m) and temporal resolution (daily). The DEM data were obtained from the Shuttle Radar Topography Mission (SRTM), land use data from the 2020 Landsat 8 satellite imagery, and soil data from the Harmonized World Soil Database (HWSD). A 3-year warm-up period (2017–2019) was set to eliminate the influence of initial conditions on model simulation results. The model was calibrated using daily flow data from 2020 to 2021 and validated using data from 2022 to 2023, achieving an NSE of 0.78 for flow simulation. The detailed calibration parameters and sensitivity analysis results are provided in Supplementary Materials Section S7 and Table S6.

At 24 h after the leakage, the high concentration band of Cr(VI) extended along the river for 8 km. Due to the diversion of tributaries, the concentration peak in the main river decreased to 0.35 mg/L. At 48 h after the leakage, the concentration peak further decreased to 0.18 mg/L. At 4.5 h after the rainstorm event (with a 4.5-h lag in the peak rainfall), the surface source Cr(VI) load reached a peak of 1.1 mg/L, causing the Cr(VI) concentration in the 1–5 km section of the target river to generally exceed 0.5 mg/L. At 24 h after the rainstorm, due to sedimentation, the particulate As was enriched in the river bottom sediment, with a concentration of 28.5 mg/kg. If the rainfall increased to 150 mm (a 20-year flood), the runoff rate of the target river increased to 1.2 m/s, the diffusion range of Cr(VI) expanded to 12 km, and the resuspension of the bottom sediment led to secondary pollution of As, with the water body As concentration sharply increasing to 0.3 mg/L. The specific prediction method can be found in Section S7 of the Supplementary Materials. The model validation results are shown in Table 4. It can be seen that the simulated values of Cr(VI) and As are in good agreement with the monitoring values (three sections of the target river, located 1 km, 3 km, and 8 km downstream of the leakage point, respectively), meeting the requirements for water quality prediction accuracy.

#### 3.2.2. Scenario Prediction Results

**Figure 2:**
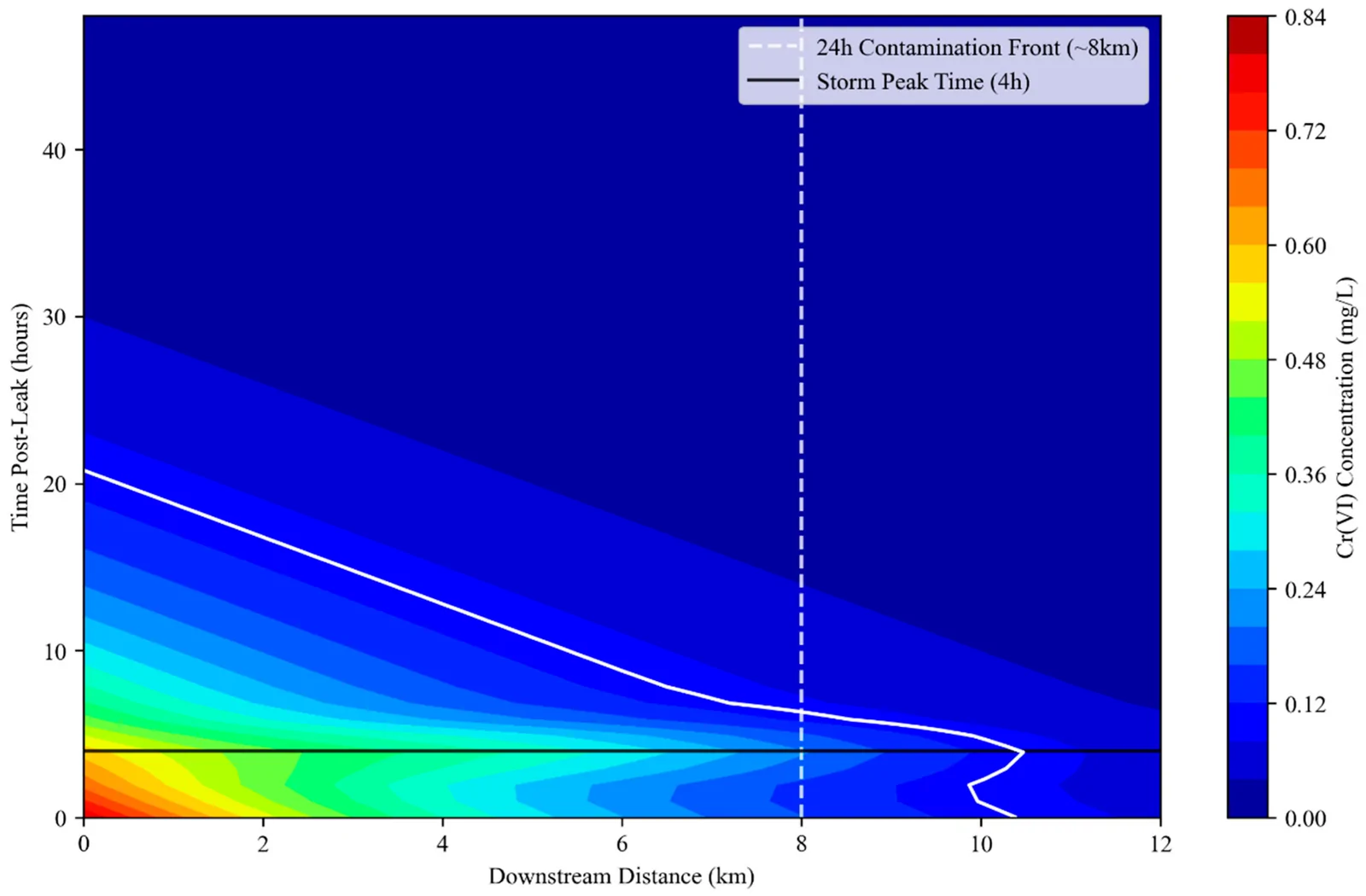
Variation of Cr(VI) concentration with downstream distance and time.

The relationship between Cr(VI) concentration and the distance downstream and time is shown in Figure 2.

During the initial stage of the leakage (0–12 h), a hot pollution core forms in the near-source area, with the concentration rising sharply; subsequently, this high-concentration area behaves like a mobile pollution cloud, with its core moving downstream along with the water flow. At the same time, the intensity gradually decreases due to dilution and diffusion, demonstrating the dynamic process of advection–diffusion coupling. The contour lines (lines of equal concentration) slope downstream along the time axis, clearly indicating the transport speed of the pollution front. At approximately 4 h and 8 km, there is a significant concentration bulge, which closely matches the simulated rainstorm event time, proving that heavy rainfall, by intensifying surface source pollution scouring and sediment resuspension, has a “pulsed” impact effect on the river water quality.

#### 3.2.3. Fast Prediction of Point Source Pollution Based on Graph Theory

The river system is abstracted as a directed graph G=(V,E), achieving the discretization mapping of the continuous river space. The detailed analysis can be found in the Supplementary Materials Section S8.1.

A graph model is constructed and the derivation of the graph model representation is performed from both the physical and mathematical perspectives. Specifically, it is divided into two parts, consisting of the consistency of physical processes and mathematical logical derivation [30]. The detailed analysis can be found in the Supplementary Materials Section S8.2.

The simulation results show that 2 h after the leakage occurred, the front of the Cr(VI) pollution plume reached the 1 km downstream section of the v_10_ section, with a concentration of 12.3 mg/L; 8 h after the leakage, the pollutants migrated to the 5 km downstream section of the v_16_ section, with a concentration of 0.42 mg/L. In terms of inter-model consistency, the relative deviation from the SWAT simulation result (0.38 mg/L) is 10.5%. When validated against the on-site measured concentration (0.39 mg/L) at the same section, the relative error is 7.7%. The calculation time of this model is significantly shortened from the hour level of the SWAT model to the minute level, which significantly improves the assessment efficiency and meets the real-time prediction requirements in environmental emergency response.

##### Model Validation and Applicable Scope

The graph theory model was validated using 50 sets of independent monitoring data from eight river sections (covering both dry and flood seasons), with on-site measured Cr(VI) concentrations used as the reference benchmark. The results showed that the graph theory model achieved an average relative error of 14.2%, a root mean square error (RMSE) of 0.055 mg/L, and a coefficient of determination (R^2^) of 0.87. Stratified statistics indicated that the average relative error was 12.3% for straight main river sections, and rose to 20.1% at the tributary confluence section; the larger error at the confluence was attributed to the simplified treatment of flow mixing in the graph theory model.

For the 72-h emergency response window after the accident (the core application scenario of this model), the average relative error was 13.1%. For high-concentration sections (Cr(VI) > 0.3 mg/L) that are the focus of emergency early warning, the average relative error dropped to 11.6%, showing higher prediction accuracy in the risk-concerned interval. The inter-model comparison with the SWAT model showed an average relative deviation of 10.5%, indicating that the graph theory model maintains a prediction accuracy level close to the mature hydrological water quality model while greatly improving computational efficiency. Detailed validation statistics against field monitoring data are provided in Table S7 of Supplementary Materials Section S8, and the computational efficiency comparison with the SWAT model is summarized in Table S8 of the same section.

It should be noted that this graph theory model is most suitable for plain river networks with relatively uniform flow velocity and no large hydraulic structures (such as dams and sluices). It is not applicable to mountainous rivers with significant elevation changes or complex hydraulic conditions, where the traditional SWAT model remains more appropriate. The above validation results are obtained based on the single-point source sudden pollution scenario in this study, and its generalization performance under other hydrological and pollution scenarios needs to be further verified by more case studies.

### 3.3. Analysis of Damage Indicators for Aquatic Organisms

The biological damage assessment is a core component of the proposed framework, designed to be applicable to multiple types of pollutants. The indicator system covers heavy metals (such as Cd and Cr) and organic pollutants (such as PAHs), with endpoints ranging from molecular/cellular levels to individual/physiological and population/community levels. In this case study, Cr(VI) and As are the primary target pollutants. The benthic organism response to As is shown in Section 3.3.7, and the Cr(VI) bioaccumulation and community-level impacts are presented in Section 3.3.8. The following sections describe the complete indicator system of the framework, including standardized dose–response relationships for representative non-case pollutants (Cd and PAHs), which are retained as universal reference templates to demonstrate the versatility and comprehensiveness of the assessment method; these template data are clearly distinguished from the Cr(VI)/As field results and are not used in any case-specific calculation.

#### 3.3.1. Research Subjects and Monitoring Plans

Taking the target river and its surrounding water bodies as the research object, selected planktonic algae (*Microcystis aeruginosa*), benthic organisms (*Limnodrilus hoffmeisteri*), and fish (carp) as indicator organisms, we monitored the target river and adjacent waters in three replicated groups, including the core pollution group, the baseline/control group (pooling the upstream control and far-downstream baseline sites), and the temporal recovery phase (6 months post-leak). Additionally, we used Generalized Additive Models (GAMs) and Structural Equation Models (SEMs) to analyze the relationship between environmental factors and biological damage. We measured algal chlorophyll a, enzyme activities and community structure, benthic avoidance/adsorption and abundance, and fish biomarkers, development, and biomass. The detailed analysis can be found in the Supplementary Materials Table S9. A hierarchical index system linked molecular or cellular responses with individual toxicity and community impacts. These multi-endpoint diagnostics support ecological risk assessment across different pollution scenarios. The detailed analysis can be found in the Supplementary Materials Section S9.

#### 3.3.2. Correlation Analysis of Aquatic Biological Statistical Information and Environmental Factors

The responses of aquatic organisms to environmental stress exhibit significant spatio-temporal heterogeneity and nonlinear characteristics. The precise assessment of the damage degree requires the establishment, based on the analysis, of the coupling mechanism between biological statistical information and environmental driving factors. This study employed a multi-scale statistical modeling approach to systematically quantify the interaction between aquatic biological indicators and key environmental parameters. It revealed the regulatory pathways and threshold effects of environmental factors on biological damage. The data collection can be found in the Supplementary Materials Section S10.

#### 3.3.3. The Combined Mechanism of Environmental Factors on Biological Damage

**Figure 3:**
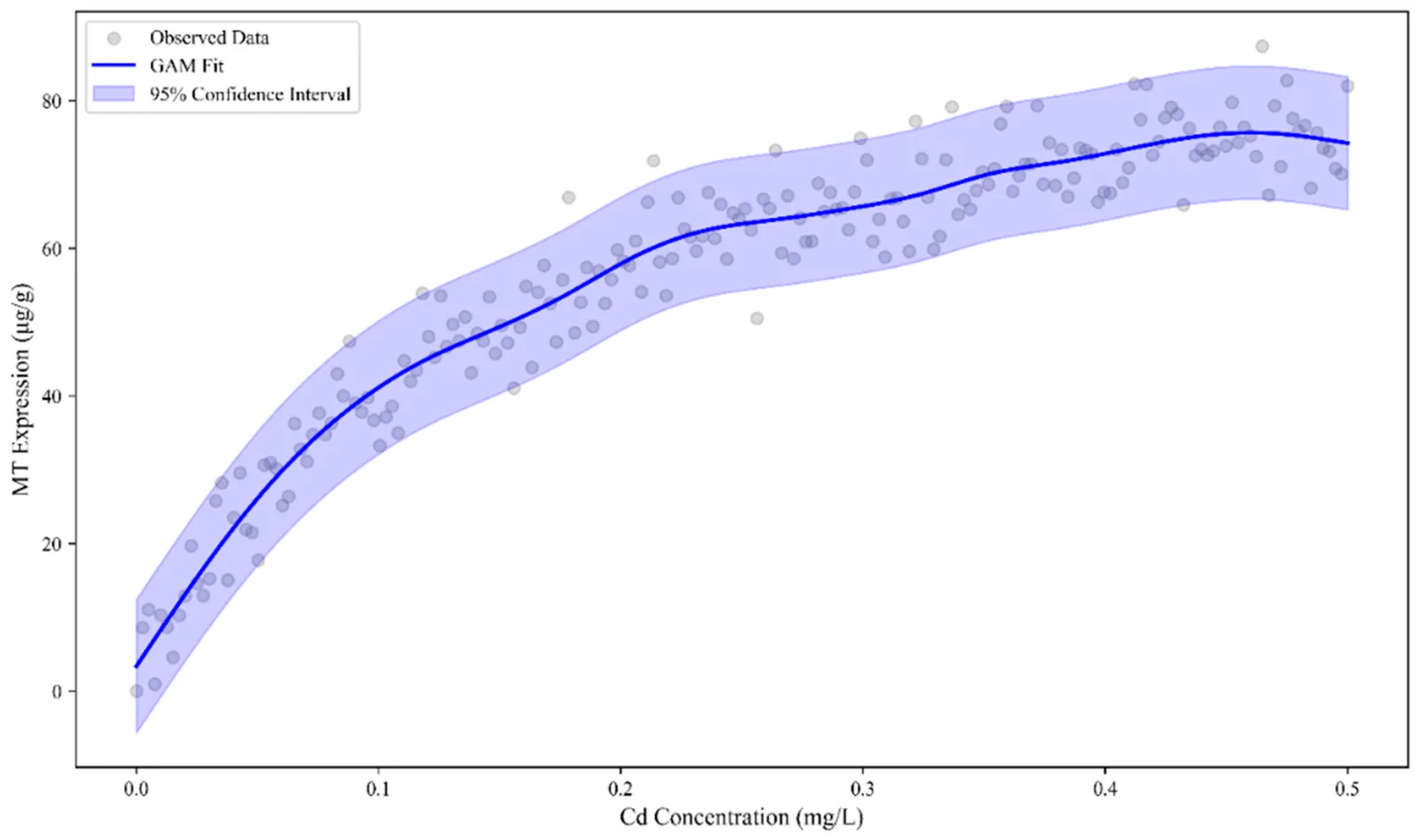
Effect of Cd concentration on MT expression in crucian carp gill tissue.

Through the coupled analysis of the generalized additive model (GAM) and structural equation model (SEM), the combined mechanism of environmental factors on biological damage was revealed. There was a nonlinear relationship between pollutant concentration and biological response. For the heavy metal Cd^2+^ concentration within the range of 0.05–0.2 mg/L, the expression level of metallothionein (MT) in the gill tissue of crucian carp showed exponential growth (R^2^ = 0.92, *p* < 0.001), but when the concentration was greater than 0.3 mg/L, it tended to saturate, which was in line with the Michaelis–Menten kinetic model, as shown in Figure 3.

**Figure 4:**
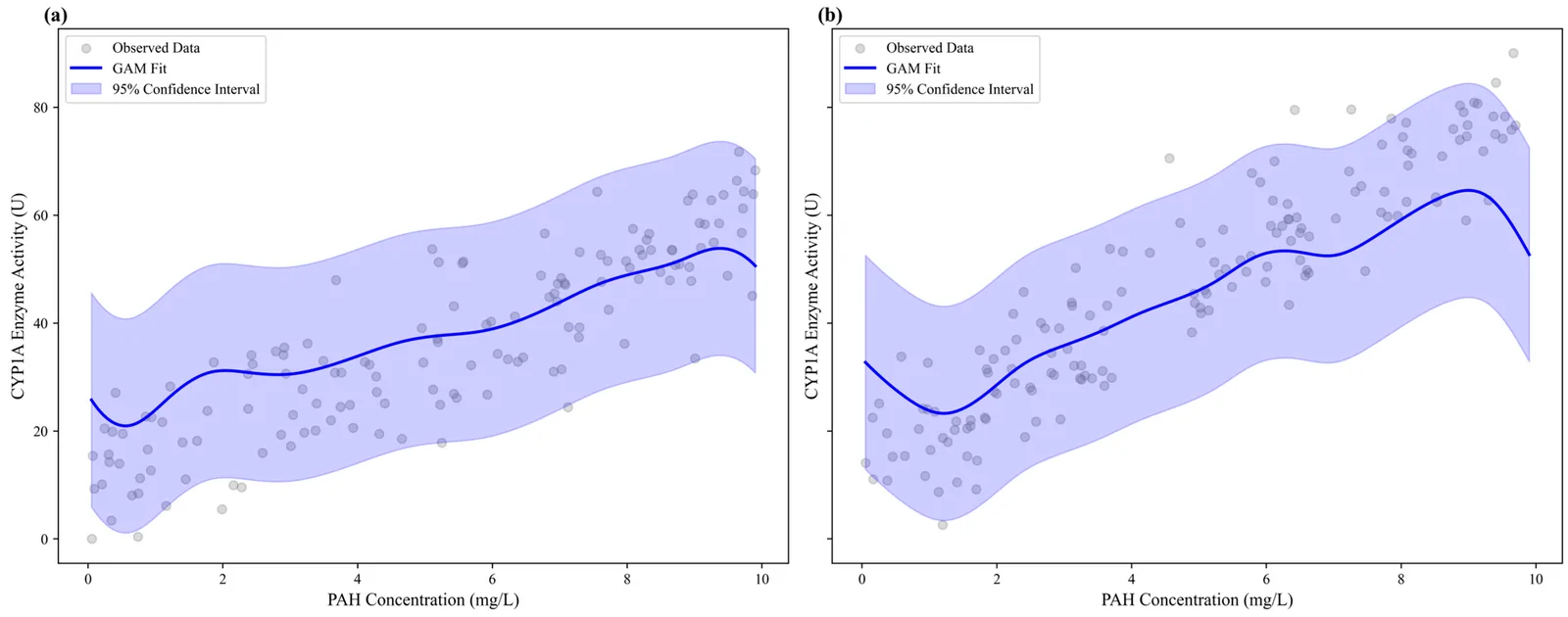
Effect of PAH concentration on algal CYP1A enzyme activity. (**a**) low light intensity (**b**) high light intensity.

The concentration of PAH has a significant impact on the activity of the CYP1A enzyme in algae. Under exposure to polycyclic aromatic hydrocarbons (such as benzo[a]pyrene), the CYP1A enzyme activity of algae was significantly positively correlated with the pollutant concentration (r = 0.78), but its induction effect was modulated by the intensity of light—the increase in enzyme activity under strong light (>1000 μmol·m^−2^·s^−1^) was 40% higher than that in the low light environment, indicating the key influence of photochemical activation on the metabolism of organic toxins. The specific effect expression is shown in Figure 4.

**Figure 5:**
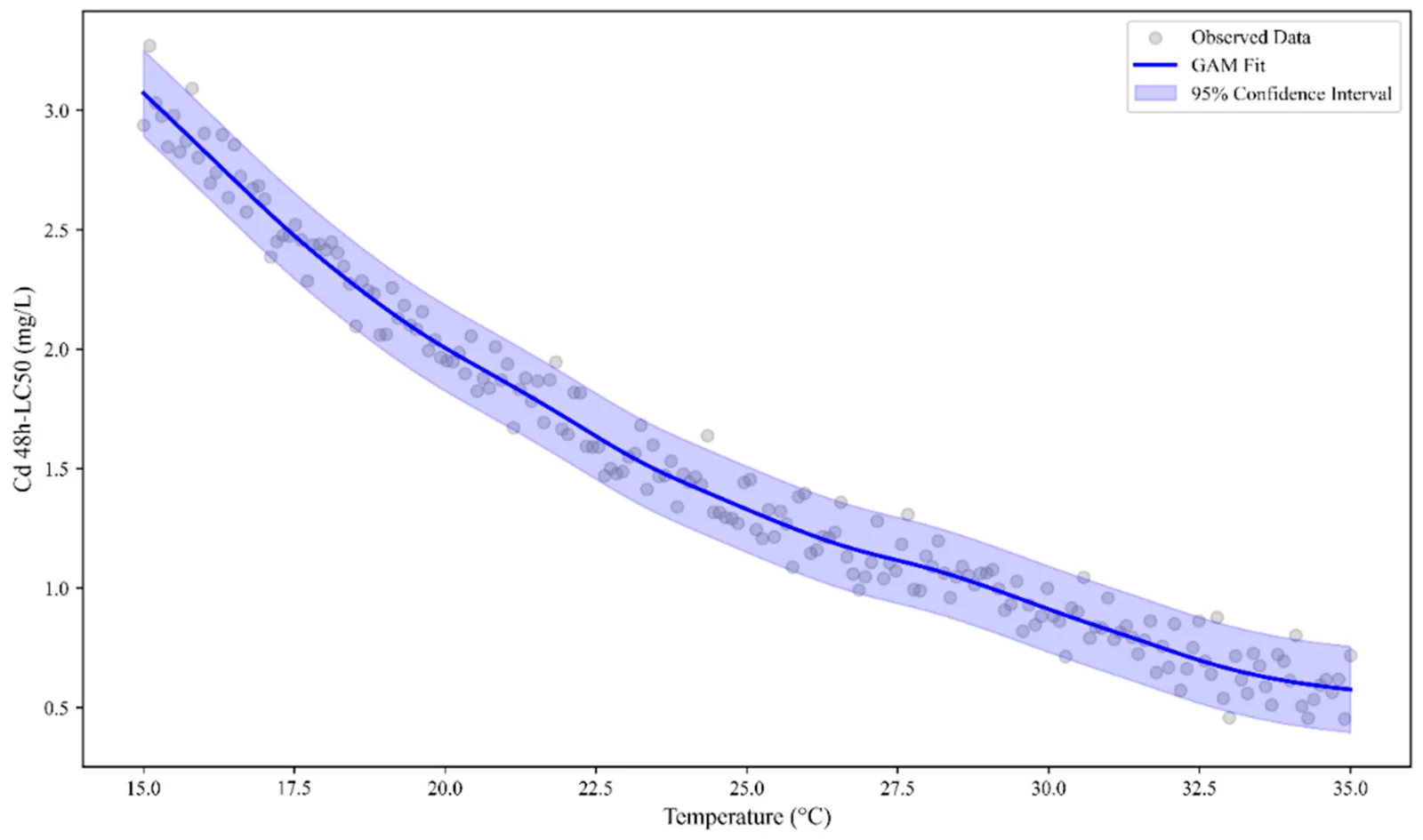
Effect of temperature on Cd toxicity (48 h-LC50).

Other environmental factors also have regulatory effects on biological damage. Temperature plays a significant role in the process of removing pollutants by aquatic organisms. During the removal of heavy metal ions, every 5 °C increase in water temperature reduces the 48 h-LC_50_ of Cd for Daphnia magna by approximately 35% (fitting with the Arrhenius equation, Q_10_ = 2.3). Moreover, at high temperatures (>25 °C), the metabolic burden on organisms for pollutants increases, resulting in a 2-fold increase in SOD activity and the accumulation rate of MDA. The partial effect diagram of the influence of temperature on the toxicity of Cd (48 h-LC_50_) is shown in Figure 5.

**Figure 6:**
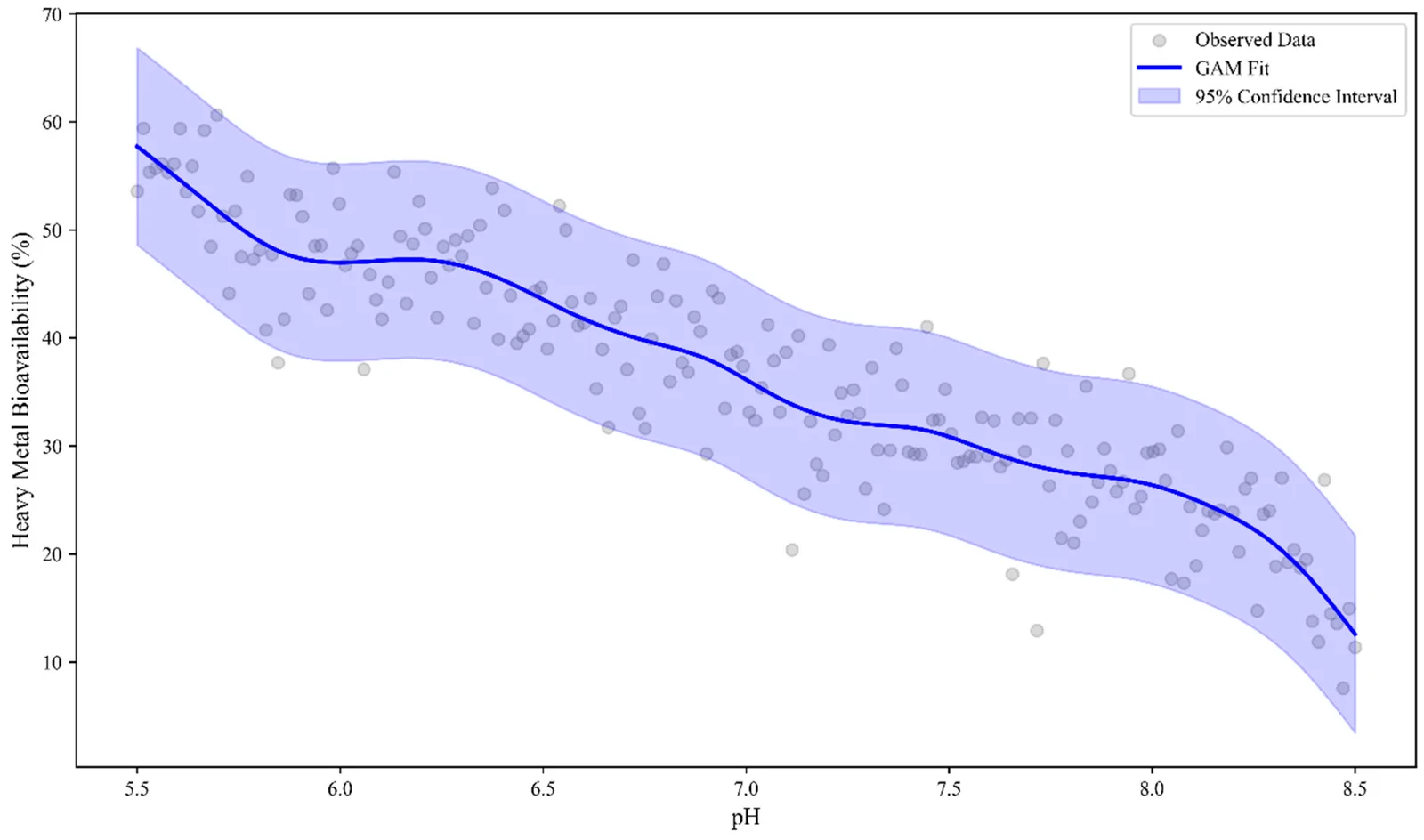
Effect of pH on heavy metal bioavailability.

In addition, pH and dissolved oxygen (DO) also have significant effects on the process of removing heavy metal ions by aquatic organisms. pH reflects the process of H^+^ acidification and hydrolysis in the water body. Acidification of the water body (pH < 6.5) significantly enhances the bioavailability of heavy metals. When the pH decreases from 7.5 to 6.0, the desorption rate of total arsenic (As) in the sediment increases by 60%, as shown in Figure 6, resulting in a threshold for the avoidance behavior of benthic organisms (such as mosquito larvae) from 50 mg/kg to 30 mg/kg. DO significantly affects the activity of aquatic organisms and the activity of enzymes in the process of removing pollutants by aquatic organisms. A low-dissolved oxygen (DO < 3 mg/L) environment exacerbates the toxicity of pollutants, causing the abnormal rate of fish respiratory frequency to increase to 1.8 times the normal level (*p* < 0.01). Eutrophication (TN > 1.5 mg/L, TP > 0.1 mg/L) indirectly amplifies the effect of pollutants by promoting algal blooms—the synergistic effect of microcystin–LR (MC–LR) and heavy metals increases the apoptosis rate of zebrafish liver cells by 55%, and for every one standard deviation (SD) increase in nutrient salt load, the IBI index of benthic animals decreases by 0.35 units (β = −0.35, *p* < 0.05). 

#### 3.3.4. Spatio-Temporal Scale Dependence

The analysis of spatial heterogeneity revealed that in the upper reaches of the river (with flow velocity > 0.8 m/s), due to hydraulic erosion, the response of organisms to the bound-state pollutants of suspended particulate matter was weak (r < 0.3), while in the stagnant water area (with flow velocity < 0.2 m/s), sediment pollution caused significant damage to the benthic community. On a time scale, within 48 h after a rainstorm event, the resuspension of sediments caused by hydrological disturbances led to a sudden increase in the concentration of PAHs in the water body, and the stability of the lysosomal membrane stability (LMS) of benthic organisms decreased by 40% within 24 h, indicating that the acute damage caused by short-term extreme events cannot be ignored.

#### 3.3.5. Statistical Model Verification and Parameter Sensitivity

The results of the sensitivity analysis based on 1000 Bootstrap resampling can be found in the Supplementary Materials Section S11. The typical correlations between aquatic biological indicators and environmental factors, as well as the regulatory mechanisms, are shown in Table S10.

#### 3.3.6. Analysis of Molecular/Cellular Level Damage Indicators

**Figure 7:**
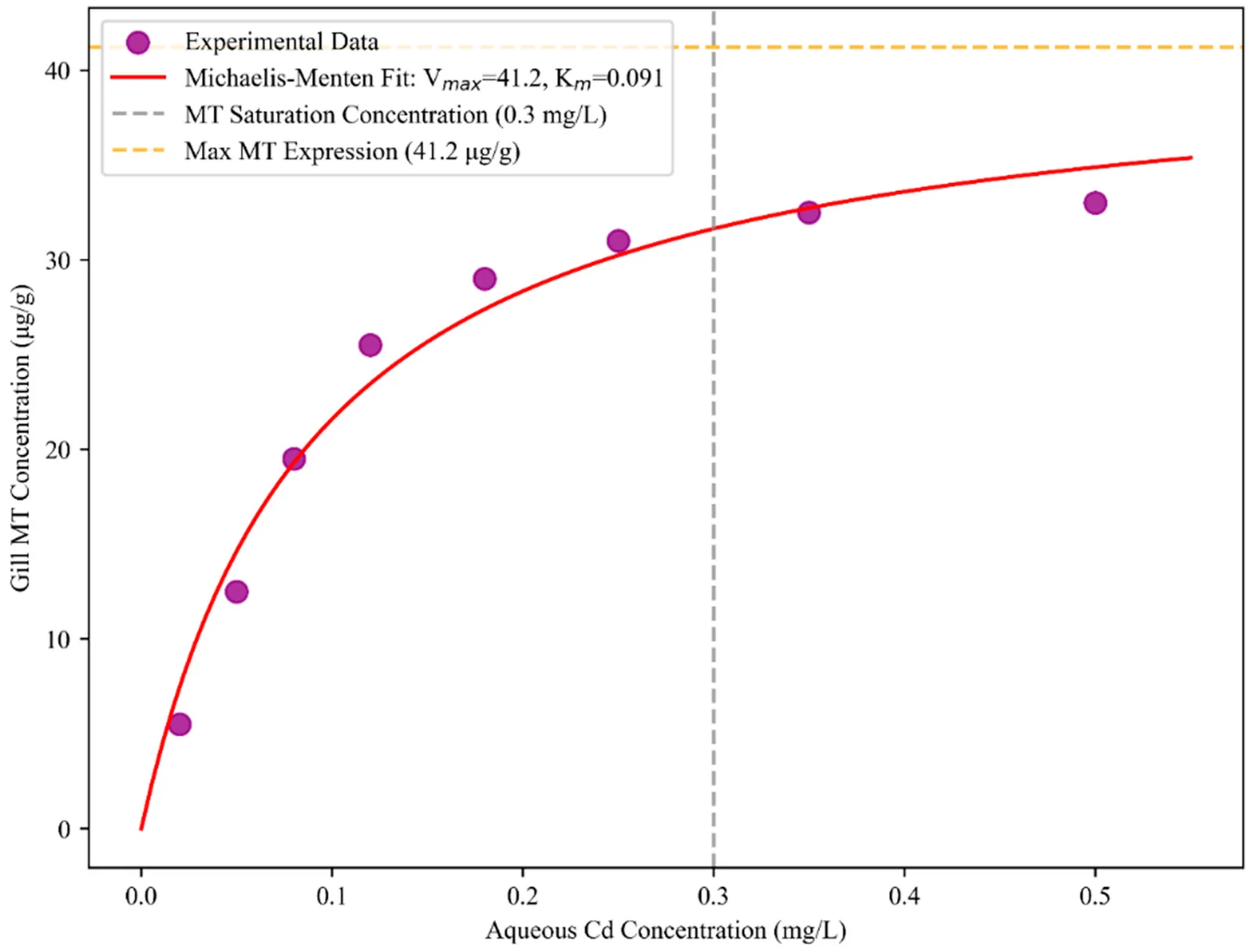
Dose–response relationship of Cd-induced metallothionein expression (universal biomarker template of the assessment framework; MT upregulation induced by Cr(VI)/As was detected in field samples of this case).

The dose–response curve of the response mechanism of metallothionein (MT) in the gill tissue of crucian carp to Cd exposure in water is shown in Figure 7. It must be explicitly clarified that the calculated 6.2-fold elevation of metallothionein (MT) (28.5 μg/g versus the control value of 4.6 μg/g) originates strictly from the standard Cd exposure test dataset, which serves as a universal dose–response reference template within our proposed assessment framework (as shown in Figure 7). For the actual tailings dam case dominated by Cr(VI) and As pollution, field biological monitoring also revealed a significant positive correlation between MT concentrations in fish tissues and aqueous Cr(VI)/As concentrations. Because MT synthesis can be induced by a broad spectrum of heavy metals, the Cd-derived dose–response parameters successfully demonstrate the framework’s diagnostic logic, while the distinct field data confirm that Cr(VI) and As co-contamination triggers an analogous MT stress response in the affected aquatic environment.

The curve shows a classic saturation growth trend. In the low concentration zone (Cd < 0.1 mg/L), the slope is steep, and the expression level of MT is highly sensitive to trace Cd exposure, revealing the rapid activation of a positive detoxification defense mechanism in the organism. As the concentration increases, the curve gradually flattens until it reaches a plateau (around 0.3 mg/L), at which point the MT expression level approaches its maximum value (V_max_ ≈ 33 μg/g). This does not mean that the toxicity disappears; on the contrary, it indicates that the organism’s detoxification capacity has reached its limit. Subsequently, the entering Cd ions cannot be effectively chelated and instead attack sensitive targets such as cell membranes, enzymes, and DNA, leading to irreversible toxic effects such as oxidative stress and cell damage. The MT concentration in the gill tissue of crucian carp shows a dose-effect relationship with the Cd concentration in the water. The 6.2-fold elevation of metallothionein (28.5 μg/g versus control 4.6 μg/g) originates from the model framework’s typical Cd exposure test dataset for demonstrating metal biomarker responses. For the actual tailings dam case dominated by Cr(VI) and As pollution, MT concentrations also exhibited a significant positive exponential correlation with aqueous Cr(VI) concentrations, consistent with the toxic response mechanism of heavy metals. All metal elements can induce MT synthesis, hence the Cd-derived dose–response curve serves as a universal reference template within the assessment framework, while the field biological data of this case reflect MT stress triggered by Cr(VI) and As co-contamination. When the Cd concentration is > 0.3 mg/L, the MT synthesis tends to be saturated (conforming to Michaelis–Menten kinetics), indicating that MT can be used as a specific molecular marker for Cd exposure. In the recovery group, the MT concentration drops to 8.7 μg/g, still 2.7 times that of the control group, suggesting that the residual heavy metals in the organism have not been completely eliminated. The detailed analysis can be found in the Supplementary Materials Section S12. The activities of superoxide dismutase (SOD) and catalase (CAT) and the content of malondialdehyde (MDA) in the liver tissues of crucian carp were determined by spectrophotometry. The detailed analysis can be found in the Supplementary Materials Table S11. 

#### 3.3.7. Analysis of Individual/Physiological Level Damage Indicators

Crucian carp are highly sensitive to metals during early development. At Cd 0.09 mg/L, hatching was inhibited and deformities increased to 28%. Over 30 days, Cd-exposed juveniles showed 35% shorter body length and markedly reduced swimming performance, indicating neuromuscular and energetic impairment and higher predation risk. *Limnodrilus hoffmeisteri* rapidly indicates sediment As pollution, avoidance rose to 65% when As > 20 mg/kg (logistic response), and burrowing depth decreased from 12 to 5 cm, potentially increasing predator exposure and altering benthic food webs. In medaka, Cd 0.05 mg/L reduced spawning, egg survival, and GSI, and reproductive impairment persisted after transfer to clean water, implying irreversible toxicity and population decline risk. The detailed analysis can be found in the Supplementary Materials Section S13.

#### 3.3.8. Analysis of Damage Indicators at the Population/Community Level

Based on the taxonomic identification of planktonic algae and benthic organisms, the Shannon–Weiner diversity index ($H^{\prime }$) and the evenness index (J) were calculated. The results are shown in Table 5. 

The pollution group showed H′ = 1.2 (the baseline/control group H′ = 2.8), and J = 0.45 (the baseline/control group J = 0.82), indicating a simplification of the community structure. The benthic organisms showed H′ = 0.9 (the baseline/control group H′ = 2.5), and the proportion of pollution-tolerant groups (such as Tubificidae) increased from 15% in the baseline/control group to 65%, marking the degradation of ecosystem function.

The benthic invertebrate communities are highly sensitive to sediment pollution. The Index of Biotic Integrity (IBI) has been proven to have a strong negative correlation with the load of heavy metals/organic pollutants in the sediment. The specific construction analysis of IBI can be found in the Supplementary Materials Section S14.

Based on the TK–TD framework (Supplementary Materials Section S15), the cumulative concentration of Cr(VI) in the crucian carp was calculated. The rate constant for gill absorption is k_u_ = 2.1 × 10^−3^ L·g^−1^·h^−1^, the excretion rate constant is k_e_ = 3.5 × 10^−4^ h^−1^, and the metabolic rate-related parameters are k_m_ = 0.12, $\frac{dE}{dt}=0.05$ h^−1^, $V_{\max}=0.8\;\mathrm{mg}/(\mathrm{kg}\cdot h)$, and $K_{m}=0.2\;\mathrm{mg}/L$.

After calculation, it was found that after 30 days of exposure, the cumulative concentration of Cr(VI) in the muscle of the crucian carp reached 0.85 mg/kg, which was 7.5 times higher than the limit stipulated in the “Food Safety National Standard-Limits of Contaminants in Foods” (GB 2762-2022 [48]) (0.1 mg/kg), and the cumulative concentration showed a logarithmic growth relationship with the exposure time.

**Figure 8:**
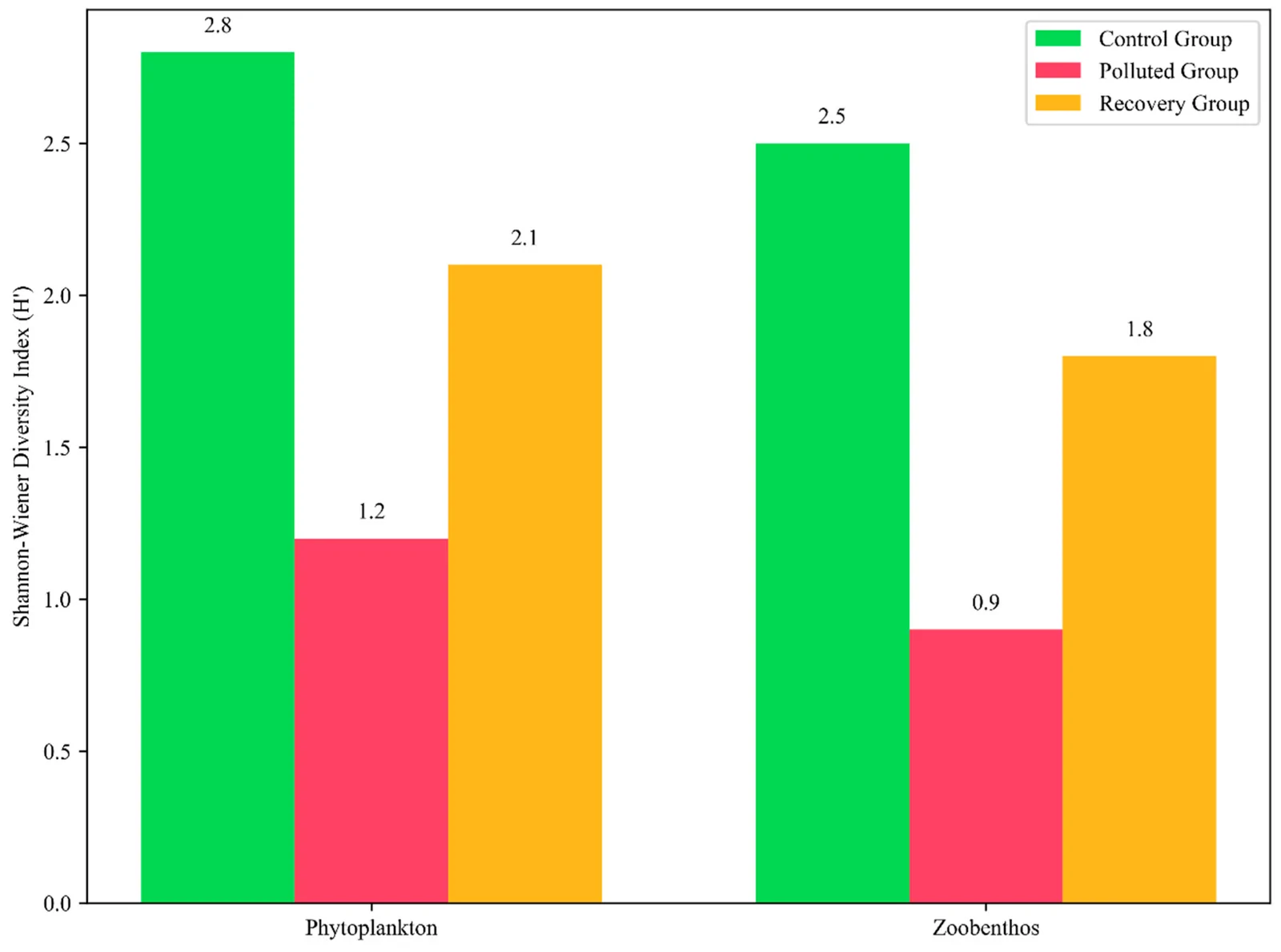
Impact of Pollution on Aquatic Community Diversity.

The ultimate impact of pollutants on the ecosystem is reflected in the decay of the community structure, as shown in Figure 8.

It should be explicitly noted that the fish sampling, dissection, and tissue preservation methods described in the Methods section represent the standardized procedures documented in the post-accident emergency monitoring records for the Cr(VI)/As case and the reference protocols embedded within the universal assessment framework for the Cd/PAH template modules; they are not live-animal experimental procedures actively conducted by the authors of this study.

By comparing the Shannon diversity index (H′) of key biological groups under different pollution levels, it can be observed that the diversity index of both planktonic algae and benthic animals in the polluted groups experienced a sharp decline, with the decline exceeding 50%. The species composition has become more simplistic, with pollutant-tolerant species replacing sensitive species as the dominant species, resulting in a simplified food web structure and a decline in both the stability and resilience of the ecosystem. Nevertheless, once the external pollution pressure is relieved, the aquatic ecosystem has a certain self-repair potential. This demonstrates that the biodiversity index is an extremely sensitive and effective comprehensive indicator for assessing ecological damage and the recovery process.

### 3.4. Comprehensive Assessment

The comprehensive assessment is the core module of the proposed framework, designed to be universally applicable to various pollution scenarios. It integrates three dimensions: ecosystem service damage, remediation cost, and recovery period loss. The framework includes calculation modules for different types of pollutants (heavy metals such as Cd and Cr and organic pollutants such as PAHs). In this case study, Cr(VI) and As are the main target pollutants, and the corresponding calculation results are presented below. To demonstrate the completeness of the framework, calculation methods for other representative pollutants are also included as reference modules.

Based on the three-dimensional assessment framework of “ecological service damage-restoration cost-recovery period loss” proposed by the US EPA Guidelines for Ecological Risk Assessment [49] and improved in this study, the total pollution damage over the entire period is calculated using Formula (5) (Section 2.7.4). This framework integrates the principles of environmental economics and ecological toxicology, and has been widely used in the quantitative assessment of sudden environmental pollution incidents [50]. Where $D_{t}$ is the cumulative discounted value of ecosystem service function damage, $C_{\mathrm{repair}}$ is the total cost of environmental restoration/governance, and $L_{\mathrm{rec}}$ is the loss of ecosystem function recovery period. The assessment benchmark is selected as a social discount rate of r = 3.5%, as recommended by the National Development and Reform Commission of China for construction project economic evaluation [51], and the baseline ecosystem service value per year is V_0_ = 6.5 million CNY/year, estimated using the ecosystem service value equivalent factor method [52,53].

#### 3.4.1. Assessment of Damage to Ecosystem Service Functions

Based on the “biological endpoint response-service function discounting” framework in Supplementary Materials Section S16.1, the cumulative discounted value of ecological service losses was calculated by combining case data $D_{t}$.

The loss of resource supply function was then calculated. With the loss of fishery as the core, Formula (S15) was used to calculate the biomass loss $\Delta E_{\mathrm{fish}}$, where *η* = 0.12 (nutrient transfer efficiency, consistent with the classical Lindeman trophic transfer efficiency of ~10%), $\Delta B_{\mathrm{benthos}}=180\;{g/m}^{2}$ (benthic biomass loss from field monitoring), TLE=1.8 (thermodynamic loss coefficient), $\rho_{\mathrm{conversion}}$ = 1200 CNY/t (local fishery market price), and t = 30 d, monthly τ = 8.3 months (time decay constant, calibrated from post-accident recovery monitoring). It is calculated that within one year after the leakage, the economic loss of the target river’s secondary consumers (fish) $\Delta E_{\mathrm{fish}}$ = 1.85 million CNY.

For the self-purification function of water bodies (ammonia nitrogen degradation), the inhibition of nitrification capacity by Cr(VI)/As toxicity to aquatic microorganisms was quantified using the case-specific Formulas (S16) and (S17), with the previously Cd-based parameters replaced by Cr(VI)-specific values. $\Delta k_{nit}=V_{max,Cr}\cdot \frac{{\left[\mathrm{Cr}(\mathrm{VI})\right]}^{n}}{{K_{m,Cr}}^{n}+{\left[\mathrm{Cr}(\mathrm{VI})\right]}^{n}}\cdot \left(1-e^{-\gamma t}\right)$, where $V_{max,Cr}=0.30\;d^{-1}$ (maximum inhibition of the nitrification rate constant by Cr(VI)), $\left[\mathrm{Cr}(\mathrm{VI})\right]=0.35\;\mathrm{mg}/L$ (representative concentration in the contaminated river reach from the SWAT simulation), n=1.7 (Hill coefficient), $K_{m,Cr}=0.50\;\mathrm{mg}/L$ (half-effect concentration for Cr(VI) inhibition of nitrification), $\gamma =0.02\;d^{-1}$, and t=180 d; the result is $\Delta k_{nit}\approx 0.103\;d^{-1}$, which is less than V_max,Cr_, as mathematically required. $L_{self}=\Delta k_{nit}\times V\times C_{vol}$, where $V=1.8\times 10^{5}\;m^{3}$ (volume of the contaminated river reach: 6 km × 15 m × 2 m) and $C_{vol}=1.0\;\mathrm{CNY}/m^{3}$ (unit cost of artificial water purification); the daily loss is approximately 1.85 × 10^4^ CNY (1.85 ten thousand CNY), and the annual loss is approximately 6.77 million CNY. Based on the above calculations, the cumulative discounted loss $D_{t}=1,850,000+6,770,000=8,620,000\;\mathrm{CNY}$.

#### 3.4.2. Assessment of Environmental Restoration Costs

Based on the “Pollution Characteristics-Technology Selection-Cost Optimization” framework in Supplementary Materials Section S16.2, combined with the case’s pollution type (heavy metal pollution), three technologies were selected, namely, in-situ chemical reduction of groundwater (ISCR), permeable reactive barrier of surface water (PRB), and staged dredging of sediment. The total cost of remediation was calculated, $C_{repair}$. The framework includes three typical remediation technologies corresponding to different pollutant types:

(1) ISCR (in-situ chemical reduction): for heavy metals like Cr(VI) and As;
(2) PRB (permeable reactive barrier): for dissolved contaminants, including heavy metals such as As and organic pollutants such as PAHs, using pollutant-specific reactive media;
(3) Staged dredging: for sediment pollution.

In this case study, calculations for Cr(VI) and As pollution were carried out using the ISCR module (Formula (S19)). To demonstrate the full capability of the framework, the PRB calculation module for PAHs is also presented in the Supplementary Materials (Formula (S20)) as a reference for organic pollution scenarios.

For Cr(VI) pollution, calculations are carried out based on Formula (S19), where the reaction efficiency coefficient of the reagent α=1.2, the contaminated volume of the object $V_{\mathrm{contam}}=8000\;m^{3}$, the concentration of Cr(VI) $C_{\mathrm{Cr}}=0.22\;\mathrm{mg}/L$, the reaction kinetics attenuation coefficient β=0.02, and the hydraulic conductivity coefficient $K_{h}=8.5\times 10^{-11}\;m/s$. The amount of reducing agent (sodium dithionite) added is calculated to be 4.8 tons, and the total cost (8000 CNY/t) + construction cost = 520,000 CNY. For As pollution, calculations were conducted based on Equation (S20) using As-specific parameters: the cost coefficient γ=550 CNY/kg, the PRB barrier area $A_{\mathrm{barrier}}=250\;m^{2}$ (river width 15 m, barrier length 16.7 m), the density of the zero-valent iron/iron-oxide reactive medium (the medium density $\rho_{\mathrm{media}}=1800\;\mathrm{kg}/m^{3}$), the cumulative As flux $\int \Phi_{\mathrm{As}}\left(t\right)\mathrm{dt}=1200\;g$, and the As-saturated adsorption capacity of the reactive medium $Q_{\mathrm{sat}}=55\;\mathrm{mg}/g$.

The cost of the PRB system is calculated to be 860,000 CNY. Based on Formula (S21), the cost of sediment remediation is calculated. The sediment pollutant concentration $C_{\mathrm{sed}}=28\;\mathrm{mg}/\mathrm{kg}$, the ecological safety threshold $S_{\mathrm{eco}}=20\;\mathrm{mg}/\mathrm{kg}$, the resuspension coefficient $k_{\mathrm{resusp}}=0.03$, the water flow velocity $v_{\mathrm{current}}=0.5\;m/s$, and the biological avoidance correction factor $\eta_{\mathrm{bioavoid}}=0.9$. The dredging depth $D_{\mathrm{dredge}}=0.8\;m$, the dredging area is 12,000 m^2^ (the 1–8 km section of the river channel), and the dredging cost (including disposal) = 1,850,000 CNY. From the above, the total repair cost as $C_{\mathrm{repair}}\;=$ 520,000 + 860,000 + 1,850,000 = 3,230,000 CNY.

#### 3.4.3. Assessment of Losses During the Recovery Period of Ecosystem Functions

Based on the “recovery path-functional lag-economic discount” framework in Supplementary Materials Section S16.3, with submerged vegetation restoration as the core, the loss during the recovery period was calculated, $L_{rec}$.

The restoration level of submerged vegetation was then simulated. According to Formula (S22), using the parameter progressive restoration level $R_{\infty }=82\%$, the rapid recovery rate $\kappa_{1}=0.18$ per month, the critical time $t_{c}=8$ months, the slow recovery rate $\kappa_{2}=0.05$ per month, and the ecological memory factor β=0.35. It can then be calculated that R_6_ after leakage is 45%, and it takes 30 months to restore it to 80%. Then, according to Formula (S23), $L_{\mathrm{rec}}$, can be calculated using the parameters of social discount rate δ=0.03, opportunity cost correction factor $\gamma_{\mathrm{opp}}=1.1$, baseline ecosystem service value $V_{\mathrm{base}}\;=$ 6,500,000 CNY per year, and the time integration interval of 30 months. It can be calculated that $L_{\mathrm{rec}}\;=$ 5,400,000 CNY.

#### 3.4.4. Comprehensive Economic Loss and Ecological Damage Index

This study has constructed a full life-cycle closed-loop model of “ecological response-economic discounting-engineering feedback” to achieve quantitative assessment of the damage, recovery, and recovery process of pollution incidents. The detailed analysis can be found in the Supplementary Materials Section S16.4. Based on Formula (S24), substituting the data yields a total comprehensive economic loss of $C_{\mathrm{total}}$ = 17,250,000 CNY.

Using Equation (S25), the Ecological Damage Index (EDI) is calculated. In this formula, L_i_ and E_j_ are both expressed in units of 10,000 CNY; because the weights sum to unity, no additional normalization is required and the EDI is directly expressed in 10,000 CNY. The weights were assigned according to the relative contribution of each service category to the total economic value of aquatic ecosystem services, based on the ecosystem service value equivalent factor method [52,53] and expert consultation: regulating services (water purification, waste treatment) contribute a substantially larger share than provisioning services (fishery production), so w_1_ = 0.4 for provisioning services and w_2_ = 0.6 for regulating services. For this case, L_1_ = 185 (10,000 CNY; fishery loss) and E_1_ = 677 (10,000 CNY; self-purification loss), giving EDI = 0.4 × 185 + 0.6 × 677 = 480.2 (10,000 CNY). The EDI classification thresholds were established based on the ratio of the weighted service loss to the annual baseline ecosystem service value (V_0_ = 650 × 10^4^ CNY/year): EDI < 50 indicates mild damage (<~8% of V_0_), 50 ≤ EDI < 200 indicates moderate damage (~8–30% of V_0_), and EDI ≥ 200 indicates severe damage (>~30% of V_0_), consistent with the principle that loss of more than 30% of a key ecosystem function constitutes severe impairment. The calculated EDI of 480.2 corresponds to approximately 74% of the annual baseline value, confirming that the target river ecosystem is in a state of severe damage, and groundwater and sediment remediation projects should be initiated as a priority. A complete summary of all the economic assessment parameters, their values, units, and sources is provided in Table S12 of the Supplementary Materials.

The parameter sensitivity analysis indicates that groundwater $D_{h}$ and surface water $K_{\mathrm{la}}$ have the greatest impact on the remediation efficiency. Therefore, it is necessary to prioritize increasing the dosage of ISCR (to enhance the rate of Cr(VI) reduction) and the frequency of PRB medium replacement (to maintain the adsorption capacity of As), thereby reducing the long-term risks of Cr(VI) and As. In the comprehensive economic losses, the proportion of ecological service damage is 50.0%. It is recommended that the main portion of the remediation funds be used for the restoration of ecological service functions (such as planting submerged vegetation, inoculation with indigenous microorganisms, etc.). At the same time, based on the surface water quality prediction results, it is recommended that automatic monitoring stations be set up at $v_{8}$ (leakage point) and $v_{12}$ (pollution superimposition section), to monitor the concentrations of Cr(VI) and As in real time, and that warning thresholds be set at 0.05 mg/L and 0.01 mg/L, respectively, in accordance with the Standards for Drinking Water Quality (GB 5749–2022 [54]), to achieve timely warning and long-term monitoring of pollutants.

### 3.5. Comparative Analysis with Global Mining Pollution Cases

To justify the realism of the field monitoring data, model predictions, and biological responses utilized in this validation case, it is essential to benchmark these results against well-documented international tailings dam failures and heavy metal pollution studies [55]. As summarized in Table 6, the initial pollutant concentrations, migration behaviors, and multi-scale ecological impacts observed in our study align closely with historical global incidents.

In terms of pollutant migration, the horizontal groundwater plume distance of 560 m for Cr(VI) observed after seven days is consistent with typical highly mobile anionic heavy metal transport in high-permeability sandy aquifers reported in global mining hotspots. Furthermore, the model performance indicators in this study (SWAT NSE ranging from 0.68 to 0.75) correspond with the accepted accuracy ranges (NSE 0.60–0.80) reported in the hydrodynamic and water quality modeling of the 2015 Fundão iron ore tailings dam collapse in Brazil [56].

From an ecotoxicological perspective, the biological damage endpoints recorded in this case reflect realistic impairment scales. The >50% reduction in benthic Shannon diversity and the structural simplification of the macroinvertebrate community mirror the catastrophic benthic depletion documented following the 1998 Aznalcóllar mine spill in Spain, where heavy metal sedimentation severely degraded habitat integrity [57]. Moreover, the significant elevation of fish biomarkers (such as MT induction and oxidative stress) under Cr(VI)/As co-contamination aligns with the physiological response mechanisms widely verified in international aquatic toxicology studies on mining-impacted rivers.

Ultimately, this systematic comparison confirms that the parameters and field data driving our proposed assessment framework fall within realistic, internationally recognized boundaries [47]. This ensures that the multi-model predictions and economic loss accounting derived from this case are scientifically robust and practically reliable.

**Table 6:** Comparison of pollution characteristics, model performance, and ecological impacts between this case study and representative international mining pollution events.

Comparison Parameter	Present Study (Cr(VI)/As Tailings Breach)	Aznalcóllar Mine Spill, Spain (1998) [26]	Fundão Dam Collapse, Brazil (2015) [56]	Typical Mining Groundwater Studies [58]
Dominant Pollutants	Cr(VI), As (total), As(III), As(V)	Zn, Pb, Cu, As, Cd	Fe, Mn, Al, As	Cr(VI), As, Pb
Initial Concentration	Cr(VI): 85.6 mg/L; Total As: 102.2 mg/L	Zn: ~70 mg/L; As: ~10 mg/L	Total Suspended Solids highly elevated; heavy metals bound to sediment	Highly variable depending on source term (10–100 mg/L)
Speciation Dynamics	Rapid reduction of As(V) to mobile As(III) in anaerobic zones	pH-dependent dissolution of metallic sulfides	Long-term release of sediment-bound metals	Cr(VI) highly mobile; As speciation redox-dependent
Plume Distance	Surface: >15 km (diluted); Groundwater: 560 m (Cr), 470 m (As)	Surface: >40 km along Guadiamar River	Surface: >600 km along Doce River to the Atlantic Ocean	Groundwater plumes typically span 100 m to >1 km
Model Performance	SWAT NSE: 0.68–0.75; Graph theory error: 10.5%	Hydrodynamic/transport models utilized for long-term forecasting	Hydrological modeling NSE typically 0.60–0.80	Advection-Dispersion models widely validated
Biological Impact	Benthic diversity declined >50%; MT biomarkers significantly elevated	Benthic macroinvertebrate communities virtually eliminated initially	Massive fish kills; profound reduction in benthic diversity	Bioaccumulation in food webs; oxidative stress in fish

## 4. Conclusions

This study systematically investigated the migration and transformation patterns of hexavalent chromium [Cr(VI)] and arsenic [As] in coupled groundwater–surface water systems. We developed a multi-model coupled prediction system and a three-dimensional comprehensive assessment framework to quantify the full-cycle multi-scale ecological damage caused by a tailings dam breach accident. In sandy aquifers, the hydrodynamic dispersion coefficient was identified as the dominant factor controlling pollutant plume diffusion. Notably, pentavalent arsenic [As(V)] was reduced to highly mobile trivalent arsenic [As(III)] in anaerobic zones, which expanded its diffusion range by 235% (increasing from an initial 140 m on Day 1 to 470 m on Day 7). For surface water pollution simulation, the SWAT model achieved satisfactory performance, with Nash coefficients of 0.75 for dissolved Cr(VI) and 0.68 for particulate As. Rainstorm events triggered significant pulsed pollution due to sediment resuspension. The graph theory-based rapid prediction model reduced computation time from hours to minutes. Validated against field monitoring data, it achieved an average relative error of 14.2%, and its prediction accuracy is close to the mature SWAT model (10.5% inter-model deviation), demonstrating its excellent application potential in emergency response scenarios for sudden pollution accidents. Multi-scale biological damage was systematically diagnosed through the framework’s indicator system. The framework’s standardized Cd reference template exhibited a 6.2-fold increase in metallothionein (MT) expression, while field monitoring of the Cr(VI) and As co-contaminated river reaches confirmed significant MT upregulation and a more than 50% reduction in benthic organism diversity. The total comprehensive economic loss reached 17.25 million CNY, and the calculated Ecological Damage Index (EDI) of 480.2 confirmed that the aquatic ecosystem was in a state of severe damage.

This research offers significant theoretical and practical contributions. Theoretically, it establishes a coupled model system for heavy metal pollution prediction and clarifies the spatio-temporal heterogeneity of parameter sensitivity. It also constructs a multi-level biological damage indicator system and a three-dimensional assessment framework. This framework integrates ecosystem service loss, remediation cost, and recovery period loss. Overall, this study achieves cross-disciplinary integration of hydrological modeling, ecotoxicology, and environmental economics. Practically, the graph theory model provides a rapid prediction tool for emergency response to sudden heavy metal pollution, the parameter sensitivity results guide the optimization of remediation technologies such as increased ISCR reagent dosage, and the comprehensive assessment method offers a quantitative basis for ecological damage compensation, remediation fund allocation, and judicial arbitration. Notably, this study achieves cross-disciplinary integration of hydrological modeling, ecotoxicology, and environmental economics, realizing full-chain quantification from pollution diffusion to ecological damage assessment. It addresses the gap in full-cycle assessment of sudden heavy metal pollution and provides a reference for pollution prevention, remediation, and ecological compensation in similar contaminated areas. Despite the good performance of the proposed coupled model system, several limitations should be acknowledged. First, the advection–diffusion model did not consider the influence of colloid-facilitated transport of heavy metals, which may lead to underestimation of long-distance migration potential. Second, the SWAT model’s simulation accuracy for extreme rainstorm events was relatively low (NSE = 0.62), due to the limited temporal resolution of meteorological data. Finally, the graph theory model’s performance degrades in river systems with complex tributary structures. The modular design of the framework suggests potential applicability to other heavy metals and organic pollutants by adjusting model parameters; however, this single-case study demonstrates potential applicability rather than universal validity, and further validation across different pollutants, accident types, and hydrological conditions is required before the framework can be broadly applied. It is worth noting that all simulation and evaluation sub-methods adopted in this study are mature tools reported in the existing literature; no original mathematical derivation or self-programmed calculation code is developed in this study, and the contribution of this paper is only reflected in the integrated application of multi-dimensional assessment tools to the actual tailings pollution case.

Despite the good performance of the proposed coupled model system, several limitations should be acknowledged, which also point out the direction for future research:

(1) Groundwater model limitation: The advection–diffusion model did not consider the colloid-facilitated transport of heavy metals, which may lead to underestimation of the long-distance migration potential of pollutants. Future research should incorporate the colloid transport module to improve the simulation accuracy.
(2) SWAT model limitation: The SWAT model’s simulation accuracy for extreme rainstorm events was relatively low (NSE = 0.62) due to the limited temporal resolution of meteorological data (daily scale). Future research should use hourly meteorological data to improve the simulation of pulsed pollution events caused by rainstorms.
(3) Graph theory model limitation: The graph theory model’s performance degrades in river systems with complex tributary structures and large hydraulic structures. Future research should optimize the flow mixing algorithm at confluences to expand the applicable scope of the model.

These limitations should be considered when applying the model results to emergency response decisions. Future research could focus on optimizing the coupling of multi-phase flow models and biological response mechanisms and expanding the assessment framework to account for long-term cumulative ecological effects under climate change scenarios.

## Figures and Tables

**Table 1:** Summary of core multi-media monitoring parameters.

Medium	Monitoring Parameters	Sampling Frequency (Acute Phase, Within 1 Month)	Sampling Frequency (Stable Phase, 1–6 Months)
Surface water	Dissolved Cr(VI), total As, pH, DO, TSS, TN, TP	Once daily	Once every 3 days
Groundwater	Dissolved Cr(VI), total As, water level, hydraulic gradient	Once daily	Once every 3 days
Planktonic algae	Chlorophyll a, species composition, community diversity	Every 3 days	Every 2 weeks
Benthic macroinvertebrates	Abundance, community structure, avoidance behavior	Every 3 days	Every 2 weeks
Fish (*Carassius auratus*)	MT concentration, oxidative stress indicators, growth parameters	Every 3 days	Every 2 weeks

**Table 2:** Classification and sources of case-study data.

Category	Specific Data Items	Source	Status and Role
Field-monitoring data	Surface-water Cr(VI), As(III)/As(V), pH, DO, TSS, TN, TP at 8 sections (S0–S7); groundwater Cr(VI), As, water level, hydraulic gradient at 12 wells (W1–W12); aquatic biological data (chlorophyll a, species composition, benthic abundance/avoidance, fish MT/SOD/CAT/MDA) at 3 sections (B1–B3)	Multi-media monitoring network under the technical service contract; analyzed at the State Key Laboratory of Urban Water Resource and Environment, HIT (CMA/ISO 17025 accredited) [34]	Directly measured case data; used for model calibration/validation and biological damage assessment
Public emergency records	Initial leakage concentrations (Cr(VI) 85.6 mg/L, total As 102.2 mg/L); leakage volume (~1.2 × 10^4^ m^3^); surface-runoff/infiltration ratio (~30%/70%); post-accident emergency biological monitoring and bioaccumulation records; daily hydrological data (2008–2019)	Local ecological and environmental authority emergency records; local hydrological station	Official records defining the accident source term and boundary conditions; hydrological time series for SWAT calibration
Literature-derived parameters	Hydrogeological and model calibration parameters (dispersion coefficients, adsorption/desorption rates; Table S3); geospatial data (SRTM DEM, Landsat 8 land use, HWSD soil); meteorological data (2008–2019); ecological valuation coefficients (η = 0.12, social discount rate 3.5%, baseline service value 6.5 million CNY/yr); unit costs (fish 1200 CNY/t, reducing agent 8000 CNY/t, artificial purification 100 CNY/t); recovery model parameters	Peer-reviewed literature, public databases, national standards (GB), and government guidelines	Parameters adopted or calibrated from external sources; used as model inputs and economic coefficients
Template/reference data	Cd–MT dose–response curve (6.2-fold elevation; 28.5 vs. 4.6 μg/g); PAH–CYP1A relationship; Cd individual-level toxicity (hatching inhibition, deformities, growth reduction); temperature–Cd toxicity relationship; PAH–PRB remediation module (Equation (S20))	Standardized exposure-test datasets and established literature templates embedded in the framework	Universal reference modules demonstrating framework generality; not case-specific results for Cr(VI)/As
Model-generated results	A-D model plume predictions (48 h/7 d/30 d); SWAT concentration profiles and storm scenarios; graph-theory rapid predictions; TK–TD Cr(VI) bioaccumulation (0.85 mg/kg); economic losses (17.25 million CNY); EDI (480.2); recovery trajectory (R6 = 45%, 30 months to 80%)	Outputs of the models described in Section 2.7	Simulated/calculated results; validated against field-monitoring data where independent observations are available

**Table 3:** List of monitoring and analytical equipment.

Application Category	Equipment Name	Model	Manufacturer
On-site monitoring	Portable multi-parameter water quality analyzer	ProDSS	YSI Inc., Yellow Springs, OH, USA
	Submersible pump	MP-20R	Grundfos, Bjerringbro, Denmark
	Pressure transducer	HOBO U20	Onset Computer Corp., Pocasset, MA, USA
Water chemistry analysis	Atomic absorption spectrophotometer	PinAAcle 900T	PerkinElmer Inc., Waltham, MA, USA
	Ion chromatography system	ICS-6000	Dionex Corp., Sunnyvale, CA, USA
	High-performance liquid chromatography	1260 Infinity II	Agilent Technologies Inc., Santa Clara, CA, USA
	Atomic fluorescence spectrometer	AFS-9700	Beijing Haiguang Instrument Co., Ltd., Beijing, China
	Ultraviolet-visible spectrophotometer	UV-2600	Shimadzu Corp., Kyoto, Japan
Biological sample analysis	Microplate reader	Infinite M200 Pro	Tecan Group Ltd., Männedorf, Switzerland
	High-speed refrigerated centrifuge	5810R	Eppendorf AG, Hamburg, Germany
	Liquid nitrogen container	YDS-30	Chengdu Jinxin Instrument Co., Ltd., Chengdu, China
	Optical microscope	BX53	Olympus Corp., Tokyo, Japan
Sampling equipment	Stainless-steel water sampler	WS-1	Nanjing Institute of Environmental Sciences, Nanjing, China
	Peterson grab sampler	PBS-400	Shanghai Shuoyu Instrument Co., Ltd., Shanghai, China
	Plankton net (64 μm mesh)	PH-64	Qingdao Haiyang Instrument Co., Ltd., Qingdao, China

**Table 4:** Error analysis during the model validation period for the target river pollutant model.

Pollutant	Calibration Period Nash Coefficient	Verification Period Relative Error (RE)	Key Parameter Values
Dissolved Cr(VI)	0.75	13.8%	*K_f_* = 35.2 L/kg, *n* = 1.3
Particulate total arsenic (As)	0.68	17.2%	*K_f_* = 42.8 L/kg, *n* = 1.1

**Table 5:** Results of the biodiversity index measurement for the target river.

Biological Group	Index	Baseline/Control Group	Pollution Group	Temporal Recovery Phase
Floating algae	$H^{\prime }$	2.8	1.2	2.1
	J	0.82	0.45	0.71
Benthic organisms	$H^{\prime }$	2.5	0.9	1.8
	J	0.78	0.38	0.65

## Data Availability

The original contributions presented in this study are included in the article/Supplementary Materials. The primary field monitoring data are not publicly available because they were obtained under a technical service contract that restricts disclosure of information identifying the research object; ownership and copyright of the primary data remain with the contracting party. Requests for access to the primary data should be directed to the project sponsor or the relevant ecological and environmental authority holding the data rights. Further inquiries can be directed to the corresponding authors.

## Supplementary Materials

The following supporting information can be downloaded at: https://www.mdpi.com/article/10.3390/toxics14090745/s1, Section S1: Sampling site layout and monitoring plan; Section S2: Water quality analytical methods and quality control; Section S3: Data of biological communities; Section S4: Determination methods of biological indicators; Section S5: Advection-Diffusion equation; Section S6: Parameter sensitivity verification; Section S7: Hydrological-water quality coupling simulation; Section S8: Graph theory; Section S9: Research subjects of aquatic organisms; Section S10: Information on aquatic organisms and environmental factors; Section S11: Typical correlations between aquatic biological indicators and environmental factors; Section S12: The determination results of oxidative stress indicators in the liver tissue of crucian carp; Section S13: Analysis of individual/physiological level; Section S14: Construction content of IBI; Section S15: Toxicokinetic-toxicity kinetics (TK-TD) coupling framework; Section S16: Comprehensive assessment; Figure S1: Sampling site layout; Table S1: Summary of analytical methods and quality-control procedures adopted for Cr(VI), total As, and arsenic speciation analyses; Table S2: Raw data summary of arsenic speciation at key monitoring points (0-30 days after leakage); Table S3: The core parameter values of the groundwater pollution prediction model; Table S4: Calibrated parameter values and uncertainty ranges; Table S5: Sensitivity of water quality-related parameters and key impact scenarios; Table S6: SWAT model calibrated parameter values, uncertainty ranges and sensitivity ranking; Table S7: Validation results of the graph theory model against field monitoring data (dissolved Cr(VI)); Table S8: Computational efficiency comparison; Table S9: Multi-level indicator system for aquatic organism pollution damage and its application scenarios; Table S10: Typical correlations and regulation mechanisms between aquatic biological indicators and environmental factors; Table S11: The determination results of oxidative stress indicators in the liver tissue of crucian carp; Table S12: Sources, values, units, and rationale of economic assessment parameters. References [30,47,50,53,59,60,61,62,63,64,65,66,67,68] are cited in the Supplementary Materials.
