# Supplementary material for: Full-Cycle Ecological Damage Assessment Framework for Sudden Water Pollution Accidents: Multi-Model Coupled Prediction and Three-Dimensional Quantitative Evaluation with a Case Study of Tailings Dam Breach
Source: Toxics. 2026 Aug 23;14(9):745. doi: 10.3390/toxics14090745 (PMC13611259; doi:10.3390/toxics14090745)
Supplement: Supplementary file 1 [file toxics-14-00745-s001.zip › toxics-4438352-supplementary.pdf]

Full-Cycle Ecological Damage Assessment Framework for Sudden Water Pollution Accidents: Multi-Model Coupled Prediction and Three-Dimensional Quantitative Evaluation with a Case Study of Tailings Dam Breach

Zhengda Lin [1,*], Xinhao Sun [1], Bingjie Yan [1] and Caoqingqing Li [2,*]

[1] State Key Laboratory of Urban Water Resource and Environment, School of Environment, Harbin Institute of Technology, Harbin 150090, China; sunxinhao2001@163.com (X.S.); 18654872775@163.com (B.Y.)

[2] Heilongjiang Research Academy of Environmental Sciences, No. 3 Haixing Street, Nangang District, Harbin·150090, China

* Correspondence: linzhengda@hit.edu.cn (Z.L.); licaoqingqing@163.com (C.L.); Tel.: +86-18845027393 (Z.L.); +86-17645170822 (C.L.)

Section S1 Sampling site layout and monitoring plan

In this study, a multi-media environmental sampling and monitoring program was designed. For surface water, eight monitoring sections were set up, focusing on the determination of dissolved and particulate concentrations of Cr(VI) and As, and the simultaneous determination of key hydrological parameters, such as pH, dissolved oxygen (DO) and total suspended solids (TSS). For groundwater, 12 monitoring wells (well depths of 6-15 m) were set up within a range of 2 km around the tailings pond to monitor Cr(VI) and As concentrations, and at the same time to obtain data on the groundwater level and hydraulic gradient. For aquatic organisms, three key sections, namely, the core area of the polluted area, the middle buffer zone and the downstream restoration area, were selected to collect samples of planktonic algae (Microcystis aeruginosa), benthic organisms (Limnodrilus hoffmeisteri) and fish (crucian carp) to analyze their biomarkers and community structure characteristics. Biomarkers and community structure will be analyzed; the monitoring frequency will be set at once a day in the early stage of the pollution event (within 1 month), and adjusted to once every three days in the subsequent stage (1-6 months).

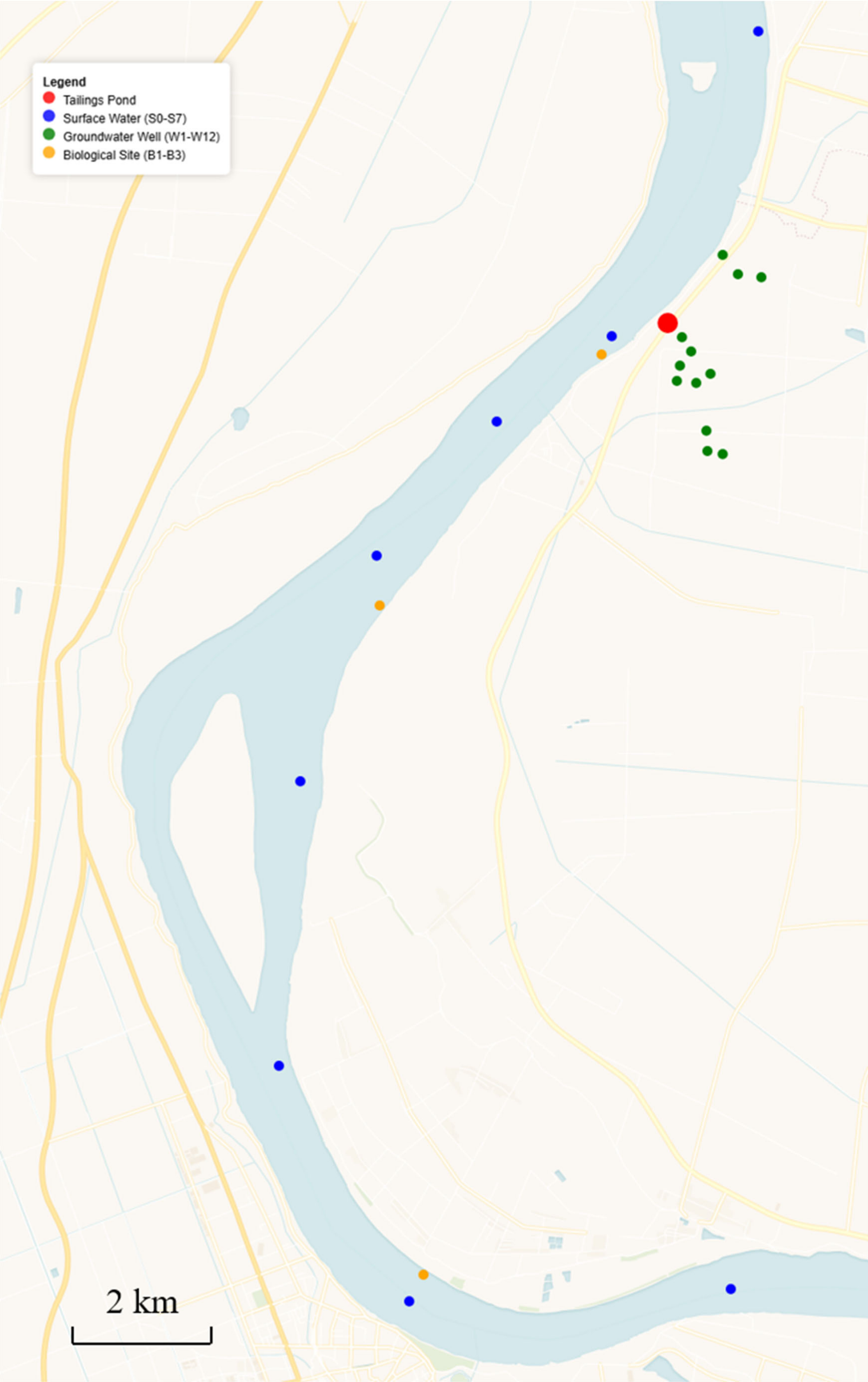

Figure S1 Sampling site layout.

Section S2: Water quality analytical methods and quality control

S2.1 Analytical methods

Table S1. Summary of analytical methods and quality-control procedures adopted for Cr(VI), total As, and arsenic speciation analyses.

Item	Cr(VI)	Total As	As(III)/As(V)
Analytical technique	Ion chromatography (IC)	Atomic absorption spectrometry (AAS)	HPLC-AFS
Target analyte	Dissolved Cr(VI)	Total arsenic	Arsenic species
Sample filtration	0.45 μm membrane	0.45 μm membrane	0.45 μm membrane
Preservation	Refrigerated at 4°C	Acidified and refrigerated	EDTA + ascorbic acid, 4°C
Calibration approach	Multi-point external calibration	Multi-point external calibration	Multi-point external calibration
Working range	Environmentally relevant concentration range	Environmentally relevant concentration range	0–50 μg/L
QA/QC	Blanks, duplicates, calibration verification	Blanks, duplicates, calibration verification	Blanks, duplicates, calibration verification
LOD/LOQ	Method dependent	Method dependent	Method dependent
Supplementary details	Content 2	Content 2	Content 2

S2.2 Quality assurance and quality control

The water body environmental dataset constructed in this study covers three types of indicators comprising surface water, groundwater, and key hydrological parameters. The surface water data is derived from cross-sectional sampling analysis, including pH, dissolved oxygen (DO), total suspended solids (TSS), Cr(VI), As, total nitrogen (TN), and total phosphorus (TP) (units: mg/L); the groundwater data is collected through monitoring wells, covering water level depth (m), hydraulic gradient (dimensionless), and the concentrations of Cr(VI) and As (mg/L); the hydrological parameters integrate

river water level (m), flow velocity (m/s), and flow rate (m^3/s) from hydrological stations, as well as soil permeability coefficient (m/d) and moisture content (%) obtained through laboratory measurements. This dataset provides a multi-dimensional foundation for systematic analysis of regional water environmental quality and pollutant migration.

The data for this study were obtained from on-site sampling and routine monitoring at hydrological stations. Among them, the samples collected on-site were analyzed in the laboratory using ion chromatography to determine anions, and the content of heavy metals was analyzed by atomic absorption spectrophotometry. The hydrological parameters were directly taken from the routine monitoring records of the hydrological stations. In terms of the design of monitoring frequency, it was once a day during the initial stage of pollution (within the first month), adjusted to once every three days during the stable period (from the first to the sixth month), and the hydrological parameters were collected once a month uniformly. All monitoring required that the data integrity be no less than 90% to ensure the reliability and scientificity of the subsequent analysis.

S2.3 Sample preservation procedures

To ensure the accuracy and reliability of the monitoring data, this study systematically implemented quality control measures in both the laboratory and field aspects. During the laboratory analysis stage, for each batch of samples, 3 parallel samples (with a relative deviation controlled within 10%) and 1 blank sample (with a detection limit not higher than 0.001 mg/L) were set up to guarantee the precision and accuracy of the analysis process. In the field sampling process, all sampling containers were pre-treated with acid washing to remove residual heavy metals interference, and strictly in accordance with the relevant requirements of the "Surface Water Environmental Quality Standards" (GB 3838-2002), the water samples were analyzed within 48 hours after collection, thereby effectively maintaining the representativeness and integrity of the samples.

S2.4 Detailed method for arsenic speciation analysis

1. **On-site sampling and preservation**: Water samples were collected in pre-cleaned 50 mL polypropylene centrifuge tubes. Immediately after collection, samples were filtered through 0.45 μm nylon syringe filters. A 20 mL aliquot was transferred to a new tube, and 200 μL of 10% (w/v) ascorbic acid solution and 200 μL of 10% (w/v)

EDTA solution were added to preserve As(III). The samples were sealed with Parafilm, stored in an ice box at 4°C, and transported to the laboratory within 4 hours.

2. **Instrumental conditions**:

1) HPLC system: Agilent 1260 Infinity II
2) AFS detector: Beijing Haiguang AFS-9700
3) Column: Hamilton PRP-X100 anion-exchange column (250 mm × 4.1 mm, 10 μm)
4) Mobile phase: 15 mmol/L $NH_4H_2PO_4$, adjusted to pH 6.0 with ammonia water
5) Flow rate: 1.0 mL/min
6) Injection volume: 100 μL
7) Column temperature: 25°C
8) AFS parameters: Lamp current 60 mA, negative high voltage 300 V, atomizer temperature 200°C, carrier gas flow 300 mL/min, shielding gas flow 800 mL/min

3. **Calibration curve**: Mixed standard solutions of As(III) and As(V) were prepared at concentrations of 0, 1, 5, 10, 20, and 50 μg/L. The calibration curves showed good linearity with correlation coefficients (R^2) > 0.999 for both species.

4. **Quality control**: Procedural blanks were analyzed with every batch of 10 samples. The relative standard deviation of replicate analyses was < 5%, and the recovery rates of spiked samples were 85%-115%.

Table S2 Raw data summary of arsenic speciation at key monitoring points (0-30 days after leakage)

**Monitoring point**	**Time (days)**	**As(III) concentration (μg/L)**	**As(V) concentration (μg/L)**	**Total As concentration (μg/L)**	**As(III)/Total As ratio**
Groundwater well W3 (core pollution area)	1	12.5 ± 0.8	89.7 ± 3.2	102.2 ± 3.5	0.12
	7	42.3 ± 1.5	59.8 ± 2.1	102.1 ± 2.8	0.41
	14	68.9 ± 2.3	31.2 ± 1.2	100.1 ± 2.6	0.69
	30	75.4 ± 2.7	23.5 ± 0.9	98.9 ± 2.8	0.76
Surface water S2 (1 km downstream)	1	8.2 ± 0.5	76.3 ± 2.8	84.5 ± 2.9	0.10

Monitoring point	Time (days)	As(III) concentration (μg/L)	As(V) concentration (μg/L)	Total As concentration (μg/L)	As(III)/Total As ratio
	7	21.5 ± 0.9	52.7 ± 1.9	74.2 ± 2.1	0.29
	14	35.8 ± 1.3	36.4 ± 1.4	72.2 ± 1.9	0.50
	30	41.2 ± 1.5	28.7 ± 1.1	69.9 ± 1.8	0.59

Section S3: Data of biological communities

S3.1 Data content

The biological community data systematically collected in this study include two types of ecological indicator data comprising aquatic organisms and soil organisms. Regarding aquatic organisms, the chlorophyll a concentration of planktonic algae (μg/L), the community composition (represented by the proportion of dominant species), the abundance of benthic organisms Limnodrilus hoffmeisteri (ind/m^2), the avoidance rate (%), and the concentration of metallothionein (MT) in the fish crucian carp (μg/g) and its embryo malformation rate (%), were all measured. For soil organisms, the soil enzyme activities of urease and dehydrogenase ($mg \cdot g^{-1} \cdot d^{-1}$) and the microbial community abundance (represented by Dehalococcoides bacteria, cells/mL) determined by real-time fluorescence quantitative PCR (qPCR) were all obtained from the post-accident public emergency monitoring records. These multi-dimensional biological parameters provide important basis for comprehensive evaluation of regional ecological quality and pollution effects.

S3.2 Data sources and monitoring frequencies

The biological community data used in this study were obtained from the post-accident public emergency monitoring records provided by the local ecological and environmental authority. According to these records, community structure was determined by microscopic counting, enzyme activity was analyzed by spectrophotometry, metallothionein (MT) concentration was quantified by enzyme-linked immunosorbent assay (ELISA), and microbial abundance was detected by quantitative real-time PCR (qPCR). The authors did not directly perform any organism collection, dissection, or laboratory exposure. The monitoring frequency was every two weeks during the pollution stage and once a month after entering the recovery period;

enzyme activity and microbial data were collected once a month. All data were required to have a validity of no less than 85% to ensure the reliability of subsequent ecological evaluation and statistical analysis.

S3.3 Data accuracy control

To ensure the representativeness of the biological community data and the accuracy of the analysis methods, this study implemented a systematic quality control measure. During the sample collection process, three parallel samples were set at each sampling point, and they were mixed evenly before analysis to effectively reflect the overall characteristics of the sampling points and reduce random errors. In terms of the validation of the analysis methods, national standard substances (such as metal sulfoprotein standards) were used to calibrate and verify the detection process, with the requirement that the relative error of the measurement results should not exceed 15%, thereby ensuring the accuracy and reliability of data acquisition.

Section S4: Determination methods of biological indicators

S4.1 The enzyme-linked immunosorbent assay (ELISA) method

The following protocols were those employed by the emergency monitoring laboratories, as documented in the public emergency monitoring records; the authors did not directly perform these assays. Firstly, accurately weigh the gill tissues and homogenize them. Then, extract the total protein by centrifugation. Add the gill tissue protein extract to the microplate that has been coated with anti-MT antibodies. After the MT (antigen) fully binds to the solid-phase antibody, wash away the unbound substances; then add the enzyme-labeled detection antibody to form an "antibody - antigen - enzyme-labeled antibody" complex; wash again, and then add the corresponding enzyme substrate solution for the color reaction. Finally, use the microplate reader to measure the absorbance value of each well at a specific wavelength. The concentration of MT in the samples can be calculated by plotting a standard curve with a known concentration of standard samples simultaneously, thereby obtaining accurate quantitative results.

S4.2 Determination of oxidative stress indicators

Superoxide dismutase (SOD) activity was determined by the xanthine oxidase method. One unit of SOD activity was defined as the amount of enzyme that inhibits the reduction of nitroblue tetrazolium (NBT) by 50% at 550 nm. Catalase (CAT)

activity was measured by the ammonium molybdate method, which quantifies the decomposition of hydrogen peroxide (H_2O_2) by CAT at 405 nm. Malondialdehyde (MDA) content was determined by the thiobarbituric acid (TBA) method, where MDA reacts with TBA to form a red product with a maximum absorption peak at 532 nm.

All assays were performed in strict accordance with the manufacturer's instructions. For each sample, three technical replicates were analyzed, and the results were normalized to the total protein content of the tissue homogenate, which was determined by the bicinchoninic acid (BCA) method. The intra-assay coefficient of variation was controlled below 8%, and the inter-assay coefficient of variation was below 12%.

Section S5: Advection-Diffusion equation

Based on the Advection-Diffusion equation formula (1), key process parameterization and innovative algorithm processing are carried out, and each physical change process is discussed separately.

S5.1 Hydrodynamic dispersion process

The hydrodynamic dispersion theory was first proposed by Bear[1] and the anisotropic dispersion tensor form used in this study is consistent with the description of Zheng and Bennett[2].

In the process of advection and diffusion, hydrodynamic dispersion plays a significant role. By introducing the permeability tensor Kij to determine anisotropic dispersion for innovative processing, the control equation is obtained as shown in formula (S1):

$$\mathbf{D_h} = \begin{pmatrix} \frac{\alpha_L v_x^2}{\parallel \mathbf{v} \parallel} + D_m & \frac{\alpha_T v_x v_y}{\parallel \mathbf{v} \parallel} \\ \frac{\alpha_T v_x v_y}{\parallel \mathbf{v} \parallel} & \frac{\alpha_T v_y^2}{\parallel \mathbf{v} \parallel} + D_m \end{pmatrix} \qquad (S1)$$

In the formula, α_L--Longitudinal dispersion coefficient (m) describes the characteristic length of mechanical dispersion along the flow direction (longitudinal), reflecting the longitudinal extension characteristics of convective dispersion in the medium.

α_T--Lateral dispersion (m) is a characteristic length that depicts the mechanical dispersion in the vertical water flow direction (lateral), reflecting the lateral diffusion

capacity of the medium for convective dispersion.

v_x、v_y--The components of the flow velocity in the x and y directions (m/s) constitute the spatial decomposition of the flow velocity vector v, which is used for calculating anisotropic diffusion.

$\| v \|$--The magnitude of the velocity vector (m/s), which represents the speed of the flow, $\| v \| = \sqrt{v_x^2 + v_y^2 + v_z^2}$ (in a three-dimensional scenario), is used to normalize the velocity components and calculate the diffusion coefficient tensor. When in a two-dimensional scenario, vz = 0 m/s.

D_m--Molecular diffusion coefficient (m^2/s) reflects the diffusion characteristics of solute molecules themselves. It is not driven by convection but is solely caused by molecular thermal motion. It is the fundamental parameter for the diffusion effect in hydrodynamic dispersion.

In the field of seepage mechanics, the flow velocity v is related to Darcy's law and the permeability. The permeability tensor acts as a vector influence, so the direction of the flow velocity v is determined by the permeability tensor Kij, as shown in Equation (S2):

$$\boldsymbol{v} = -\frac{K_{ij}}{\mu}\nabla h \qquad (S2)$$

In the formula, K_{ij}--Permeability tensor (m^2), which describes the anisotropic permeability characteristics of the medium, is represented by the tensor components Kij, which reflect the differences in permeability capabilities in different directions, and is used to determine the anisotropic dispersion characteristics.；

μ——Hydrodynamic viscosity (Pa·s).

∇h——Hydraulic gradient.

S5.2 Surface complexation adsorption

The surface complexation model used in this study is based on the diffuse layer model proposed by Davis et al.[3], which can accurately describe the adsorption behavior of heavy metal ions on mineral surfaces.

$\sum R_{kin}$ represents the total sum of the sink/source terms for the elementary processes of surface complexation adsorption. The surface complexation adsorption process encompasses both physical and chemical processes. Based on physical adsorption, the chemical process involves a significant bonding effect of complexation on metal ions, as defined in formula (S3). The relationship between kf and $\Delta\psi$ is

shown in formula (S4), and the specific surface complexation adsorption model equation is given in formula (S5). Thus, it can be concluded that the Zeta potential is an important regulatory parameter in the surface complexation adsorption process.

$$\Delta\psi \propto \frac{1}{\sqrt{I}} \qquad (S3)$$

$$k_f = k_0 \cdot \exp\left(\frac{-E_a + \alpha\Delta\psi}{RT}\right) \qquad (S4)$$

$$\frac{\partial S}{\partial t} = k_f C(Q_{max} - S) - k_r S e^{-\beta\Delta Eh} \qquad (S5)$$

In the formula, S--Surface adsorption capacity (mg/m2), represents the ratio of the mass of solute adsorbed on the surface of the medium to the surface area, reflecting the degree of surface adsorption load.

t--Response time (s)；

k_f--Adsorption rate constant (L/(mg·d)), describes the rate at which solute is adsorbed onto the surface of the medium, and is affected by factors such as surface potential.

C--Concentration of solute in the solution (mg/L), source of concentration gradient driving the adsorption process.

Q_{max}--Surface maximum adsorption capacity (mg/m^2), characterizes the maximum load that the medium surface can accommodate for solute adsorption, and reflects the total number of adsorption sites.

k_r--Desorption rate constant (d^{-1}), describing the rate at which adsorbed solute molecules desorb from the surface and enter the solution.

β--The coefficient associated with the redox potential （V^{-1}） characterizes the intensity of the influence of the redox potential difference ΔEh on the desorption process. The desorption rate is regulated by the exponential term $e^{-\beta\Delta Eh}$.

ΔEh--Redox potential difference (V) reflects the differences in the oxidation-reduction environment of the system and drives the desorption behaviors related to redox.

I--Ion strength.

$\Delta\psi$--The surface potential difference (V) is significantly regulated by the ionic strength and plays a crucial role in controlling the surface adsorption kinetics.

k_0--Reference rate constant.

E_a——Apparent activation energy.

α——The correction coefficient of potential for adsorption energy.

R——Gas constant（J/(mol·K)）.

T——Temperature（K）.

In an oxidation-reduction reaction system, the oxidation-reduction potential difference ΔEh represents the surface potential difference value in the environmental system. Usually, in the calculation process, ΔEh and $\Delta\psi$ are considered to be the same value. k_f as $\Delta\psi$ changes, the surface potential difference significantly affects the surface adsorption kinetics but has a negligible effect on the desorption rate. $\Delta\psi$ affects k_f through changing the energy barrier (E_a) of the adsorption process, reducing the energy barrier (E_a decreases) and increasing k_f. Therefore, in the surface complexation adsorption process, the effective regulation of the surface potential difference $\Delta\psi$ for controlling the reaction process is a key step to ensure the smooth progress of the reaction, and it is usually determined as the difference between the Zeta potential before and after the reaction. The final value calculated is the result of the $\sum Rkin$ term in the Advection-Diffusion equation model by $\frac{\partial S}{\partial t}$.

S5.3 Kinetic of biodegradation

The microbial degradation kinetic model was constructed based on the Monod equation, and the spatial heterogeneity of microbial abundance was considered according to the method of Prommer et al.[4].

The degradation of pollutants in water bodies, especially heavy metal ions, mainly relies on the removal effect of microorganisms on the pollutants. On this basis, a biodegradation kinetic model is constructed. The spatially heterogeneous degradation parameter λ is established based on the qPCR abundance field Ndhc(x,y) of Dehalococcoides. The governing equation is as shown in Formula (S6):

$$\lambda = \mu_{max}\frac{C}{K_s + C}\frac{[H_2]}{K_{m,H_2} + [H_2]}N_{dhc} \qquad (S6)$$

In the formula, μ_{max}--Maximum degradation rate (s^{-1}), describes the upper limit of microbial degradation activity under optimal conditions, reflecting the degradation potential.

K_s --Saturation constant (mg/L), the concentration of the solute when the degradation rate reaches half of the maximum degradation rate, reflecting the affinity of microorganisms to the substrate.

C--Concentration of solute in the solution (mg/L), source of concentration gradient driving the adsorption process.

$[H_2]$--Hydrogen gas concentration (mol/L), as an electron donor in the biodegradation process, affects the rate of the degradation reaction.

K_{m,H_2}-- Hydrogen partial saturation constant (mol/L), which characterizes the affinity of microorganisms for hydrogen utilization, and is compatible with the hydrogen-driven degradation kinetics.

N_{dhc}--The abundance of Dehalococcoides bacteria (cells/mL), determined by qPCR, was used to construct a spatial distribution field $N_{dhc}(x,y)$ to describe the spatial heterogeneity of microbial degradation.

λ--Microbial degradation rate（s^{-1}）.

S5.4 NAPL phase dissolution

The fluxes that replenish solutes to the water body through processes such as dissolution of non-aqueous phase liquids (NAPL) are the mechanisms of sources and sinks in water pollution. The activity coefficient γ is introduced to calculate the equilibrium concentrations of multiple-component NAPL phases, and the governing equations as shown in formulas (S7) and (S8) are obtained:

$$Q_s = K_{la}(C_s^* - C) \tag{S7}$$

$$C_s^* = \frac{\gamma x P^{sat}}{RT} M \tag{S8}$$

In the formula, K_{la}——Liquid mass transfer coefficient (m/d) describes the mass transfer rate of solute from the NAPL phase to the water phase, reflecting the kinetic characteristics of phase-to-phase mass transfer.

Q_s——Dissolution flux (mg/(m^2·d)), reflects the mass of solute that dissolves from NAPL into the water phase per unit time and per unit area.

C_s^*--NAPL phase equilibrium concentration (mg/L), the concentration of the solute in the aqueous phase when it reaches a dissolution equilibrium with the NAPL phase, subject to corrections such as activity coefficients.

C--The actual solute concentration in the aqueous phase (mg/L), the concentration difference driving force that initiates the dissolution process (C_s^* - C represents the concentration driving force)

γ--Activity coefficient is used to correct the non-ideal behavior of solutes. It is introduced when calculating the equilibrium concentration of multi-component NAPL

phases and reflects the influence of the interaction between ions in the solution on the dissolution equilibrium.

x--The mole fraction of solute in the NAPL phase, describing the composition ratio of the solute in the NAPL phase.

R--Gas constant（J/(mol·K)）.

T--Temperature（K）.

M--Molar mass of solute（g/mol）.

P^{sat}-The saturated vapor pressure (Pa) of the NAPL phase, which is related to the physical and chemical properties of the NAPL phase, is a fundamental thermodynamic parameter for calculating the equilibrium concentration.

S5.5 Kernel parameter

The pollutant diffusion was predicted using the Advection-Diffusion equation model. The core parameters were calibrated based on site monitoring and literature, and the results are shown in Table S3.

TableS3 The core parameter values of the groundwater pollution prediction model

Parameter	Physical significance	Value (Cr(VI) as an example)	Source/Basis
D_h	Comprehensive coefficient of solute diffusion and mechanical dispersion	$0.375\ \mathrm{m}^2/\mathrm{d}$	Calculate by formula (S1)（$\alpha_L = 2.5$ m，$\alpha_T = 0.25$ m）
v	Water flow driving speed	0.15 m/d	Calculate by formula (S2)（$K_{ij} = 1.8 \times 10^{-12}\ \mathrm{m}^2$）
λ	Microbial degradation attenuation coefficient	$1.2 \times 10^{-6}\ \mathrm{s}^{-1}$	Calculate by formula (S6)（$N_{\mathrm{dhc}} = 5.2 \times 10^5$ cells/mL）
$\sum R_{\mathrm{kin}}$	Net rate of surface complexation adsorption	-8.5×10^{-6} mg/(L·s)	Calculate by formula (S5)（$\Delta\psi = -32$ mV）
Q_s	The rate of dissolution and replenishment of organic matter	4.2×10^{-5} mg/(L·s)	Calculate by formula (S7)（$K_{la} = 2.5 \times 10^{-2}$ m/d）

S5.6 Model calibration, validation, and uncertainty analysis

1. **Calibration algorithm**: The SUFI-2 algorithm was used for parameter calibration, which quantifies parameter uncertainty by calculating the posterior distribution of parameters based on observed data. The objective function was defined as the maximization of the Nash-Sutcliffe Efficiency (NSE) coefficient.

2. **Calibration process**: A total of 6 key parameters were calibrated, including longitudinal dispersion coefficient (αL), lateral dispersion coefficient (αT), adsorption rate constant (kf), desorption rate constant (kr), microbial degradation rate constant (λ), and NAPL dissolution mass transfer coefficient (Kla). The parameter ranges were determined based on literature values and site characteristics.

3. **Validation metrics**: The model performance was evaluated using three metrics:

1) Nash-Sutcliffe Efficiency (NSE): NSE > 0.7 indicates good model performance
2) Root Mean Square Error (RMSE): Lower values indicate better model accuracy
3) Coefficient of determination (R^2): $R^2 > 0.6$ indicates acceptable model performance

4. **Uncertainty analysis**: The 95% prediction uncertainty (95PPU) interval was calculated based on the posterior parameter distribution. The P-factor (percentage of observed data within the 95PPU interval) was 0.89, and the R-factor (average width of the 95PPU interval divided by the standard deviation of observed data) was 0.62, indicating that the model had good uncertainty characterization ability.

The detailed calibration results and uncertainty intervals for each parameter are shown in Table S4.

Table S4 Calibrated parameter values and uncertainty ranges

Parameter	Calibrated value	95% confidence interval	Sensitivity rank
αL (m)	2.5	1.8-3.2	1
αT (m)	0.25	0.18-0.32	2
kf (L/(mg·d))	0.012	0.008-0.016	3
kr (d^{-1})	0.002	0.001-0.003	4
λ (s^{-1})	1.2×10^{-6}	0.8×10^{-6}-1.6×10^{-6}	5
Kla (m/d)	0.025	0.018-0.032	6

The parameter sensitivity ranking and key impact scenarios are presented in

Supplementary Material Content 6 and Table S4.

Section S6: Parameter sensitivity verification

Parameter sensitivity analysis is a key method for quantitatively evaluating the degree of influence of model input parameters on the output results. Its core lies in revealing the regulatory effects of each parameter within a reasonable range of variation on the solute transport process (represented by $\frac{\partial(\theta C)}{\partial t}$). Based on Equation (1), through the calculation of partial derivatives combined with the physical variation range of parameters, this section systematically analyzes the sensitivity of the hydrodynamic dispersion tensor (D_h), the biodegradation rate constant (λ), the total sum of surface complexation adsorption kinetics ($\sum R_{kin}$), and the NAPL phase dissolution flux (Q_s). This provides a basis for model parameter optimization and uncertainty analysis.

The sensitivity characteristics of water quality-related parameters and the key influencing scenarios are shown in Table S5.

TableS5 Sensitivity of water quality-related parameters and key impact scenarios

Parameter	Partial derivative expression	Range of variation	Level of influence	Key impact scenarios
D_h	$\nabla\cdot(\theta\nabla C)$	10^{-10}~10^{-6} m^2/s	high	Pollution plume diffusion stage, high-concentration gradient area
λ	$-\theta C$	10^{-8}~$10^{-6}s^{-1}$	medium to high	Microbial active areas, medium and long-term pollution attenuation
$\sum R_{kin}$	1	-10^{-5}~10^{-5} mg/(L·s)	medium	Low-permeability medium, surface adsorption dominant zone
Q_s	1	10^{-6}~10^{-4} mg/(L·s)	medium to low	The initial stage of NAPL leakage and the source area of pollution

The results show that in sandy aquifers, the hydrodynamic dispersion coefficient D_h is the main controlling factor for the diffusion of the pollution plume. In fine clay layers, the sensitivity of surface complexation adsorption ($\sum R_{kin}$) increases to 18, becoming a key parameter for the retention of pollutants. However, it only plays a dominant role in the early stage of pollution and gradually loses its influence in the later

stage due to desorption. The biodegradation rate λ has a significant impact on the long-term migration of As.

Section S7: Hydrological-water quality coupling simulation

The SWAT (Soil and Water Assessment Tool) model was developed by the Agricultural Research Service of the United States Department of Agriculture (USDA-ARS) in the 1990s[5]. It is a semi-distributed hydrological model based on physical mechanisms, which is widely used in watershed hydrological cycle, non-point source pollution and water quality simulation[6]. The version used in this study is SWAT 2012, and the model construction and calibration follow the standard procedures described in the SWAT Theoretical Documentation.

S7.1 Hydrological process simulation

Using the Green-Ampt infiltration equation and the Muskingum routing method for parameter calibration, the average daily flow before the accident was 8.5 m^3/s, the peak flow during the rainstorm period was 32 m^3/s (with a lag of 4.5 hours for the rainfall peak), the runoff depth was 42 mm, the infiltration amount in the loam soil area was 18 mm, the infiltration amount in the sandy soil area was 25 mm, the constant of the river storage capacity $K = 2.5h$, and the weight factor $X = 0.25$.

The calibrated Nash coefficient NSE was 0.78, and the verified coefficient was 0.72. The relative error was 10.5%, which met the requirements for hydrological simulation accuracy. During heavy rainfall events, the surface runoff coefficient increased sharply from 0.28 in the normal water period to 0.62, resulting in a 2.2-fold increase in the flux of surface source pollution erosion.

S7.2 Simulation of Pollutant Migration

For Cr(VI) and As in surface water, the Freundlich adsorption isotherm equation and Stokes sedimentation correction were adopted. The Freundlich adsorption constant for Cr(VI) was $K_f = 35.2L/kg$, the empirical constant was $n = 1.3$, the particle diameter was $d_p = 50\mu m$, the particle density was $\rho_p = 2.65g/cm^3$, and the turbulent correction coefficient was $\gamma_{turb} = 1.2$. For total arsenic (As), $K_f = 42.8L/kg$,$n = 1.1$,$d_p = 40\mu m$,$\rho_p = 2.70g/cm^3$,$\gamma_{turb} = 1.1$.

S7.3 SWAT model calibration and validation

The SWAT model was calibrated and validated using the Sequential Uncertainty

Fitting (SUFI-2) algorithm in the SWAT-CUP software. The calibration period was 2020-2021, and the validation period was 2022-2023. A total of 12 hydrological parameters and 8 water quality parameters were calibrated.

The model performance for flow simulation was excellent, with an NSE of 0.85 in the calibration period and 0.78 in the validation period. For water quality simulation, the model achieved an NSE of 0.75 for dissolved Cr(VI) and 0.68 for particulate As in the validation period, which is consistent with the results presented in the main text.

Parameter sensitivity analysis showed that the curve number (CN2) was the most sensitive parameter for flow simulation, while the partitioning coefficient (Kf) was the most sensitive parameter for heavy metal transport simulation. he detailed calibration results, uncertainty ranges and parameter sensitivity ranking are shown in Table S6.

Table S6 SWAT model calibrated parameter values, uncertainty ranges and sensitivity ranking

**Parameter category**	**Parameter**	**Physical significance**	**Calibrated value**	**95% confidence interval**	**Sensitivity rank**
**Hydrological parameters**	CN2	Curve number (runoff generation)	72	65-79	1
	SOL_K	Saturated hydraulic conductivity (mm/h)	15	10-20	2
	ALPHA_BF	Baseflow alpha factor (days)	0.048	0.03-0.06	3
	GWQMN	Threshold water depth for baseflow (mm)	250	180-320	4
	REVAPMN	Threshold water depth for revap (mm)	100	70-130	5
	ESCO	Soil evaporation compensation factor	0.95	0.85-1.05	6
	SURLAG	Surface runoff lag coefficient (days)	2.5	1.8-3.2	7
	CH_K2	Effective hydraulic	120	80-160	8

**Parameter category**	**Parameter**	**Physical significance**	**Calibrated value**	**95% confidence interval**	**Sensitivity rank**
		conductivity of main channel (mm/h)			
	CH_N2	Manning's n value for main channel	0.035	0.028-0.042	9
	CH_S2	Slope of main channel (m/m)	0.0012	0.0008-0.0016	10
	GW_DELAY	Groundwater delay time (days)	15	10-20	11
	GW_REVAP	Groundwater revap coefficient	0.12	0.08-0.16	12
**Water quality parameters**	Kf_Cr	Freundlich adsorption coefficient for Cr(VI) (L/kg)	35.2	28-42	1
	n_Cr	Freundlich exponent for Cr(VI)	1.3	1.1-1.5	2
	Kf_As	Freundlich adsorption coefficient for As (L/kg)	42.8	34-51	3
	n_As	Freundlich exponent for As	1.1	0.9-1.3	4
	SETTLE_CR	Settling velocity of dissolved Cr(VI) (m/d)	0.002	0.001-0.003	5
	SETTLE_As	Settling velocity of particulate As (m/d)	0.08	0.05-0.11	6
	RESUSP_CR	Resuspension coefficient for Cr(VI) (1/d)	0.001	0.0005-0.0015	7
	RESUSP_As	Resuspension coefficient for As (1/d)	0.03	0.02-0.04	8

S7.4 Extreme Rainstorm Simulation Error Analysis

The SWAT model showed relatively low simulation accuracy for the extreme rainstorm event that occurred 7 days after the accident (NSE = 0.62). The main reasons are: (1) The meteorological data used in this study is daily scale, which cannot capture the short-term intensity changes of rainstorms; (2) The model's sediment resuspension module does not fully consider the impact of sudden flow changes on sediment transport. The relative error of Cr(VI) concentration simulation during the rainstorm period was 22.5%, which is within the acceptable range for emergency assessment.

Section S8: Graph theory

The application of graph theory in hydrological and water quality simulation has developed rapidly in recent years. Cole et al.[7] first proposed a graph-based modeling framework for tracing hydrological pollutant transport in surface waters, which significantly improved the computational efficiency compared with traditional numerical models. This study improves on this framework by introducing a diffusion correction term to make it more suitable for the simulation of sudden point source pollution.

S8.1 Pollution prediction based on graph theory

The river system is abstracted as a directed graph $G=(V,E)$, achieving the discretization mapping of the continuous river space. The node set $V=(v_1,v_2,...,v_n)$ corresponds to the river sections, sorted by flow direction from upstream to downstream, where v_1 is the section where the pollution source is located and v_n is the river outlet section; the directed edge set $E=((v_i,v_j)\|i<j)$ corresponds to the river segments, (v_i,v_j) represents the river segment flowing from the upstream section v_i to the downstream section v_j; the river segment length L_{ij} is the physical length of the river segment from node v_i to v_j (m). The adjacency matrix $A=[a_{ij}]_{n\times n}$ of the river system is defined, quantifying the direct connection relationship between nodes as shown in formula (S9).

Definition of river system adjacency matrix $A = [a_{ij}]_{n\times n}$, quantitative direct links between the relationship between node as shown in formula (S9):

$$a_{ij} = \begin{cases} 1 & \text{If there is a direct river section between } v_i \text{ and } v_j \\ 0 & \text{Or else} \end{cases} \quad (S9)$$

To facilitate numerical calculations, the ordinary differential equations of type k are transformed into matrix form, as shown in Equation (S10):

$$\frac{dC}{dt} = (A \otimes W)C - (D_a \otimes W)C - kIC \tag{S10}$$

In the formula, $C(t)$ --Concentration vector$[C_1(t), C_2(t), \ldots, C_n(t)]^T$, mg/L.

W--A weight matrix containing flow velocity and length, $[\frac{u}{L_{ij}} w_{ij}]_{n\times n}$, s^{-1}.

D_a--Diagonal element $d_{ii} = \sum_{j=1}^{n} a_{ij}$,the number of outgoing edges of node i.

I-- Unit matrix.

A-- Adjacent matrix.

S8.2 Graph theory analysis

From a physical perspective, the graph model, through the "node-edge" structure, completely maps the three core processes of convection, diffusion, and degradation in the convection-diffusion equation into the discrete graph structure, ensuring that the physical meaning is completely consistent with the continuous model.

Firstly, there is the mapping of the convection process. In the continuous model, the convection process is described by $u\frac{\partial C}{\partial x}$. The greater the flow velocity u, the faster the migration rate of pollutants along the flow direction. In the graph model, the convection process is transformed into "directed propagation between nodes", which is achieved by defining the propagation coefficient α_{ij}, as shown in Equation (S11):

$$\alpha_{ij} = \frac{u}{L_{ij}} \cdot w_{ij} \tag{S11}$$

In the formula, α_{ij} represents the transmission coefficient of pollutants from node i to node j (s^{-1}); L_{ij} is the river section length from node i to node j (m); w_{ij} is the weight factor, usually set to 1.From formula (3), it can be seen that the larger the u value and the shorter the L_{ij}, the larger the α_{ij}. This is completely consistent with the physical trend in the continuous model, which states that "the greater the flow velocity, the stronger the convection". Secondly, the mapping of the diffusion process. In the continuous model, the diffusion process is described by $D\frac{\partial^2 C}{\partial x^2}$. The larger the diffusion coefficient D, the more significant the diffusion effect due to the concentration difference. In the graph model, the diffusion process is achieved through the correction of the weight factor w_{ij}: by associating w_{ij} with D (such as $w_{ij}=1+\frac{D}{uL_{ij}}$), the propagation coefficient simultaneously reflects the combined effect of convection and diffusion - the larger D, the larger w_{ij}, and the larger α_{ij}, corresponding to an enhanced diffusion

effect, which is consistent with the diffusion mechanism of the continuous model. Finally, the mapping of the degradation process. In the continuous model, the degradation of pollutants is described by $-kC$: the higher the concentration, the faster the degradation rate. In the graph model, this core term is directly retained, and $-kC_j(t)$ is introduced in the equation of the change in node concentration to ensure that the kinetic laws of the degradation process are exactly the same as those of the continuous model.

From a mathematical perspective, the convection-diffusion equation of the continuous model is a partial differential equation, describing the concentration changes in the two-dimensional continuous domain of "space + time"; the graph model needs to convert it into a system of ordinary differential equations, adapting to the "node discreteness" of the graph structure. First, there is spatial discretization, that is, the mapping from continuous x to discrete nodes. The continuous river length x (range [0, L]) is discretized into n nodes $v_1, v_2, ..., v_n$, and each node corresponds to a fixed spatial position x_i (for example, $x_i=(i-1)\cdot\Delta x$, where Δx is the section spacing); the river segments between nodes (v_i, v_j) correspond to the spatial interval [x_i, x_j]. Secondly, the transformation from partial derivatives to the difference in node concentrations. In the continuous model, the first-order spatial derivative (the core of the convection term) is approximately at the discrete nodes as "the ratio of the concentration difference of adjacent nodes to the river length", as shown in formula (S12):

$$\frac{\partial C(x_j,t)}{\partial x} \approx \frac{C_i(t) - C_j(t)}{L_{ij}} \quad (i < j) \tag{S12}$$

In the equation, $C_i(t)$ represents the concentration (mg/L) of node i at time t on the upstream; $C_j(t)$ represents the concentration (mg/L) of node j at time t on the downstream; L_{ij} represents the topological distance between the two nodes (m).

Substituting the convection term $u\frac{\partial C}{\partial x}$ of the continuous model, we obtain the discretized convection term $u\frac{C_i(t)-C_j(t)}{L_{ij}} = \frac{u}{L_{ij}}C_i(t) - \frac{u}{L_{ij}}C_j(t)$. Due to the instantaneous nature and high concentration of point source pollution, there is no need to consider the time integration of continuous emissions and the superposition effect of background concentration. The result is as shown in formula (S13):

$$\frac{dC_j}{dt} = \sum_{i=1}^{n} \alpha_{ij} C_i(t) - \sum_{k=1}^{n} \alpha_{jk} C_j(t) - kC_j(t) \tag{S13}$$

In the equation, $\frac{dC_j}{dt}$ represents the time rate of change of pollutant concentration at node j ($mg\cdot L^{-1}\cdot s^{-1}$); α_{ij} is the propagation coefficient of the pollutant from node i to node j (s-1); $C_i(t)$ is the pollutant concentration at node i at time t ($mg\cdot L^{-1}$); α_{jk} is the propagation coefficient of the pollutant from node j to node k (s^{-1}); k is the degradation coefficient of the pollutant (s^{-1}). To facilitate numerical calculation, the ordinary differential equation system in equation k is transformed into matrix form.

In response to the sudden characteristics of tailings leakage accidents, this study proposes a rapid assessment model for pollution diffusion based on graph theory. The river pollutant diffusion modeling based on graph theory abstracts the river system as a directed weighted graph (G=(V,E,W)), converting the point-source pollution diffusion problem from the traditional full-stream numerical solution of partial differential equations (complexity $O(N^2)$) into the problem of graph's shortest path traversal (complexity $O(V\log V)$), achieving an exponential improvement in computational efficiency. Due to the discreteness of this method, it can only track the key nodes (such as densely populated areas and water sources) on the pollution propagation tree (T_s), and the computational cost is proportional to the size of the affected area ($O(\|T_s\|)$), rather than the full-stream scale ($O(V)$), avoiding the defect of traditional methods' redundant calculations for the entire river basin. At the same time, this method only requires the river topology structure and the estimation of average flow velocity ($u_{ij}\approx L_{ij}/T_{ij}$), weakening the spatial distribution dependence on high-precision diffusion coefficient D(x) and flow velocity field u(x) in traditional methods (which are often lacking high-resolution data in actual river basins), significantly improving the real-time and practicality of emergency response in sudden pollution events.

Firstly, the target river system is characterized as a directed graph structure G=(V,E), where the node set V consists of 32 monitoring sections arranged along the river flow direction (numbered from upstream to downstream as $v_1\sim v_{32}$), with the leakage point located at v_8 section and the estuary section as v_{32}. The edge set E represents the river segment connection relationships between adjacent sections, and the length of each river segment L_{ij} is calculated using the Floyd–Warshall algorithm. The elements in the adjacency matrix *A* are $a_{ij}=1$ if there is a direct river segment, and $a_{ij}=0$ if there is no direct connection. On this basis, a pollutant migration model is established, where the propagation coefficient $\alpha_{ij}=\frac{u}{L_{ij}}\cdot w_{ij}$, with the water flow velocity

$u=0.6m/s$ and the diffusion effect correction factor $w_{ij}=1.1$, and the result is calculated as $\alpha_{ij}=3.3\times10^{-4}s^{-1}$; the natural degradation coefficient of Cr(VI) is set as $k=2.5\times10^{-6}s^{-1}$. The initial conditions are set as $C_1(0)=85.6mg/L$ (at the leakage section), and the initial concentrations of the other sections are zero.

S8.3 Model validation and error analysis

The graph theory model was validated against independent field monitoring data, using 50 sets of dissolved Cr(VI) concentration observations collected from 8 river sections during the accident period (25 sets from the dry season and 25 sets from the flood season) as the reference benchmark.

The validation results showed that the graph theory model achieved an average relative error of 14.2%, a root mean square error (RMSE) of 0.055 mg/L, and a coefficient of determination (R^2) of 0.87 for all sections. Stratified statistics were conducted for different section types and application scenarios: the average relative error was 12.3% for straight main river sections, and rose to 20.1% at the tributary confluence section; the larger error at the confluence was attributed to the simplified treatment of flow mixing in the graph theory model. For the 72-hour emergency response window (the core application scenario of this model), the average relative error was 13.1%. For high-concentration sections (Cr(VI) > 0.3 mg/L) that are the focus of emergency early warning, the average relative error dropped to 11.6%, showing higher prediction accuracy in the risk-concerned interval. Detailed validation statistics are summarized in Table S7.

Table S7 Validation results of the graph theory model against field monitoring data (dissolved Cr(VI))

Category	Number of monitoring groups	Average relative error	RMSE (mg/L)	R^2
Straight main river sections	38	12.3%	0.053	0.92
Tributary confluence section	12	20.1%	0.062	0.85
72-hour emergency response window	22	13.1%	0.045	0.90
High-concentration interval (>0.3 mg/L)	16	11.6%	0.053	0.91
All sections (total)	50	14.2%	0.055	0.87

To quantify the consistency between the graph theory model and the mature hydrological water quality model, we compared the graph theory outputs with the SWAT simulation results. The inter-model comparison showed an average relative deviation of 10.5%, with a minimum deviation of 3.2% in straight sections and a maximum deviation of 18.2% at the confluence section. This result indicates that the graph theory model maintains a prediction accuracy level close to the SWAT model while greatly improving computational efficiency.

A comparison of the computational efficiency between the graph theory model and the SWAT model is shown in Table S8.

Table S8 Computational efficiency comparison

Model	Average computation time for 7-day prediction	Memory usage
SWAT	2.5 hours	8.2 GB
Graph theory	3.2 minutes	0.5 GB

S8.4 Tributary Confluence Error Analysis

The maximum relative error of the graph theory model validated against field monitoring data (20.1%) occurred at the tributary confluence section (5 km downstream of the leakage point). This is because the model simplifies the flow mixing process at

the confluence and assumes that the pollutants are completely mixed instantaneously. The inter-model comparison with SWAT also shows the same error pattern, with a maximum relative deviation of 18.2% at the confluence section. In practical applications, it is recommended to increase the density of monitoring points at confluence sections to correct the model results.

Section S9: Research subjects of aquatic organisms

Taking the target river and its surrounding water bodies as the research object, selected planktonic algae (Microcystis aeruginosa), benthic organisms (Limnodrilus hoffmeisteri), and fish (carp) as indicator organisms, three monitoring groups were set up (pollution group: 1-3 km downstream of the leakage point; control group: 5 km upstream, unpolluted area; recovery period group: 6 months after the leakage, polluted area), with each group having three repetitions. The concentrations of chlorophyll a in planktonic algae, enzyme activities, and community structure were detected, the avoidance behavior, adsorption capacity, and community abundance of benthic organisms were measured, and the molecular markers (MT, SOD), individual development, and population biomass of fish were analyzed.

This study established a multi-level index system for aquatic biological pollution damage covering three scales: molecular cells, individual physiology, and population-ecosystem. It systematically reveals the toxic effects of pollutants on aquatic ecosystems from the microscopic to the macroscopic level, as shown in Table S9.

The indicator system determined in this study is both sensitive, specific and operational, providing a structured endpoint for damage quantification in the subsequent bio-chemical coupling model.

TableS9 Multi-level indicator system for aquatic organism pollution damage and its application scenarios

Damage level	core index	biological significances	Association of typical pollutants	measuring method
Molecules /Cells	MT concentration	Metal detoxification capacity and exposure dose markers	Cd, Pb, Zn, Cu	ELISA/mass spectrometry method
	SOD/CAT	Intensity of	Heavy metals,	Spectrophotometr

	activity & MDA content	oxidative stress and degree of lipid peroxidation	PAHs, pesticides	y/fluorescence method
	CYP1A enzyme activity / mRNA expression	Metabolic activation markers of organic pollutants	PAHs, PCBs, dioxins	Enzyme activity determination / qPCR
	Lysosomal membrane stability (LMS)	Universal markers of cellular homeostasis imbalance	Multi-pollutant stress	Neutral red retention method
Individual /Physiology	Embryo hatching rate & larval deformity rate	Intensity of developmental toxicity	Heavy metals, endocrine disruptors	Microscopic observation statistics
	Swimming ability (speed, endurance)	Neuromuscular function and energy metabolism status	Organophosphorus pesticides, heavy metals	Behavior tracking system
	Reproductive capacity (egg production, GSI)	Population continuity ability	EDCs, persistent pollutants	Count and Weigh Method
	Benthic organisms' burrowing/avoidance behavior	Sediment toxicity indication	Sediment-bound heavy metals/organic substances	Multi-chamber selective experiment
Population /Community	Shannon Diversity Index (H')	Community structure stability	Comprehensive pollution pressure	Taxonomic identification and calculation
	Benthic organism integrity index (IBI)	The ecological functional integrity of sediments	Sediment pollution	Construct the index based on the parameters of the classification group

Abundance of key functional groups	Energy transfer efficiency of the food web	Nutrients, pesticides	Sampling count of plankton webs
Algal community succession (diatom/cyanobacteria ratio)	Primary productivity structure and degree of eutrophication	N/P nutrients, herbicides	Microscopic identification/pigment analysis

At the molecular cell level, the concentration of metallothionein (MT) serves as a sensitive indicator of metal detoxification ability and exposure dose. It can be detected by ELISA or mass spectrometry and specifically responds to heavy metal pollution such as Cd, Pb, Zn, and Cu. The activities of superoxide dismutase (SOD) and catalase (CAT) and the content of malondialdehyde (MDA) constitute the triad of oxidative stress indicators. These are determined by spectrophotometry or fluorescence methods and are used to assess the degree of lipid peroxidation caused by heavy metals, polycyclic aromatic hydrocarbons, and pesticides. The activity of CYP1A enzyme and its mRNA expression level are quantified by enzyme activity measurement and qPCR technology, which are key molecular markers for characterizing the metabolic activation of organic pollutants such as PAHs, PCBs, and dioxins. The stability of lysosomal membrane (LMS) is measured by the neutral red retention method and serves as a universal indicator of cellular homeostasis imbalance. It has good indicative properties for various complex pollution stressors.

At the individual physiological level, the embryo hatching rate and the juvenile malformation rate can be statistically analyzed through microscopic observation to effectively assess the developmental toxicity caused by heavy metals and endocrine disruptors; the swimming ability (including speed and endurance) is quantified using a behavioral tracking system, sensitively reflecting the inhibition of organophosphorus pesticides and heavy metals on the neuromuscular function and energy metabolism; the reproductive capacity is measured by the egg production volume and gonad development index, evaluated through weighing and counting, and is used to diagnose the impact of endocrine disruptors and persistent pollutants on the population's continuation ability; the cave avoidance behavior of benthic organisms is observed through multi-chamber selection experiments, which is an important behavioral indicator for characterizing the toxicity of bound heavy metals and organic pollutants

in sediments.

At the population and community level, the Shannon diversity index is calculated based on taxonomic identification data and reflects the stability changes in the community structure under the combined pollution pressure; the benthic Index of Biotic Integrity (IBI) is constructed by integrating multiple taxonomic group parameters and is specifically used to assess the impact of sediment pollution on the integrity of ecological functions; the abundance of key functional groups is obtained through plankton net sampling and counting, revealing the interference of nutrients and pesticides on the energy transfer efficiency of the food web; the structure of the algal community is characterized by the ratio of diatoms to cyanobacteria, achieved through microscopic identification or pigment analysis, and can sensitively indicate the degree of eutrophication of water bodies and the evolution of primary productivity structure caused by nutrient and herbicide pollution. This indicator system constructs a multi-endpoint diagnostic path from molecular responses to ecological functions, providing comprehensive and hierarchical technical support for ecological risk assessment in different pollution scenarios.

Section S10: Information on aquatic organisms and environmental factors

At the data collection level, this study integrated public monitoring data from a long-term ecological monitoring network (2018–2023), integrating high-frequency data sets from 12 typical sections in the middle and lower reaches of the Yangtze River. The content covered biological statistical parameters, environmental driving factors, and hydrological characteristics. Biological statistical parameters included phytoplankton density (cells/L), benthic animal taxonomic unit number, fish population biomass (g/m^3), fish MT expression level, algal CYP1A enzyme activity, benthic animal avoidance rate, lysosome membrane stability (LMS), and functional indicators (such as algal chlorophyll a concentration and mollusk lysosome membrane stability). Environmental driving factors included heavy metal (As, Cd, Pb) concentration gradients (μg/L), organic matter (PAHs, pesticides) load, water temperature (°C), pH, dissolved oxygen (DO, mg/L), total nitrogen (TN), total phosphorus (TP), and light intensity. Hydrological characteristics included flow velocity (m/s), turbidity (NTU), water depth (m), and other spatiotemporal dynamic parameters.

Section S11: Typical correlations between aquatic biological indicators and environmental factors

The results of the sensitivity analysis based on 1000 Bootstrap resampling show that the heavy metal concentration (contributing rate 32%), water temperature (18%), and dissolved oxygen (15%) are the three main controlling factors explaining the variation of biological indicators, further analysis reveals that nonlinear interaction terms (such as $C_d \times pH$) can additionally explain 12% of the variance, highlighting the importance of multi-factor synergy. Moreover, through Partial Dependence Plots, the critical concentration of Cd for inhibiting the movement of Daphnia magna is quantified as 0.08 mg/L (95% CI: 0.07–0.09), while the threshold of TN for a dramatic change in the algal community structure is 1.2 mg/L (the point of a sudden drop in Shannon index H').

The above correlation analysis not only reveals the driving mechanism of biological damage under environmental gradients, but also provides crucial input for the subsequent construction of the "biological-chemical coupled model". Specifically, it quantifies the functional form of the pollutant dose-biological response (such as linear, exponential or saturation curves) and the corresponding ecological thresholds; clarifies the dominant environmental factors and identifies their regulatory coefficients (such as the Q_{10} value of temperature on toxicity intensity); and determines the key interaction combinations that can lead to damage amplification (such as the synergistic effect of eutrophication and organic pollution).

The typical correlations between aquatic biological indicators and environmental factors, as well as the regulatory mechanisms, are shown in Table S10:

TableS10 Typical correlations and regulation mechanisms between aquatic biological indicators and environmental factors

Biological indicator	Dominant environmental factors	Statistical relation	Regulatory mechanism	Ecological threshold
Expression level of MT in fish	Cd^{2+} concentration, water temperature	Exponential growth (R^2=0.92)	Demand for metal detoxification increases, the metabolic rate accelerates	Cd>0.05 mg/L significant induction occurs
Algal CYP1A enzyme activity	Concentration of benzo [a] pyrene, light intensity	Linear positive correlation (r=0.78)	Photoactivation enhances the metabolic efficiency of enzymes	light exposure > 500 $\mu mol \cdot m^{-2} \cdot s^{-1}$ activation occurs
Benthic organism avoidance rate	Sediment As, pH	Logistic growth (EC_{50}=35 mg/kg)	Acidification promotes the desorption, bioavailability of As	pH < 6.5 threshold decreases by 30%
Phytoplankton density	TN/TP, water temperature	Bimodal distribution (nutritional restriction conversion)	Nutrient competition and temperature-dependent growth	TP>0.08 mg/L triggers cyanobacteria dominance
Lysosomal membrane stability	PAHs concentration, dissolved oxygen	negative correlation (r=-0.65)	Hypoxia inhibits the cell's repair ability	DO<3 mg/L the damage is magnified by 1.8 times

Section S12: The determination results of oxidative stress indicators in the liver tissue of crucian carp

According to the emergency monitoring records, the activities of superoxide dismutase (SOD) and catalase (CAT) and the content of malondialdehyde (MDA) in

the liver tissues of crucian carp were determined by spectrophotometry. The content of MDA continuously accumulated: the MDA content in the pollution group reached 8.5 nmol/mg prot, which was 1.7 times higher than that in the control group (3.1 nmol/mg prot), indicating significant lipid peroxidation damage. The results are shown in Table S10. The previously mentioned 6.2-fold MT induction refers to the framework's standardized Cd exposure module, whereas the oxidative stress indicators (SOD, CAT, MDA) detailed in Table S11 reflect the overarching toxicological responses evaluated under the framework.

TableS11 The determination results of oxidative stress indicators in the liver tissue of crucian carp

Index	Control group	Pollution group（30 day）	Recovery period group（6 month）
SOD activity（U/mg prot）	85.2±6.3	72.5±5.8	81.3±6.1
CAT activity（U/mg prot）	28.6±3.2	21.8±2.5	26.5±2.8
MDA content（nmol/mg prot）	3.1±0.4	8.5±0.7	5.2±0.5

The stability of lysosomal membranes in benthic mollusks dropped to 52% seven days after the incident (90% in the control group), indicating a disruption of cellular homeostasis.

Section S13: Analysis of individual/physiological level

As a dominant fish species in freshwater ecosystems, crucian carp is particularly sensitive to heavy metal pollution during its early developmental stage. Experimental results show that when the concentration of Cd in the water reaches 0.09 mg/L, the embryonic hatching process of crucian carp is significantly inhibited: the hatching rate drops sharply from 95% in the control group to 62%, and the deformity rate rises sharply to 28%, with the main characteristics of the deformed individuals being spinal curvature and pericardial edema. The former directly affects the motor coordination of the young fish, while the latter may lead to respiratory and circulatory dysfunction, further reducing the survival probability.

A 30-day continuous monitoring of the post-hatching juvenile fish revealed that the body length of the fish exposed to Cd was shortened by 35% compared to the control group. This phenomenon is consistent with the developmental toxicity characteristics of heavy metals, which interfere with cell division and inhibit the absorption of nutrients. To assess the impairment of motor ability, the study used a behavior tracking system to measure the swimming speed and endurance of the carp juveniles: their swimming speed decreased from 0.85 m/s in the control group to 0.42 m/s, and the endurance time reduced from 120 minutes to 45 minutes, with both decreases exceeding 50%. This directly reflects the damage to the neural-muscular signal conduction and energy metabolism systems caused by Cd exposure in fish, and the decline in motor ability will significantly increase the risk of predation for the juvenile fish, threatening the continuation of the population.

Limnodrilus hoffmeisteri as a key organism in the benthic ecosystem, its behavioral changes can quickly indicate the degree of sediment pollution. In this study, the avoidance behavior of the water silk worm towards As-contaminated sediments was determined through multi-chamber selection experiments. The results showed that when the As concentration in the sediment exceeded 20 mg/kg, the avoidance rate of Limnodrilus hoffmeisteri reached 65%, while the avoidance rate of the control group was less than 5%; and the avoidance rate showed a typical logistic growth relationship with the As concentration. At low As exposure levels, the avoidance behavior was not obvious, but once it exceeded the threshold of 20 mg/kg, the avoidance rate rose rapidly and gradually stabilized.

Meanwhile, As pollution has also altered the habitat depth of Limnodrilus hoffmeisteri. Its normal burrowing depth is 12 cm, but under high concentration As stress, it shrinks to 5 cm. The core purpose of this behavioral adjustment is to reduce the vertical habitat distance and minimize contact with deep and highly concentrated As-contaminated sediments. This is a defensive strategy for the organism's adaptation in a polluted environment. However, long-term shallow habitation may increase the probability of being captured by upper-level predators, thereby disrupting the benthic food chain structure.

The medaka is often used as a model organism to assess the reproductive toxicity of pollutants due to its short reproductive cycle and easily observable reproductive characteristics. Experimental results show that when the Cd concentration in the water body is 0.05 mg/L, the reproductive function of medaka undergoes significant decline:

the frequency of spawning drops from 3 times/week in the control group to 1.8 times/week, the egg survival rate decreases from 85% to 52%, and more importantly, the Gonadosomatic Index (GSI) drops from 6.2 to 3.8. This series of changes indicate that Cd can accumulate in the gonad tissue of medaka through water exposure. On the one hand, it damages the reproductive cell structure; on the other hand, it interferes with the synthesis and regulation of sex hormones, resulting in reduced gamete production and decreased fertilization success rate. Moreover, continuous observation after the experiment reveals that even when medaka are transferred to clean water, their reproductive function does not recover, confirming that the damage of Cd to the reproductive system of medaka is irreversible. Long-term exposure will directly lead to a decrease in the population size and even local extinction of the species.

Section S14: Construction content of IBI

During the construction of the IBI, the core biological parameters that respond sensitively to pollution were first selected. Then, the original values of each parameter were uniformly converted into values between 0 and 1 using the ratio method or the quantile method. Finally, the scores of each parameter were summed up to obtain the total IBI value, which has a theoretical range of 0–100. A higher score indicates better biological integrity. Based on the IBI construction method, the IBI of the polluted group was calculated to be 28, while that of the control group was 75, and they were classified in the "extreme" category (IBI < 30). At the same time, the IBI was strongly negatively correlated with the concentration of Cr(VI) in the sediment ($r = -0.85$, $p < 0.001$), and could be used as a comprehensive response indicator for sediment pollution.

Section S15: Toxicokinetic-toxicity kinetics (TK-TD) coupling framework

In the context of the interaction between organisms and the environment, the essence of trans-biologic accumulation lies in the dynamic coupling of environmental pollutants and biological systems across multiple interfaces and processes. The core logic stems from the hierarchical correlation of "environmental input - biological response - in vivo accumulation". After pollutants enter the organism from environmental media such as water and sediments, they must undergo a continuous process of "environment-biologic interface contact → physical barrier penetration → biochemical barrier regulation → in vivo distribution and enrichment": Firstly, through

organs such as the skin, gills, and intestines that directly interface with the environment, a material exchange channel is established; then, through passive diffusion and active transport mechanisms of biological membranes, the physical barrier is broken through; subsequently, through enzymatic reactions and cellular metabolism, the transformation or retention is completed; finally, accumulation forms in target organs (such as the liver and muscles).

During this process, the interaction between environmental exposure intensity (pollutant concentration, exposure duration) and biological barrier characteristics (permeability efficiency, metabolic capacity) is the core driving force: high concentration environmental exposure may accelerate the material input into the physical barrier, while the biological organisms can reverse this by up-regulating the activity of metabolic enzymes and enhancing excretion; conversely, low concentration long-term exposure may induce adaptive changes in the biological barrier (such as up-regulation of transporter protein expression), forming a "exposure-adaptation-cumulation" dynamic balance. To quantify this complex coupling relationship, a toxicokinetic-toxicodynamic (TK-TD) coupling framework was constructed, integrating the external driving force of environmental exposure and the internal regulation of the biological barrier into a unified mathematical system. By analyzing the absorption, metabolism, and excretion of pollutants in the organism, the theoretical connection of "environmental input - internal accumulation" was achieved.

The TK-TD framework focuses on the dynamic changes of pollutant concentrations at the target sites within the body. It integrates the three key processes of absorption, excretion, and metabolism through differential equations. The mathematical form (S14) of this framework is constructed in accordance with the "Law of Conservation of Matter" and the "Biological Process Dynamics Laws":

$$\frac{dC_b}{dt} = k_u \cdot C_w \cdot SA - k_e \cdot C_b - k_m \cdot \frac{dE}{dt} \cdot V_{max} \cdot \frac{C_b}{K_m + C_b} \qquad (S14)$$

In the formula, $\frac{\mathrm{dC_b}}{\mathrm{dt}}$--The rate of change of pollutant concentration (denoted as C_b) in specific parts of the organism (such as blood and related parts) over time t reflects the dynamic changes in concentration of the pollutant under the combined effects of absorption, distribution, metabolism and excretion processes in the organism. A positive value indicates that accumulation increases the concentration in that part, while a negative value indicates that excretion or other processes reduce the concentration.

k_u--Gill/epidermal absorption rate constant, used to quantify the rate at which pollutants are absorbed from the environmental medium into the organism (such as a carp) through the gills or epidermis within a specific period of time under specific environmental conditions. The larger the value, the faster the absorption process.

C_w--The concentration of pollutants in the environment (such as water bodies) represents the "material source" concentration for organisms to absorb pollutants from the environment. The magnitude of this value will affect the driving force for organisms to absorb pollutants and the final accumulation level within their bodies.

SA--The specific surface area factor, for organisms such as fish, reflects the effective surface area of a unit mass of the organism in contact with the environmental medium, and is used to measure the interface scale for the absorption of pollutants by the organisms. The SA of fish is $1.2 - 2.5\ \mathrm{cm^2/g}$.

k_e--The rate constant for the excretion of pollutants in an organism (or the combined process of excretion, metabolism, etc.) represents the rate at which pollutants are expelled from a specific part of the organism (corresponding to the location indicated by C_b) within a unit of time. This rate is influenced by both the excretion process and absorption processes, and together they affect the dynamic concentration of pollutants within the organism.

C_b--The concentration of pollutants in specific parts of the body (such as blood, which is a key area related to the distribution, metabolism, etc. of pollutants within the body) is a crucial indicator reflecting the "internal level" of pollutants in the body. Its changes are influenced by the combined effects of absorption, excretion, and metabolism processes.

k_m--Characterizes the coefficient of CYP450-mediated metabolic activation efficiency, which is used to quantify the efficiency characteristics of the pollutant metabolic activation process when CYP450 enzymes are involved, and affects the rate and extent of pollutant metabolism and transformation in the body.

$\frac{dE}{dt}$--The rate of change of the coefficient E related to metabolism reflects the changes in the state of the metabolic process (such as changes in metabolic enzyme activity, activation degree of metabolic pathways, etc.) over time, and indirectly affects the dynamic process of pollutant metabolism and transformation.;

V_{max}--The maximum metabolic rate of this pollutant by the organism represents the maximum capacity of the organism's metabolic system to handle this pollutant. That

is, when the metabolic substrate (the pollutant) is abundant, the maximum amount of pollutant that can be metabolized and transformed per unit time.

K_m--Michaelis constant ,the constant reflects the affinity of metabolic enzymes (such as those involved in the metabolism of pollutants) for pollutants (substrates). When the pollutant concentration $C_b = K_m$, the metabolic rate reaches $V_{max}/2$, and it is used to characterize the characteristics of the enzyme-substrate interaction during the metabolic process.

$k_u \cdot C_w \cdot SA$ is an absorption term that characterizes the material input mechanism of the physical barrier. Here, the value of k_u is determined jointly by the biological membrane structure (such as the thickness and pore size of the phospholipid bilayer) and the physical and chemical properties of the pollutants (such as molecular polarity and lipophilicity). Lipophilic pollutants (with a high K_{ow} value) can easily enter the body through passive diffusion across the gill epithelial cells, corresponding to a higher k_u; while polar pollutants require the active transport through membrane transport proteins, and k_u is positively correlated with the expression level of the transport proteins. The typical k_u value of fish gills (carp gills $k_u = 2.3 \times 10^{-3}$ L$\cdot$ g$^{-1} \cdot$ h^{-1}) is derived based on the microscopic structure of the gill filaments and the diffusion coefficient of the pollutants, reflecting the quantitative correlation of "structure - function".

$k_e \cdot C_b$ is the excretion component, regulating the material output from the biological barrier. Among them, k_e represents the clearance capacity of the biological excretion system (kidneys, gills, intestines), and its theoretical basis is "carrier-mediated active transport kinetics" - the transport proteins on the epithelial cells of the renal tubules can specifically bind to pollutants and pump them into the urine for excretion; while the gills use capillaries on the gill lamellae to form a concentration difference with the water body, promoting the passive diffusion of pollutants to the environment. Therefore, the value of k_e is directly related to the functional state of the excretion organs, reflecting the regulatory effect of "active clearance" of the organisms.

$k_m \cdot \frac{dE}{dt} \cdot \frac{V_{max} \cdot C_b}{K_m + C_b}$ is a metabolic transformation term, analyzing the transformation mechanism of the biochemical barrier. Among them, $\frac{V_{max} \cdot C_b}{K_m + C_b}$ is the Michaelis-Menten term, based on the "enzyme-substrate reaction mechanism", describing the variation law of metabolic rate with the concentration of internal pollutants. When $C_b \ll K_m$, the

metabolic rate is proportional to C_b (first-order reaction); when $C_b \gg K_m$, the enzyme activity is saturated, and the metabolic rate approaches V_{max} (zero-order reaction). Among them, V_{max} represents the total amount of the enzyme and its upper limit of activity, and K_m reflects the affinity of the enzyme to the pollutant. Both are determined by the characteristics of the biological own enzyme system (the expression level and catalytic efficiency of CYP450 enzymes), and are the core parameters for quantifying the function of the biochemical barrier.

By splitting and quantifying the independent processes such as absorption, excretion, and metabolism, the contributions of each process to the accumulation within the body can be separately determined. For instance, by comparing the magnitudes of the environmental absorption term and the in-body excretion term, one can determine whether the pollutant is in a "net accumulation" (absorption > excretion) or "net clearance" (excretion > absorption) state; combined with the changes in the metabolic term, one can further analyze whether the metabolic process intensifies (activates) or alleviates (detoxifies) the accumulation, achieving precise positioning of the "process - accumulation" correlation.

In summary, the cross-barrier accumulation model under the interaction of organisms and the environment has constructed a theoretical system of "environmental input - biological barrier - in-body accumulation" through the TK-TD framework. Based on fundamental scientific laws such as material conservation and enzymatic reaction kinetics, it quantifies the interaction mechanism between environmental driving and biological regulation; through parameter adjustment, it realizes the universal expansion across species, media, and pollutants; from the theoretical perspective, it reveals the intrinsic laws of cross-barrier accumulation under the interaction of organisms and the environment, laying a model foundation for the study of pollutant ecological effects.

Section S16: Comprehensive assessment

This section describes the comprehensive assessment methodology of the proposed framework. The framework is designed for general applicability and includes calculation modules for multiple pollutant types. Representative pollutants (Cd for heavy metals, PAHs for organic pollutants) are used to demonstrate the formula derivation and calculation process. When applying the framework to specific cases, parameters and formulas should be adjusted according to the actual target pollutants.

S16.1 Assessment of damage to ecosystem service functions

The assessment method of ecosystem service function damage is based on the equivalent factor method proposed by Costanza et al.[8] and the biological endpoint response method recommended by the European Commission[9].

The assessment of damage to the ecosystem services of water bodies requires the construction of a multi-dimensional and multi-scale quantitative framework, deeply integrating the ecological toxicological mechanism and the principles of environmental economics. Based on the biological-chemical coupling model (BCIM) output of biological damage parameters (degradation rate of benthic organism abundance, excess multiple of fish metal sulfoprotein expression, degradation rate of algal community Shannon index), this study establishes a complete chain assessment system of "biological endpoint response - service function discounting - economic loss conversion" by integrating the equivalent factor method, energy analysis, and willingness value assessment method. The empirical study of a chemical leakage incident in the Yangtze River Estuary shows that heavy metal pollution led to a 72% decrease in the abundance of the key benthic species Limnodrilus hoffmeisteri. Through the trophic energy flow model, it was discovered that the biomass loss of secondary consumers presented a nonlinear decay characteristic, and its dynamic equation can be expressed as formula (S15):

$$\Delta E_{\text{fish}} = \eta \cdot \frac{\Delta B_{\text{benthos}}}{TLE} \cdot \rho_{\text{conversion}} \cdot \exp\left(-\frac{t}{\tau}\right) \qquad (S15)$$

In the formula, η--The transfer efficiency of energy and matter between different trophic levels reflects the proportion of energy and matter transfer between these levels. The measured average value is 0.12.

TLE--Thermodynamic loss coefficient,1.8.

$\rho_{\text{conversion}}$--Biomass - economic quantity conversion factor， 1200yuan/t；

T--The time decay constant, a characteristic constant reflecting the decay of biomass loss and other factors over time, is valued at 8.3 months.

ΔE_{fish}--The economic loss of secondary consumers (such as fish).

$\Delta B_{\text{benthos}}$--The biomass changes of benthic organisms (such as the Limnodrilus hoffmeisteri) (here it refers to the decrease in biomass).

t--The time variable represents the time elapsed after pollution occurs.

The pollution incident led to a reduction of up to tens of thousands of tons in the

annual fishery output, with direct economic losses exceeding hundreds of millions of yuan. This damage to the resource supply function is not only reflected in the decline of biomass, but also manifested as the simplification of the food web structure leading to a decrease in ecosystem resilience. The recovery period is prolonged by more than 40% compared to the simple recovery of biomass.

In the dimension of ecosystem regulation services, the weakening of the self-organizing ability of water bodies by pollutants exhibits a multi-dimensional, multi-path, and complex time-space dynamic characteristic mechanism. From the microscopic perspective of microbial processes, hexavalent chromium [Cr(VI)] and arsenic [As(III)] interfere with the structure and function of the water body's microbial community in various ways. On one hand, Cr(VI) (as a strong oxidant) and As(III) bind to functional proteins (such as key enzymes involved in ammonia nitrogen metabolism, including ammonia monooxygenase and nitrite oxidoreductase) within microbial cells, changing the spatial conformation of the proteins and thereby inhibiting their biological activity; on the other hand, the stress of Cr(VI)/As triggers the reconfiguration of the ecological niches of the microbial community. The dominant nitrifying bacterial community (ammonia-oxidizing bacteria and nitrite-oxidizing bacteria) gradually declines due to its high sensitivity to Cr(VI), while some pollutant-resistant miscellaneous bacteria can survive but cannot effectively participate in ammonia nitrogen degradation and other self-purification processes. This leads to a reduction of the ammonia nitrogen degradation rate constant as quantified by the Cr(VI)-specific Hill-type dose-response model (Eq. S16), which is actually a comprehensive manifestation of the decline in microbial community functions. This microbial function degradation is not an isolated event but rather like a domino effect, triggering a systematic attenuation of the water body's self-purification ability from the microbial metabolic process to the entire water ecosystem's material circulation and energy flow.

In order to break through the limitations of the traditional description of the interaction between pollutants and the self-purification system of water bodies, and to more accurately and comprehensively depict this complex process, this study integrates theories from multiple disciplines such as hydrodynamics, pollutant migration and transformation kinetics, and microbial ecological kinetics, and constructs a coupled model of river pollutant migration and transformation. This model not only considers the distribution and migration process of pollutants between the aqueous phase and the sediment phase, but also introduces biological processes such as the expression of

functional genes of microbial communities, population competition and cooperation, achieving multi-scale coupling simulation from the macroscopic hydrological process to the microscopic biological metabolism.

Through in-depth analysis of the model output results, it was found that the dose-effect relationship between the self-purification function loss and the Cr(VI) concentration of pollutants is expressed as formulas (S16) and (S17) as follows:

$$\Delta k_{nit} = V_{max,Cr} \cdot \frac{[Cr(VI)]^n}{{K_{m,Cr}}^n + [Cr(VI)]^n} \cdot (1 - e^{-\gamma t}) \quad (S16)$$

$$L_{self} = \Delta k_{nit} \times V \times C_{vol} \quad (S17)$$

In the formula, Δk_{nit}--The variation (reduction) in the rate constant of ammonia nitrogen degradation (d^{-1}).

$V_{max,Cr}$--Maximum inhibition rate of the nitrification rate constant by Cr(VI) (d^{-1}).

$[Cr(VI)]$--The concentration of the pollutant Cr(VI) (mg/L).

n--Hill coefficient, 1.7.

$K_{m,Cr}$--Half-effect concentration for Cr(VI) inhibition of nitrification (mg/L).

γ--The coefficient related to the development of inhibition over time (d^{-1}).

t--The time elapsed after pollution occurs (d).

C_{vol}--The unit cost of artificial water purification (CNY/m^3).

V--Volume of the contaminated river reach (m^3).

Δk_{nit} represents the change in the ammonia nitrogen degradation rate constant, which directly reflects the degree of damage to the core function of water body self-purification (ammonia nitrogen degradation); $V_{max,Cr}$ represents the maximum inhibition rate of the nitrification rate constant by Cr(VI), and this parameter is determined based on indoor simulation experiments and field in situ monitoring reported in the literature, reflecting the potential upper limit of the inhibitory effect of Cr(VI) on the ammonia nitrogen degradation process under extreme pollution conditions, and also indirectly reflecting the maximum tolerance threshold of the water body microbial community to Cr(VI) stress; n is the Hill coefficient, which is used to describe the intensity of the synergistic or antagonistic effect between pollutant concentration and the loss of self-purification function. When $n > 1$, it indicates the existence of a positive synergistic effect; $K_{m,Cr}$ is the half-effect concentration, which is the Cr(VI) concentration corresponding to half of the maximum inhibition rate of

self-purification function, and it is a key indicator for measuring the ecological toxicity of Cr(VI) to nitrifying microorganisms; γ is a coefficient related to the development of inhibition over time, reflecting the dynamic characteristics of the degradation of self-purification ability; t is the time elapsed after the pollution occurred (in days), and the introduction of the time dimension enables this model to simulate the dynamic evolution process of the water body's self-purification function after the pollution event. In formula (S17), V is the volume of the contaminated river reach (m^3), and C_{vol} is the unit cost of artificial water purification (CNY/m^3). The dimensional consistency is verified as follows: Δk_{nit} (d^{-1}) × V (m^3) yields the effective volume of self-purification service lost per day (m^3/d), and multiplying by C_{vol} (CNY/m^3) yields CNY/d, which is dimensionally complete.

In the field of ecosystem service research, the relationship between the degree of damage and the time dimension is a highly complex and dynamic core issue. Analyzing the damage to ecosystem services from the perspective of the time dimension requires the construction of an analytical framework that integrates the dynamic evolution of ecological processes and the temporal attributes of economic value. The ecosystem itself has natural succession and recovery patterns over time, which means that the damage to services is not static but will decline or change over time. At the same time, the time value of economic funds requires the discounting of the loss of ecosystem services at different time points to achieve comparable analysis of inter-temporal values. To accurately depict this relationship in the time dimension, the intertemporal loss model is introduced, and its expression is (S18):

$$D_t = \sum_{t=0}^{T} \frac{L_0 \cdot e^{-\lambda t}}{(1+r)^t} \qquad (S18)$$

In the formula, D_t--Cumulative discounted loss, after taking into account the time value, the total present value of the damage to ecosystem services over a certain period of time.

T--The time span (total number of periods) represents the time range of the assessment.

L_0--Initial annual loss.

λ--attenuation coefficient.

r--social rate of discount.

e--natural constant.

Cumulative discounted loss D_t represents the total present value of the ecosystem service damage over a specific period, considering the time value. The core significance of this indicator lies in converting the process of ecosystem service damage, which has distinct temporal dynamic characteristics, into a value that can be measured and compared within the same time dimension through economic discounting, thereby providing a unified value scale for the quantitative assessment of ecological damage. T is the time span (total number of periods), which defines the time range for the assessment, and its value needs to be comprehensively considered based on factors such as the recovery period of the ecosystem, the duration of pollution impact, and the actual needs of the assessment. Different ecosystem types (such as forest ecosystems, aquatic ecosystems, etc.) and different types of pollution stress will correspond to different reasonable time spans, which also makes T one of the key parameters in the model that reflects the specificity of the ecosystem. L_0 is the initial annual loss, that is, the annual ecosystem service loss amount in the initial stage of pollution, and it is the basis for the calculation of the entire inter-period loss. Its determination depends on the precise assessment of the damaged status of ecosystem services in the initial stage of pollution, involving the classification accounting of ecosystem services (such as provisioning services, regulating services, cultural services, etc.) and the quantitative analysis of the degree of damage of each service type. The attenuation coefficient λ reflects the rate at which ecosystem service losses naturally decline over time, reflecting the recovery or damage evolution characteristics of the ecosystem. When the ecosystem has a strong self-repair ability, the λ value is larger, meaning that the loss will decay rapidly over time; conversely, if the ecosystem's self-repair ability is weak, the λ value is smaller, and the decay of the loss is slower. r is the social discount rate, used to discount future loss amounts to the current time, reflecting the time value of funds. The selection of the social discount rate usually needs to consider factors such as society's time preference and the opportunity cost of capital. Different discount rates will have a significant impact on the cumulative discounted loss D_t. A high discount rate will reduce the present value of future losses, while a low discount rate will increase the present value of future losses. e is the natural constant, $e^{-\lambda t}$ which depicts the decay process of losses over time and works together with λ to describe the dynamic change trajectory of losses in the time dimension from the perspective of the ecological process.

Ecological damage is often the result of the combined effect of multiple factors

over different time periods. Through this model, the losses in each time period can be accurately divided and measured. Combined with data such as pollution fluxes, it can clearly determine the contribution ratio of different responsible entities in the time dimension, providing a scientific basis for the determination of responsibility in judicial decisions. On the other hand, based on the recovery cycle characteristics of ecosystem services, the allocation of restoration resources can be optimized. Different ecological restoration measures will have different impacts on the recovery cycle of ecosystem services. By using this model, the time dynamic changes in the loss of ecosystem services under different restoration strategies can be simulated, and thus the optimal solution for the allocation of restoration funds can be found.

S16.2 Environmental restoration/environmental governance cost assessment

The cost calculation methods for in-situ chemical reduction (ISCR) and permeable reactive barrier (PRB) technologies refer to the US EPA Remediation Technology Cost Manual[10].

The assessment of environmental restoration and environmental governance costs for water body pollution requires the establishment of a complete cost quantification framework that comprehensively considers the technical feasibility of the engineering implementation, the temporal dynamics, and the response characteristics of the ecosystem. Based on the output of the integrated assessment model of water bodies and aquatic organisms, this study integrates the principles of engineering economics, the efficiency model of restoration technologies, and the dynamics of ecosystem recovery, and establishes an "pollution characteristics - technology selection - cost optimization" tripartite assessment system. Taking the restoration project of a composite polluted site in the Yangtze River Delta as an example, the co-pollution of heavy metals and organic substances led to a peak concentration of hexavalent chromium in groundwater reaching 18.7 mg/L (374 times over the limit of GB 5749), and the accumulation of polycyclic aromatic hydrocarbons in the sediment was > 35 mg/kg. Through technical comparison, the combined repair scheme of in-situ chemical reduction (ISCR) and permeable reactive barrier (PRB) was determined.

The direct cost of the restoration project is jointly determined by technical efficiency and pollution flux. For groundwater hexavalent chromium pollution, in the ISCR technology, the dosage of sodium dithionite added is non-linearly related to the

volume of the pollutant, as expressed by formula (S19):

$$m_{\text{reductant}} = \alpha \cdot V_{\text{contam}} \cdot C_{\text{Cr}} \cdot \left(1 + e^{-\beta \cdot K_h}\right) \qquad (S19)$$

In the formula, $m_{\text{reductant}}$ --The dosage of the reducing agent (sodium hyposulfite).

α--Reagent reaction efficiency coefficient.

V_{contam}--The volume of pollutants represents the volume of groundwater and other pollutants contaminated by hexavalent chromium.

C_{Cr}--The concentration of hexavalent chromium pollutants.

β--Reaction kinetics attenuation coefficient.

K_h--Hydraulic conductivity coefficient.

α reflects the ratio of the actual reaction quantity of the agent to the theoretical stoichiometric reaction quantity. This parameter needs to be calibrated through laboratory batch experiments and site pilot tests. β reflects the time-space characteristics of the attenuation of the remediation reaction activity. The heterogeneity of the contaminated site will cause the activity of the remediation agent to continuously decay along the transport path, and it is necessary to compensate for the attenuation loss by increasing the dosage of the agent or optimizing the dosing method. The former directly increases the material cost, while the latter increases the equipment operation and maintenance cost and process control cost, ultimately resulting in an increase in direct costs. K_h describes the permeation-diffusion ability of the remediation medium in the subsurface medium. If the medium transfer efficiency is low, the remediation agent is difficult to quickly reach the contaminated core area, and it is necessary to ensure the remediation effect by extending the remediation time (increasing equipment operation and monitoring costs), densifying the distribution points/ injection wells (increasing construction and hardware costs), etc., which directly pushes up the direct engineering cost.

For the remediation of dissolved contaminants, the Permeable Reactive Barrier (PRB) technology, with its advantages of "passive interception - in-situ degradation/adsorption", has become a typical technical approach for in-situ remediation of contaminated groundwater, applicable to both organic pollutants (e.g., PAHs) and heavy metals (e.g., As) using pollutant-specific reactive media. However, its cost is significantly constrained by the spatial and temporal heterogeneity of the medium's adsorption capacity. Based on the coupling analysis of mass conservation

principle and engineering economy, the cost of the PRB system can be quantitatively characterized by formula (S20):

$$C_{\mathrm{PRB}} = \gamma \cdot A_{\mathrm{barrier}} \cdot \rho_{\mathrm{media}} \cdot \frac{\int_0^t \Phi_{\mathrm{PAH}}(t)dt}{Q_{\mathrm{sat}}} \quad (S20)$$

In the formula, C_{PRB}-- The cost of the PRB (Permeable Reactive Barrier) system, which is the expense of constructing and operating this barrier for the purpose of remedying dissolved contamination.

γ--Cost coefficient.

A_{barrier}--The area of a PRB barrier is a two-dimensional coverage parameter of the barrier in space.

ρ_{media}--The density of the medium (the medium material in PRB used for adsorption and other functions).

$\Phi_{\mathrm{PAH}}(t)$-- The flux of the target contaminant (e.g., PAHs for organic pollution scenarios, or As for heavy metal scenarios) varies with time, reflecting the amount of pollutant passing through at different times.

t--The time variable represents the time span from the start of the repair project to the calculation moment.

Q_{sat}--Saturated adsorption capacity.

The cost coefficient γ is the cost conversion coefficient corresponding to the unit pollutant flux adsorption of the unit mass functional medium under the unit barrier area. This coefficient needs to be determined through the comprehensive consideration of multiple factors: on one hand, conduct engineering economic accounting by combining the market price of the functional medium, the construction process quota standards, and the energy consumption and labor costs during the operation and maintenance stage; on the other hand, it is necessary to consider the combined influence of the site engineering conditions (construction difficulty, material transportation distance) and market factors (raw material price fluctuations), which is the key bridge for the transmission of engineering economic elements to cost quantification. A_{barrier} is the intuitive manifestation of the spatial interception range of the PRB system. Its value needs to be determined by combining the horizontal distribution range of the pollutant plume and the direction of groundwater flow. It directly determines the scale cost of the PRB system. The saturated adsorption capacity Q_{sat} reflects the maximum adsorption capacity of the unit mass reactive medium for the target contaminant (e.g., PAHs on

activated carbon, or As on zero-valent iron/iron-oxide media). This study determines Q_{sat} through batch adsorption experiments, combined with the fitting of Langmuir/Freundlich adsorption isothermal models; it not only reflects the static adsorption potential of the medium, but also constrains the service life and medium replacement cost of the PRB system through the matching relationship between the adsorption process and fluid flow.

Formula (S20) quantifies the temporal and spatial consumption degree of the medium's adsorption capacity by the ratio of cumulative pollution flux to the saturated adsorption capacity of the medium; further, by combining the barrier area, medium density and cost coefficient, the pollution load demand, medium technical attributes and engineering economic elements are coupled into a unified cost expression, achieving quantitative correlation across the dimensions of pollution, technology and economy.

When the organic pollution exhibits the characteristics of high flux and strong temporal and spatial fluctuations, $\int_0^t \Phi_{\mathrm{PAH}}(t)dt$ will significantly increase; if the saturation adsorption capacity Q_{sat} of the medium is limited, then it is necessary to meet the remediation requirements by increasing the barrier area A_{barrier}, improving the medium filling density ρ_{media}, or increasing the frequency of medium replacement, etc. Ultimately, this is reflected in the increase of the PRB system cost C_{PRB}. The introduction of the cost coefficient γ further converts the physical responses of technical parameters into economic expressions of engineering costs, making the model have both the physical interpretability of the technical process and the quantitative feasibility of economic assessment. The characterization of the saturation adsorption capacity Q_{sat} of the fluid-medium interaction matches the flow rate of the fluid in the saturated adsorption medium with the adsorption kinetics, reflects the static adsorption capacity and considers the impact of the temporal stability of the medium's adsorption efficiency during the dynamic remediation process on the long-term cost, providing a quantitative basis for the selection of functional media in the PRB system and the optimization of the operation cycle.

Bottom sediment, as an important "sink" for pollutants in water environments, involves not only direct costs such as equipment leasing, earthwork transportation, and pollutant disposal, but also indirect costs (such as the implementation of ecological restoration auxiliary projects and the continuous investment in long-term ecological and

environmental monitoring). The traditional "all-encompassing one-time dredging" model is prone to causing problems such as the destruction of benthic communities and the pulsed release of pollutants due to the neglect of the ecological thresholds of aquatic organisms. This, in turn, leads to the expansion of ecological restoration auxiliary projects (such as artificial habitat reconstruction and the reintroduction of native species) and the extension of monitoring periods, significantly increasing indirect costs. Therefore, it is necessary to optimize the bottom sediment dredging strategy with the core of ecological threshold regulation, achieving ecological control of costs.

The dredging of sediment should be constrained by the threshold for aquatic organism recovery to avoid secondary ecological damage, such as the loss of benthic animal habitats and the toxic impact of pulsed pollutant releases on plankton. Based on the BCIM model of sediment-biota interaction, this study outputs the critical concentration of typical benthic indicator organisms (Limnodrilus hoffmeisteri) as 20 mg/kg. That is, when the sediment pollutant concentration exceeds this value, dredging intervention should be combined; at the same time, to match the "gradual recovery characteristics" of the ecosystem, a phased dredging strategy is adopted: the first stage targets the core contaminated area where the pollutant concentration is far above the critical value and the biological activity is nearly lost, and the second stage focuses on the transitional area where the concentration is close to the critical value and the biological community has preliminary recovery potential. Through "temporal mismatch + spatial partitioning", the instantaneous disturbance to the ecosystem caused by dredging is reduced.

The optimization of dredging depth and area requires a comprehensive consideration of "pollution intensity, hydrodynamic conditions, and biological response", which is quantified by formula (S21):

$$D_{\text{dredge}} = f\left(\frac{C_{\text{sed}} - S_{\text{eco}}}{k_{\text{resusp}} \cdot v_{\text{current}}}\right) \cdot \eta_{\text{bioavoid}} \qquad (S21)$$

In the formula, D_{dredge}——Dredging depth refers to the depth index of the sediment that needs to be excavated when carrying out sediment dredging operations.

C_{sed}——The concentration of pollutants in the sediment.

S_{eco}——Ecological security threshold.

k_{resusp}——Resuspension coefficient.

v_{current}——Velocity of water.

η_{bioavoid}——Biological avoidance behavior correction factors.

The ecological safety threshold S_{eco} refers to the critical value at which the concentration of pollutants in the sediment does not cause a significant decline in the aquatic biological community. To ensure the safety of aquatic ecosystems, the maximum allowable concentration of the corresponding substances in the sediment is permitted. Exceeding this limit may have adverse effects on the ecology. The re-suspension coefficient k_{resusp} represents the ease or proportion coefficient of pollutants re-suspending into the water body due to the action of water flow and other factors, describing the difficulty of sediment particles (containing pollutants) entering the overlying water body under water flow disturbance. The water flow velocity v_{current} influences the migration and diffusion behaviors of the sediment and pollutants. The higher the velocity, the easier the sediment is to be disturbed, and the re-suspension coefficient k_{resusp} shows a positive correlation response. The bioavoid correction factor η_{bioavoid} uses to correct the behavior of aquatic organisms avoiding contaminated sediment and other behaviors. Benthic organisms (such as Limnodrilus hoffmeisteri) will avoid high-pollution sediment through "vertical migration and horizontal escape". This factor quantifies the degree of correction of the range of contaminated sediment that needs dredging by the active behavior of the organisms, making the dredging plan more in line with the ecological reality.

S16.3 Assessment of losses during the recovery period of ecosystem functions

The two-stage S-shaped recovery curve model used in this study is based on the ecological succession theory proposed by Odum[11] and has been verified in a large number of aquatic ecosystem restoration cases[12].

After an ecosystem is severely disturbed, its functional recovery usually exhibits significant time-lag effects and nonlinear dynamic characteristics. This process directly determines the extent of loss of ecological service functions during the recovery period. Traditional static assessment methods cannot reflect the phased differences and asynchrony of service functions during the recovery process, which may lead to underestimation or overestimation of the losses. Therefore, establishing a dynamic quantitative framework that can depict the time series characteristics of the recovery process and integrate the asynchrony of ecological functions is of great significance for scientifically assessing the loss of ecosystem service functions and optimizing

restoration decisions. This section takes the water-body-water-invertebrate coupled system as the object, based on the recovery threshold theory, the functional lag model, and the opportunity cost algorithm, proposes a "recovery path - functional lag - economic discount" tripartite dynamic loss assessment system, and demonstrates its applicability and scientificity in the actual restoration process through the case of the blue-green algae control project in Taihu Lake. The dynamic quantitative framework constructed in this study includes three core modules:

1）Recovery path module: Describe the recovery trajectory of key ecological factors. Use a two-stage S-shaped curve to simulate the recovery process of submerged vegetation coverage, which can depict the two stages of rapid recovery and slow recovery, and introduce the ecological memory factor to reflect the response inertia of the ecosystem after disturbance.

2）Functional lag module: In response to the asynchronous nature of ecological service functions, by combining the recovery trajectory of benthic community structure (the change in Shannon index predicted by the BCIM model) and the changes in water body nitrogen and phosphorus removal rates, the differences in the main functional recovery times are quantified, and the impact of functional lag effects on the overall service value is depicted.

3）Economic discount module: Based on the theory of opportunity cost and the discounted cash flow method, the unrealized service function value during the recovery period is converted to its present value to reflect the long-term economic impact of the loss during the recovery period.

The above framework achieves a multi-dimensional integration from ecological structure recovery to functional response and then to economic value assessment, and is applicable to typical cases such as river basin water ecosystems. Submerged vegetation, as an important primary producer in lake ecosystems, plays a decisive role in the improvement of water quality and the reconstruction of benthic biological communities. Based on the measured data and related restoration dynamics research, the coverage of submerged vegetation shows a two-stage recovery characteristic after disturbance, and the recovery process is modeled by a two-stage S-shaped curve model as shown in formula (S22):

$$R_t = R_\infty\left[1 - e^{-\kappa_1 t} \cdot (1 - \beta) + \beta e^{-\kappa_2 (t - t_c)}\right] \qquad (S22)$$

In the formula, R_t--The coverage of submerged vegetation at time t.

R_∞--The progressive recovery level, 82%, characterizes the coverage of the ultimate goal of recovery.

κ_1--The rate constant during the rapid recovery phase is set at 0.18 per month ($month^{-1}$), corresponding to the period from 0 to 8 months, representing the initial rate of recovery.

t_c--Critical time point is set at 8 months, indicating the transition point of the recovery stage.

κ_2--The rate constant for the slow recovery stage is set at 0.05 per $month^{-1}$, representing the rate during the later stage of recovery.

β--The ecological memory factor is set at 0.35, which is used to quantify the "memory" effect of the ecosystem and influence the smoothness of the transition stage.

t--Time, month.

This model can reflect ecological characteristics: 1) The phased nature of the recovery rate: The recovery rate is fast in the early stage and gradually slows down in the later stage.; 2) Ecological memory effect: By controlling the smoothness of the recovery curve with β, the ecosystem will exhibit response inertia after disturbance, causing the recovery path to deviate from a single S-shaped curve; 3) Compatibility with the BCIM model: The benthic organism Shannon index predicted by BCIM shows a synergistic relationship with the vegetation recovery process, but there is a significant time lag, and asynchronous analysis needs to be introduced in the framework.

After an ecosystem is disturbed, it enters a recovery period. The formation of economic losses in such a situation is not solely due to the absence of a single service supply, but rather a coupled and cumulative process of functional deficiency costs and opportunity costs. This dual-dimensional composition not only reflects the reality of the current value loss of ecological services, but also embodies the possibility of potential loss of the potential benefits from resource allocation. It is the core logic for understanding the profit and loss characteristics of the dual system of ecology and economy during the recovery process.

Functional deficiency costs arise from the current value loss due to the ecosystem's service functions not reaching the baseline level (the healthy state without disturbance). Essentially, it is the total value of services that the ecosystem should have provided but failed to do so due to the lag in recovery. Its measurement needs to be carried out by coupling the two dimensions of the continuity of ecological processes and the temporal

dynamics of value:

1）Ecological process dimension: The Source and Quantification of Functional absence

The recovery trajectories of ecosystem service functions and core structural factors are highly coordinated. Define the recovery degree at any time t as $\frac{R_t}{R_\infty}$ (where R_t is the submerged vegetation coverage at time t, R_∞ is the target coverage, representing the structural state when the function is fully restored); then the functional deficiency rate can be expressed as $1-\frac{R_t}{R_\infty}$, which directly quantifies the proportion of the loss of ecosystem service functions at time t compared to the baseline level - this is the direct source of the cost of functional deficiency.

2）Value measurement dimension: Dynamic discounting and cumulative accounting

Baseline service value (V_{base}): This represents the total economic value of the integrated services provided by the ecosystem over a unit of time (such as on an annual scale) in a healthy state, covering various dimensions of services such as water purification, fishery output, and biodiversity maintenance. It needs to be assessed through a multi-method integration approach (such as the market value method, the substitution cost method, etc.) for determination.

Time value discounting: Incorporation of the social discount factor $e^{-\delta t}$ (where δ is the social discount rate), converting the loss of services in future moments into current present value, reflecting the time value of money and social preferences. The impact of a 1-unit loss of value at the present moment on economic decision-making is significantly greater than that of a 1-unit loss of value in the future.

The value loss due to the absence of functions needs to be dynamically calculated by combining the baseline ecosystem service value with the discounting of time value. Therefore, the present value of the cost of function absence can be achieved through an integral form, "cumulatively measuring the value of service absence in the continuous time dimension". The mathematical expression is as shown in formula (S23):

$$L_{\text{rec}} = \int_{t_0}^{t_f} \left[V_{\text{base}} \cdot \left(1-\frac{R_t}{R_\infty}\right) \cdot e^{-\delta t} \right] dt \cdot \gamma_{\text{opp}} \qquad (S23)$$

In the formula, L_{rec}——Cumulative economic losses during the recovery period.

V_{base}——Baseline ecosystem service value.

R_t——The vegetation coverage at time t.

R_∞——Target coverage.

$\frac{R_t}{R_\infty}$——Restored degree.

$1 - \frac{R_t}{R_\infty}$——The function deficiency rate indicates the proportion of the loss of ecosystem service functions at time t compared to the baseline level. This is the direct cause of the current loss.

δ——social discount rate.

$e^{-\delta t}$——Discount factor, used to calculate the present value of future losses at the current time. The longer the time period t, the smaller this factor becomes, indicating that the present value of future losses is lower.

γ_{opp}——Opportunity cost correction factor.

Opportunity cost arises from the occupation of resources by ecological restoration activities, resulting in the potential loss of returns that could have been generated from these resources being diverted to other productive activities. Ecological restoration requires the allocation of scarce resources such as funds, land, technology, and human resources. If these resources were allocated to other economic sectors (such as industrial development and infrastructure construction), they could have generated corresponding investment returns. Therefore, the essence of opportunity cost is the cost of suboptimal resource allocation. The opportunity cost correction factor γ_{opp} reflects the loss of investment returns due to resource idleness, γ_{opp} is a coefficient greater than 1, used to correct and quantify the potential losses in returns that would have been incurred if the resources were not occupied (such as for ecological restoration) and instead were invested in other productive activities.

The value of factor γ_{opp} needs to be comprehensively considered across three dimensions: 1) Regional resource scarcity: the scarcer the land resources are, the higher the opportunity cost of using them for ecological restoration; 2) The return on investment of alternative production activities: the higher the average return on investment of local advantageous industries, the higher the opportunity cost of shifting resources to ecological restoration; 3) The specificity of ecological restoration resources: the more difficult it is to transfer resources from the ecological restoration field to other production activities, the lower the relative opportunity cost.

Through γ_{opp}, the cost of functional deficiency can be associated with the

potential loss of benefits, that is, the measurement of opportunity cost is based on the cost of functional deficiency, and the cost of resource misallocation is amplified through a correction factor. The total economic loss during the recovery period is the coupled superposition of the cost of functional deficiency and the opportunity cost. This coupling relationship profoundly reflects the complexity of economic losses during the recovery period: The cost of functional deficiency is the direct manifestation of the insufficient supply of ecosystem services, reflecting the loss of service flow within the ecosystem. The opportunity cost is an indirect reflection of external resource allocation constraints, reflecting the cost of resource misallocation in the social economic system. The superposition of the two will integrate the internal gains and losses of the ecosystem and the resource allocation gains and losses of the social economic system into the loss accounting system, completely depicting the collaborative loss characteristics of the ecological-economic dual system during the recovery process.

The precise calculation of dual-dimensional costs relies not only on the dynamic quantification ability of ecological processes, but also requires the integration of multiple methods for the assessment of regional social and economic characteristics (such as the multi-method integration of V_{base} and the multi-dimensional parameter calibration of γ_{opp}). Only by achieving the cross-system coupling and quantification of "ecological processes - economic value - resource allocation" can we provide scientific basis for the cost-benefit analysis of ecological restoration and the design of ecological compensation mechanisms.

S16.4 Comprehensive assessment and analysis

In the aforementioned section, a quantitative assessment method for water pollution damage was constructed from three dimensions: the impairment of ecosystem service functions, the costs of environmental restoration/governance, and the loss during the recovery period of ecosystem functions. However, a single-dimensional analysis is insufficient to fully reflect the systematic and dynamic nature of pollution events, nor can it effectively support the optimization of restoration funding allocation, policy formulation, and judicial decisions. Therefore, in this section, a comprehensive assessment method based on multi-dimensional coupling is proposed. A "ecological response - economic discount - engineering feedback" full life cycle closed-loop model is constructed to achieve quantitative assessment of the damage, restoration, and recovery process of pollution events.

The theoretical basis of the comprehensive assessment lies in unifying the evaluation scales of ecological, engineering and economic indicators. Firstly, the biological endpoint response data output by the Water Body - Aquatic Organism Comprehensive Assessment Model (BCIM) is coupled with the ecosystem service function damage formula (S15) and the ammonia nitrogen degradation rate loss formula (S16) (S17) to form an ecological damage base matrix. This matrix not only includes the biomass loss of benthic organisms, fish and other secondary consumers, but also includes information on the degradation of microbial self-purification functions, achieving multi-scale integration from the micro community to the macro ecosystem function. Secondly, the environmental restoration and governance cost formulas (S19) to (S22) are incorporated into the time-dependent discounted cash flow system, and combined with the loss formula of the recovery period of ecosystem functions (S23) to calculate the cross-period economic impact, forming a full-cycle quantitative framework. Thus, the comprehensive economic loss C_{total} can be expressed by formula (S24):

$$C_{total} = D_t + C_{repair} + L_{rec} \qquad (S24)$$

In the formula, D_t --The cumulative discounted value of ecosystem service function impairment (formula (S18)) reflects the temporal dynamic loss of ecosystem functions caused by pollution events.

C_{repair}--The total cost of environmental restoration and governance, including direct engineering expenses and indirect long-term monitoring costs (formulas (S19) to (S22)).

L_{rec}--Loss of ecosystem function recovery period (formula (S23)).

Through this expression, the entire economic aspect of the pollution incident can be quantitatively integrated within the same framework, achieving consistency and comparability in cross-departmental management, ecological compensation, and judicial decisions.

In the quantification of ecological damage, this study proposes a multi-path weighted method to take into account the different ecological service functions and their recovery time characteristics. Specifically, weights w_s and w_r are assigned to supply services (such as fishery productivity, submerged vegetation coverage) and regulating services (such as nitrogen and phosphorus removal, water purification capacity), respectively. Combined with the decline rate of benthic organisms and the

degradation rate of microbial self-purification function, a comprehensive ecological damage index EDI is formed as expressed in formula (S25):

$$EDI = w_s \cdot \sum_i \Delta E_{fish,i} + w_r \cdot \sum_j E_j \qquad (S25)$$

In the formula, $\Delta E_{fish,i}$ -- The economic loss of the i-th type of secondary consumers (formula (S15)).

E_j-- The loss amount of the j-th type of regulating service (formula (S17)).

w_s and w_r —Weight. The weights were assigned according to the relative contribution of provisioning and regulating services to the total economic value of aquatic ecosystem services, based on the ecosystem service value equivalent factor method[8, 13] and expert consultation. For riverine aquatic ecosystems, regulating services (water purification, waste treatment, hydrological regulation) account for a substantially larger share of total service value than provisioning services (fishery production, water supply); accordingly, w_s = 0.4 and w_r = 0.6 were adopted. The weights can be adjusted for different water body types and pollution scenarios based on local ecosystem service valuation. The EDI classification thresholds are defined as follows: EDI < 50, mild damage (weighted loss < ~8% of the annual baseline value V_0); 50 ≤ EDI < 200, moderate damage (~8–30% of V_0); EDI ≥ 200, severe damage (> ~30% of V_0). The 30% threshold for severe damage is consistent with the ecological risk assessment principle that loss of more than 30% of a key ecosystem function indicates severe impairment. This index can simultaneously reflect the spatial heterogeneity, functional asynchrony of the ecosystem services, and the multi-path effects of pollution stress, providing a scientific basis for prioritizing restoration strategies and allocating funds.

In terms of integrating the costs of environmental restoration, this study established a time-dynamic coupling model, taking into account the decline in technical efficiency, the reduction in medium adsorption capacity, and the ecological feedback effect. Taking the PRB system as an example, the dynamic changes in its restoration costs over time (t) can be represented by an attenuation function (S26):

$$C_{PRB}(t) = C_{PRB,0} \cdot \left(1 - \eta_{decay} \cdot t\right) \qquad (S26)$$

In the formula, $C_{PRB,0}$-- Initial construction and operation costs.

η_{decay}-- The annual average attenuation rate of adsorption capacity reflects the

characteristic that the performance of the remediation medium declines over time.

Similarly, the nonlinear relationship between the dosage of the drug and the volume of pollution, as well as the reaction kinetics attenuation coefficient β, in the ISCR technology can be used to real-time correct the costs, thereby avoiding the deviations caused by static cost estimation.

The comprehensive assessment of the loss during the recovery period of ecosystem functions also adopts a dynamic approach. Based on the double-stage S-shaped recovery curve, the asynchronous recovery of submerged vegetation coverage, benthic biodiversity, and water purification functions are incorporated into a unified framework, and are mapped through functions to the annual economic loss L_{rec}. Combined with the social discount rate δ and the opportunity cost correction coefficient γ_{opp}, the future recovery period losses are converted into present values, enabling comparable analysis of losses across different time periods.

The application process of the comprehensive assessment framework can be summarized into three steps: Firstly, conduct quantitative assessment of ecological responses, based on the output of the BCIM model, calculate the functional losses of benthic organisms, secondary consumers and microbial communities, and construct the comprehensive ecological damage index EDI. Secondly, perform dynamic integration of engineering costs, apply time decay and efficiency correction to the cost of restoration technologies, combine long-term monitoring and auxiliary engineering expenses to form the dynamic cost matrix $C_{repair}(t)$. Finally, conduct discounting of losses during the recovery period, using the double-stage S-shaped curve and opportunity cost coefficient, convert the losses during the ecological function recovery period into present value, and combine uncertainty analysis to assess the risk premium. Therefore, the total comprehensive assessment value C_{total} can be used to quantitatively describe the full life cycle impact of pollution events on the aquatic ecosystem, providing theoretical support for the optimization of restoration strategies, capital allocation and liability determination.

Table S12 summarizes all parameters used in the economic assessment, together with their values, units, sources, and rationale.

Table S12. Sources, values, units, and rationale of economic assessment parameters.

Parameter	Symbol	Value	Unit	Source / Rationale
Social discount rate	r	3.5	%	[14]
Baseline ecosystem service value	V_0	650	10^4 CNY/year	Equivalent factor method[8, 13]
Trophic transfer efficiency	η	0.12	dimensionless	Lindeman efficiency (~10%),[11]
Thermodynamic loss coefficient	TLE	1.8	dimensionless	Energy flow analysis, calibrated from field data
Fish market price	p_f	1200	CNY/t	Local fishery market price
Time decay constant	τ	8.3	month	Calibrated from post-accident recovery monitoring
Hill coefficient	n	1.7	dimensionless	Fitted from BCIM model output
Artificial water purification cost	C_{vol}	1.0	CNY/m^3	Local wastewater treatment cost
Reducing agent (sodium dithionite) price	—	8000	CNY/t	Market price; US EPA Remediation Technology Cost Manual
ISCR reaction efficiency coefficient	α	—	dimensionless	Laboratory batch and field pilot tests
ISCR reaction kinetics decay coefficient	β	—	dimensionless	Field calibration
Hydraulic conductivity	K_h	18	m/d	Field hydrogeological survey
Dredging ecological safety threshold	S_{eco}	20	mg/kg	BCIM model output (Limnodrilus avoidance threshold)
Recovery target (submerged vegetation)	R_{max}	82	%	field restoration target[12]
Rapid recovery rate	k_1	0.18	month^{-1}	Two-stage S-curve fitting, 0–8 months
Critical recovery time	t_c	8	month	Field monitoring data
Slow recovery rate	k_2	0.05	month^{-1}	Two-stage S-curve fitting, >8 months
Ecological memory factor	β_{eco}	0.35	dimensionless	calibrated from recovery trajectory[11]
Opportunity cost correction factor	k_{opp}	—	dimensionless	Regional resource scarcity and alternative ROI

Parameter	Symbol	Value	Unit	Source / Rationale
EDI weight, provisioning services	w_1	0.4	dimensionless	Equivalent factor method + expert consultation
EDI weight, regulating services	w_2	0.6	dimensionless	Equivalent factor method + expert consultation
EDI severe-damage threshold	—	200	10^4 CNY	>30% of annual baseline value V_0
Maximum inhibition rate (Cr(VI))	$V_{max,Cr}$	0.30	d^{-1}	Literature on Cr(VI) inhibition of nitrifying bacteria[15,16]
Half-effect concentration (Cr(VI))	$K_{m,Cr}$	0.50	mg/L	Literature EC50 for Cr(VI) nitrification inhibition[15,16]
Representative Cr(VI) concentration	C_{Cr}	0.35	mg/L	SWAT model prediction, 24-h peak in contaminated reach
Inhibition development coefficient	γ	0.02	d^{-1}	Fitted from BCIM model output
Contaminated river reach volume	V	1.8×10^5	m^3	6 km × 15 m × 2 m; from SWAT river geometry
Assessment time	t	180	d	Post-accident assessment period

References

[1] BEAR J. Dynamics of fluids in porous media [J]. 1972.

[2] ZHENG C, BENNETT G D. Applied contaminant transport modeling [M]. Wiley-Interscience New York, 2002.

[3] DAVIS J A, JAMES R O, LECKIE J O J J O C, et al. Surface ionization and complexation at the oxide/water interface: I. Computation of electrical double layer properties in simple electrolytes [J]. 1978, 63(3): 480-99. http://dx.doi.org/https://doi.org/10.1016/S0021-9797(78)80009-5

[4] PROMMER H, BARRY D A, ZHENG C. MODFLOW/MT3DMS-based reactive multicomponent transport modeling [J]. Ground Water, 2003, 41(2): 247-57. http://dx.doi.org/10.1111/j.1745-6584.2003.tb02588.x

[5] ARNOLD J G, SRINIVASAN R, MUTTIAH R S, et al. Large Area Hydrologic Modeling and Assessment Part I: Model Development1 [J]. JAWRA Journal of the American Water Resources Association, 1998, 34(1): 73-89. http://dx.doi.org/10.1111/j.1752-1688.1998.tb05961.x

[6] NEITSCH S L, ARNOLD J G, KINIRY J R, et al. Soil and water assessment tool theoretical documentation version 2009[R]. Texas Water Resources Institute, 2011.

[7] COLE D L, RUIZ-MERCADO G J, ZAVALA V M. A graph-based modeling framework for tracing hydrological pollutant transport in surface waters [J]. Computers

& Chemical Engineering, 2023, 179. http://dx.doi.org/10.1016/j.compchemeng.2023.108457
[8] COSTANZA R, DARGE R, DEGROOT R, et al. The value of the world's ecosystem services and natural capital [J]. Nature, 1997, 387(6630): 253-60. http://dx.doi.org/10.1038/387253a0
[9] EUROPEAN COMMISSION. Guidance Document on the Assessment of Environmental Damage[R]. Brussels, 2010.
[10]U.S. ENVIRONMENTAL PROTECTION AGENCY. Remediation Technology Cost Manual[R]. EPA/542/R-20/001. Washington, DC: 2020.
[11]ODUM E J S. The strategy of ecosystem development [J]. 1969, 164(3877): 262-70. http://dx.doi.org/10.1126/science.164.3877.262
[12]PALMER M A, BERNHARDT E S, ALLAN J D, et al. Standards for ecologically successful river restoration [J]. Journal of Applied Ecology, 2005, 42(2): 208-17. http://dx.doi.org/10.1111/j.1365-2664.2005.01004.x
[13]XIE G, ZHANG C, ZHEN L, et al. Dynamic changes in the value of China's ecosystem services [J]. Ecosystem Services, 2017, 26: 146-54. http://dx.doi.org/10.1016/j.ecoser.2017.06.010
[14]DEVELOPMENT N, REFORM COMMISSION, MINISTRY OF CONSTRUCTION. Methods and Parameters for Economic Evaluation of Construction Projects [M]. 3rd ed. Beijing: China Planning Press, 2006.
[15] Su X, Zhai H, Ouyang F, et al. Responses of activities and communities of nitrifying bacteria to chromium (VI) in activated sludge [J]. Desalination and Water Treatment, 2016, 57(24): 11111-11120. https://doi.org/10.1080/19443994.2015.1044913
[16] Kim Y M, Park H, Chandran K. Nitrification inhibition by hexavalent chromium Cr (VI)–Microbial ecology, gene expression and off-gas emissions[J]. Water research, 2016, 92: 254-261. https://doi.org/10.1016/j.watres.2016.01.042
